# Genomic analysis of the domestication and post-Spanish conquest evolution of the llama and alpaca

**DOI:** 10.1186/s13059-020-02080-6

**Published:** 2020-07-02

**Authors:** Ruiwen Fan, Zhongru Gu, Xuanmin Guang, Juan Carlos Marín, Valeria Varas, Benito A. González, Jane C. Wheeler, Yafei Hu, Erli Li, Xiaohui Sun, Xukui Yang, Chi Zhang, Wenjun Gao, Junping He, Kasper Munch, Russel Corbett-Detig, Mario Barbato, Shengkai Pan, Xiangjiang Zhan, Michael W. Bruford, Changsheng Dong

**Affiliations:** 1 College of Animal Science and Veterinary Medicine, Shanxi Agricultural University, Taigu, Shanxi China; 2 CAS Key Lab of Animal Ecology and Conservation Biology, Institute of Zoology, Chinese Academy of Sciences, Beijing, China; 3 Cardiff University – Institute of Zoology Joint Laboratory for Biocomplexity Research, Chinese Academy of Sciences, Beijing, China; 4 University of Chinese Academy of Sciences, Beijing, China; 5 BGI Genomics, BGI, Shenzhen, China; 6 Departamento de Ciencias Básicas, Facultad de Ciencias, Universidad del Bio Bio, Chillán, Chile; 7 Programa de Doctorado en Ciencias mención Ecología y Evolución, Escuela de Graduados, Facultad de Ciencias., Universidad Austral de Chile, Valdivia, Chile; 8 Facultad de Ciencias Forestales y de la Conservación de la Naturaleza, Universidad de Chile, Santiago, Chile; 9 CONOPA-Instituto de Investigación y Desarrollo de Camélidos Sudamericanos, Pachacamac, Lima, Peru; 10 Bioinformatics Research Centre, Aarhus University, Aarhus, Denmark; 11 Department of Biomolecular Engineering and Genomics Institute, UC Santa Cruz, Santa Cruz, CA USA; 12 Department of Animal Science, Food and Technology – DIANA, Università Cattolica del Sacro Cuore, Piacenza, Italy; 13 Center for Excellence in Animal Evolution and Genetics, Chinese Academy of Sciences, Kunming, China; 14 School of Biosciences and Sustainable Places Institute, Cardiff University, Cardiff, Wales UK

**Keywords:** Introgression, Domestication, Llama, Alpaca, Spanish conquest

## Abstract

**Background:**

Despite their regional economic importance and being increasingly reared globally, the origins and evolution of the llama and alpaca remain poorly understood. Here we report reference genomes for the llama, and for the guanaco and vicuña (their putative wild progenitors), compare these with the published alpaca genome, and resequence seven individuals of all four species to better understand domestication and introgression between the llama and alpaca.

**Results:**

Phylogenomic analysis confirms that the llama was domesticated from the guanaco and the alpaca from the vicuña. Introgression was much higher in the alpaca genome (36%) than the llama (5%) and could be dated close to the time of the Spanish conquest, approximately 500 years ago. Introgression patterns are at their most variable on the X-chromosome of the alpaca, featuring 53 genes known to have deleterious X-linked phenotypes in humans. Strong genome-wide introgression signatures include olfactory receptor complexes into both species, hypertension resistance into alpaca, and fleece/fiber traits into llama. Genomic signatures of domestication in the llama include male reproductive traits, while in alpaca feature fleece characteristics, olfaction-related and hypoxia adaptation traits. Expression analysis of the introgressed region that is syntenic to human HSA4q21, a gene cluster previously associated with hypertension in humans under hypoxic conditions, shows a previously undocumented role for PRDM8 downregulation as a potential transcriptional regulation mechanism, analogous to that previously reported at high altitude for hypoxia-inducible factor 1α.

**Conclusions:**

The unprecedented introgression signatures within both domestic camelid genomes may reflect post-conquest changes in agriculture and the breakdown of traditional management practices.

## Background

South and Central America encompasses one of the most important cradles of domestication, giving rise to key crop plants and domesticated animals including turkey, guinea pig, and the region’s key domestic herbivores, the llama and alpaca [1]. These South American camelids (SACs) attained central importance in the economy of the Andean region, with the llama used as a pack animal, facilitating the expansion of the Inca Empire [2], and the alpaca selected to produce some of the world’s finest wool [3]. Both putative wild ancestors (the guanaco and vicuña) remain extant, with SACs classified in two genera, each containing two species: *Lama guanicoe* (guanaco) and *L*. *glama* (llama), and *Vicugna vicugna* (vicuña) and *V*. *pacos* (alpaca) [4, 5]. The alpaca has been variously hypothesized as descending from the guanaco, the vicuña, and as a llama/vicuña hybrid, while the llama is generally thought to originate from the guanaco [4]. These hypotheses stem from studies of morphological and behavioral variations in extant animals, while archaeozoological evidence places domestication of the alpaca from the vicuña in the wet puna ecosystem of Peru’s central Andes, 6000–7000 years ago, and domestication of the llama from the guanaco in the dry punas of southern Peru, Chile, and Argentina [6]. Previous molecular data, while confirming that the genera *Lama* and *Vicugna* are valid and separated 2–3 MYA (million years ago), have documented extensive hybridization among modern domestic SACs, with microsatellite and mitochondrial DNA analysis suggesting that the llama is descended from the guanaco and the alpaca is descended from the vicuña, and the alpaca has been reclassified from *Lama pacos* to *Vicuna pacos* [4, 5]. Recent Y-chromosome analysis has recapitulated this result [7]. A genomic perspective on this problem is urgently needed, however, because previous analysis used a few neutral genetic markers, representing only a very small fraction of the genome, and was incapable of accurately assessing genetic diversity, genomic regions under selection and systematically evaluating genome-wide introgression in wild and domestic forms.

We generated de novo genomes and transcriptomes for vicuña, llama, and guanaco, adding to a reference genome previously produced for alpaca [8] and resequenced the genomes of seven further individuals of each species. We aimed to elucidate the following for the llama and alpaca: (1) their evolutionary origins; (2) the level, origin, distribution of introgression in both species; (3) their demographic history from domestication to the present day, including the Spanish conquest; and (4) genes selected during domestication and potentially as a result of admixture, and their implication for the llama and alpaca’s unique adaptations.

## Results

### De novo sequencing, assembly, and resequencing

Genomes were sequenced to 129x, 108x, and 102x coverage for *L*. *guanicoe*, *V*. *vicugna*, and *L*. *glama*, respectively (Additional file 1: Supplementary Text 1a, b and Additional file 1: Table S1); all these genomes were assembled by SOAPdenovo [9] and featuring contig and scaffold N50 lengths of 91.6 kb and 14.6 Mb for *L*. *guanicoe*, 91.1 kb and 6.15 Mb for *V*. *vicugna*, and 44.1 kb and 3.2 Mb for *L*. *glama* (Additional file 1: Table S2a-c). Each species’ blood was used for RNA sequencing and those assembled unigene alignment showed that the assemblies covered more than 82% of unique genes for each species (Additional file 1: Table S3). Benchmarking Universal Single-Copy Orthologs (BUSCO) assessment showed that > 93.7% of core genes [10] could be aligned to the SAC genomes (Additional file 1: Table S4). The three de novo genomes were all inferred to be ~ 2.6 Gb (Additional file 1: Table S5 and Figure S1) and share high synteny with the human and *V*. *pacos* reference genomes (coverage rate 87%; Additional file 1: Table S6), with a relatively low rate of genomic rearrangement. We identified 21,435 protein-coding genes in the genomes of *L*. *guanicoe*, 21,757 in *V*. *vicugna*, and 21,460 in *L*. *glama*, respectively (Additional file 1: Table S7a-c). BUSCO assessment showed that the large majority present in the predicted gene sets (over 78%). In total, > 92% of genes was functionally annotated. Repeat sequences were inferred to comprised 33.1% of the genome in *L*. *guanicoe*, 32.7% in *V*. *vicugna*, and 27.02% in *L*. *glama*, lower values than in most mammals [11], although such sequences were difficult to assemble most likely due to the limitations of short-read sequencing. Resequencing of seven individuals per species (Additional file 1: Figure S2; Table S8) yielded an average sequence depth of 16-fold after filtering (31–45 Gb, Additional file 1: Table S9). Resequencing uncovered 21.3 million SNPs and a heterozygous SNP rate of 0.0030 for alpaca, 25.2 million and 0.0023 for vicuña, 16.7 million and 0.0018 for guanaco, and 16.1 million and 0.0020 for llama, indicating a higher overall SNP rate for the *Vicugna* lineage.

### Phylogenetic, admixture, and lineage sorting analysis

**Fig. 1:**
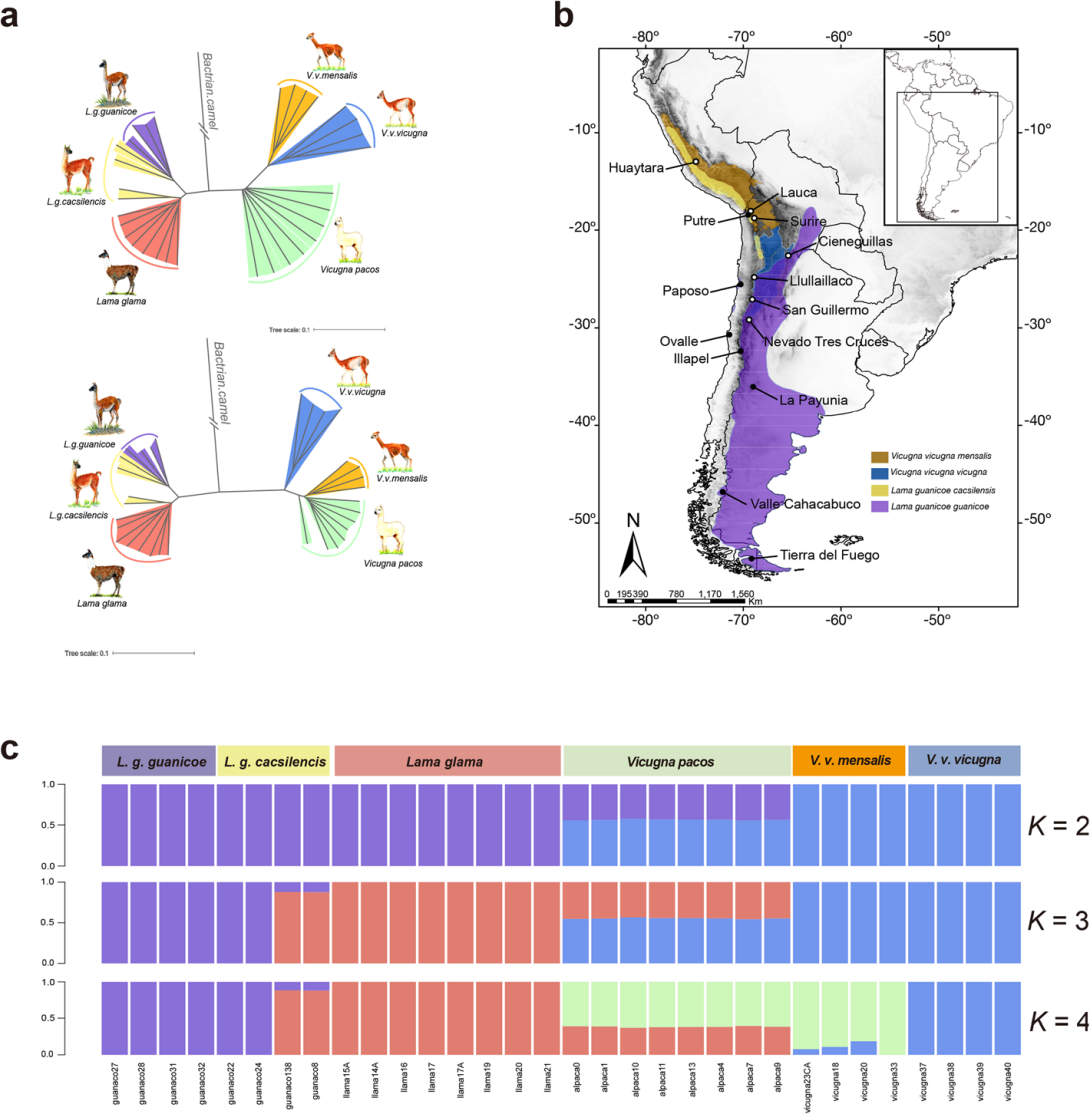
Evolutionary relationship among South American camelids (SACs). **a** Neighbor-joining (NJ) trees by using all whole-genome data (top) and by using loci located in alpaca regions of highest vicuña ancestry (bottom). **b** Map with current distributions of SACs and sample location in this study. Open and solid dots indicate sampled localities of vicuña and guanacos respectively. **c** Model-based clustering of 32 SACs performed using ADMIXTURE with the number of ancestry kinships (*k*) set to 2, 3, or 4. Each vertical bar in proportions is corresponding to its proportion of genetic ancestry from each of these ancestral populations. The names of species and samples are shown in top and below of figure respectively

A neighbor-joining tree based on 5,901,447 SNPs (Fig. 1a upper) showed two major SAC lineages with 100% bootstrap support, comprising vicuña and alpaca in one group (genus *Vicugna*) and guanaco and llama in the other (genus *Lama*). Within *Lama*, *L*. *g*. *guanicoe* formed a single group, whereas two *L*. *g*. *cacsilensis* (guanaco 22 and 24) grouped with *L*. *g*. *guanicoe*, the other two (guanaco138DA and 8) grouped with all llamas. Guanaco 22 and 24 were collected at Paposo and Ovalle, Chile, respectively 7° and 11° latitude further south than guanaco138DA and 8, collected in Putre, northern Chile (Fig. 1b; Additional file 1: Figure S2). Guanaco 22 and 24 are therefore likely mislabelled as *L*. *g*. *cacsilensis* since they were collected within the “dry diagonal,” an arid region and known biogeographic transition zone in the Andean chain [12, 13]. All llamas grouped with the two north Chilean *L*. *g*. *cacsilensis*, supporting the hypothesis that llamas were domesticated from within the *L*. *g*. *cacsilensis* gene-pool. In contrast, both currently described vicuña subspecies clustered separately, with 100% bootstrap support. *V*. *v*. *mensalis* were sampled in northern Chile and Peru, whereas *V*. *v*. *vicugna* were sampled in Chile and Argentina. Despite clustering within *Vicugna*, and with 100% bootstrap support, the alpacas did not cluster within either the *V*. *v*. *mensalis* or *V*. *v*. *vicuna* groups, instead forming a set of apparently divergent lineages with longer branch lengths, nested within the broader *Vicugna* cluster. However, after Local Ancestry Inference (LAI)-assisted removal of all alpaca genomic regions not having significant vicuña ancestry (Fig. 1a lower, see below), the relative position of alpaca changed to group with *V*. *v*. *mensalis*, consistent with a northern origin of domestication of the alpaca as previously reported [3, 7].

Admixture cross-validation analysis showed the optimal grouping to be *k* = 2 (CV = 0.593, 0.620, 0.657, 0.744 for *k* = 2, 3, 4, 5 respectively). At *k* = 2, the results were broadly concordant with the neighbor-joining tree (Fig. 1c), with llama clustering with guanaco and alpaca with vicuña. However, alpaca showed evidence for strong admixture with approximately 36% of genomic SNPs detected in the genomes of all individuals analyzed clustering with guanaco/llama. At *k* = 4 (Fig. 1c), the signal of admixture in the alpaca was still strongly evident, with the remainder of the genome clustering with *V*. *v*. *mensalis*, in accordance with Fig. 1a lower, and evidence of unidirectional admixture from llama to alpaca and from *V*. *v*. *vicuna* into *mensalis*. In addition, also in support of the neighbor-joining results, *L*. *g*. *cacsilensis* samples guanaco138DA and 8 clustered with all llamas (albeit with some evidence of admixture). *Treemix* results (Additional file 1: Figure S3) confirmed both the close relationship between *V*. *pacos* and *V*. *v*. *mensalis* and the unidirectional gene flow between llama and alpaca.

**Fig. 2:**
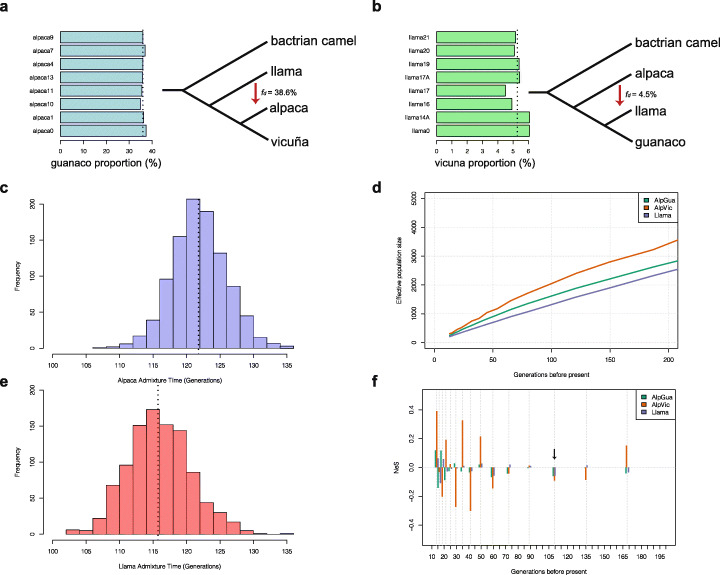
Inferences of admixture proportion and time and demographic history. **a** Estimated introgression proportions from llama to alpaca by using Local Ancestry Inference (LAI) (left). The *X*-axis indicates the guanaco-ancestry proportions in each sequenced alpaca individuals (*Y*-axis; *N* = 8). The right panel showed the introgression proportions by using ABBA-BABA (*f*_*d*_) method. The arrow showed the introgression direction from llama to alpaca. **b** Estimated introgression proportions from alpaca to llama by using LAI (left). The *X*-axis indicates the vicuna-ancestry proportions in each sequenced llama individuals (*Y*-axis; *N* = 8). The right panel showed the introgression proportions by using ABBA-BABA (*f*_*d*_) method. The arrow showed the introgression direction from alpaca to llama. **c** Inference of admixture time in alpaca by analyzing the LAI tract length. Dotted line indicates the median value of admixture time. **d** Change in linkage disequilibrium estimated *N*e using SNeP over approximately the last 200 generations (AlpGua: alpaca genome that removed guanaco ancestry sites; AlpVic: alpaca genome that only remain vicuña ancestry sites). **e** Inference of admixture time in llama by analyzing the LAI tract length. Dotted line indicates the median value of admixture time. **f** Variation in estimated *N*e compared to the overall decline slope, with changes below the 0 value on the *Y*-axis indicating a steeper decline and above the line indicating a shallower decline. The steeper decline event at 110 generations BP (black arrow) coincides with the date of admixture identified by LAI tract analysis

**Fig. 3:**
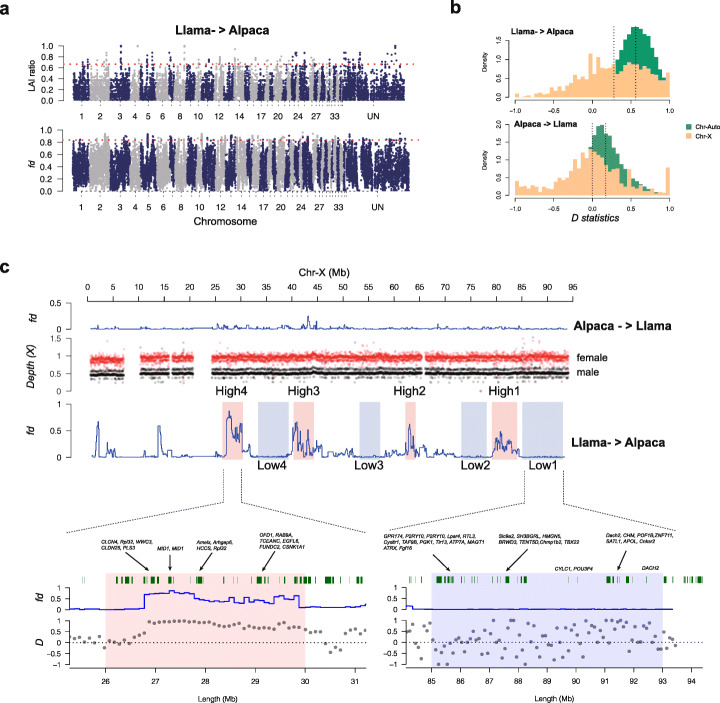
Introgression patterns along the X chromosome in comparison to the autosomes. **a** LAI (top) and *f*_*d*_ statistics (bottom) were used to detect introgressed regions from llama to alpaca. The red dotted lines indicate the 1% threshold. **b** Comparison of introgression profiles between the inferred X-chromosome and the autosomes (top: llama to alpaca; bottom: alpaca to llama). The black dotted lines indicate the median value of calculated *D* statistics. **c** Variation in introgressed signatures (high and low) and genes across the inferred X-chromosome. In the upper panel, the calculated *f*_*d*_ statistics in the X-chromosome were shown. The sequencing depths of male and female were used to identify the X-chromosome. The red and black dot points indicate the female and male sequencing depth respectively. In the lower panel, genes in the two represent regions (Low1 and High4) were zoomed in. The green rectangles represent the genes and gene symbols are shown above rectangles. The *f*_*d*_ and *D* statistics are shown below the genes

ABBA-BABA analysis produced a significantly positive *D* value for ((*alpaca*, *vicuña*), *llama*, *bactrian*) of 0.565 ± 0.006 (two-tailed *z*-test, *P* < 0.01), indicating strong genome-wide introgression between llama and alpaca, recapitulating the results of the ADMIXTURE and *Treemix* analysis. In contrast, we found a significantly negative *D* value for ((*guanaco*, *llama*), *vicuña*, *bactrian*) of − 0.185 ± 0.006 (two-tailed *z*-test, *P* < 0.01), suggesting the introgression from alpaca into llama was significantly lower (*t*-test, *P* < 0.001). The introgression proportion identified by using *f*_*d*_ analysis was ~ 38.9% from llama to alpaca and ~ 4.5% in the opposite direction (Fig. 2a, b; Additional file 1: Table S10). Local Ancestry Inference estimates of ancestry proportions for llama and alpaca were highly concordant with *f*_d_ and ADMIXTURE results, with approximately 94.5% of llama ancestry and 36% of alpaca ancestry inferred to have originally originated from guanaco/llama and 5.5% and 64% derived from vicuña/alpaca, respectively (Fig. 2a, b). We identified 290 high probability llama ancestry introgressed windows (top 1%, size = 100 kb) using LAI or *f*_d_, comprising 225 and 236 genes, respectively (Fig. 3a; Additional file 1: Table S11). There were 87 windows (comprising 101 genes) detected in common by both methods (Additional file 1: Table S11), which were investigated for GO terms (Table 1). Using the same approach, we identified 247 high alpaca ancestry regions using LAI or *f*_d_, comprising 335 and 287 genes, respectively (Additional file 1: Table S11; Figure S4) with 113 regions (192 genes) detected in common (Additional file 1: Table S12), which were also investigated for GO terms. GO analysis (Table 1) identified regions including olfactory receptor genes, putative blood pressure markers, fleece quality and color genes (bidirectional introgression), and disease resistance/susceptibility (unidirectional introgression).

**Table 1:** Genes, location, inferred or potential function, and directionality of introgression between llama and alpaca, revealed by Local Ancestry Inference and ABBA-BABA analysis (*f*_d_)

Gene	Chromosome	Function	Directionality	LAI, *f*_d_, or both
*Olfactory receptor* (*OR5*) *family*	ChrUN	Olfaction/dietary choice [14]	Llama–Alpaca	Both
*Anthrax toxin receptor 2* (*ANTXR2*)	2	Anthrax resistance/susceptibility [15]	Llama–Alpaca	Both
*Fibroblast growth factor 5* (*FGF5*)	2	Longwool/shortwool fleece [16]	Llama–Alpaca	Both
*C4orf22*	2	ANTXR2,FGF5, C4orf22 region linked to high blood pressure susceptibility [17]	Llama–Alpaca	Both
*Atrial natriuretic peptide-converting enzyme* (*CORIN*)	2	Negatively regulates Agouti, yellow coat in Hanwoo cattle [18]	Alpaca–Llama	Both
*Endothelin 3* (*EDN3*)	19	Blue-eyed, white coat phenotype in Waardenburg syndrome of human [19]	Alpaca–Llama	Both
*Olfactory receptor* (*OR2*) *family*	ChrUN	Olfaction/dietary choice [14]	Alpaca–Llama	*f*_d_
*Fibroblast growth factor 21* (*FGF21*)	11	Melanogenesis in alpaca [20]	Alpaca–Llama	Both
*Agouti signaling protein* (*ASIP*)	19	Pigmentation [21, 22]	Alpaca–Llama	*f*_d_
*Transgelin* (*TAGLN*)	33	Fiber development in Merino sheep [23]	Alpaca–Llama	*f*_d_
*GNAS complex*	19	Performance traits in cattle (imprinted) [24]	Alpaca–Llama	Both
*Cathepsin Z* (*CSTZ*)	19	Immunoprotection [25, 26]	Alpaca–Llama	Both

We examined introgression patterns along the X-chromosome in comparison to the autosomes. For segments where we could unambiguously identify contiguous X-chromosomal regions, we found relatively low *D* and *f*_d_ values (see Fig. 3b) of 0.278 ± 0.414 (s.d) and − 0.001 ± 0.435 for llama to alpaca and alpaca to llama, respectively, compared to 0.560 ± 0.203 and 0.172 ± 0.234 for the autosomes (*t*-tests for both comparisons *P* < 0.001; Fig. 3b) allowing us to infer significantly lower introgression into the X-chromosome, implying an adaptive cost of introgression between llama and alpaca as has been seen in other domestic hybrids [27] or strong ancestral selective sweeps in the region, inhibiting our ability to detect introgression because of low divergence [28]. Inferred introgression was especially variable on the X-chromosome, which, after filtering for putative Y-chromosome and pseudo-autosomal regions (PARs), featured some regions of high introgression (four regions comprising 79 genes) but also regions of almost zero introgression (four regions comprising 116 genes) (Fig. 3c). The eight regions contained genes with known deleterious or lethal X-linked effects in humans or mouse (25 genes in low introgression regions and 28 in high regions). For example, the deleterious mutations in the *PTCHD1* (patched domain containing 1) gene have been described in male patients with X-linked autism spectrum disorder (ASD) and/or intellectual disability (ID) [29, 30], which further been found that the deficiency of PTCHD1 could also induce excitatory synaptic and cognitive dysfunctions in mouse [31]. Beyond it, eight genes feature dominant inheritance, five of unknown or variable inheritance and 12 recessive for low regions and eight, nine, and 11 such genes found in highly introgressed regions (Additional file 1: Table S13). Taken together, these results suggest that selection could be limiting heterogametic introgression of deleterious variants between *Lama* and *Vicugna* in domestic crosses.

Analysis of incomplete lineage sorting (ILS) indicated a higher ILS rate for llama-guanaco-alpaca (0.275) than for llama-guanaco-vicuña (0.157), which in the absence of admixture would be expected to give similar estimates if the alpaca is descended from the vicuña. Within each species trio, the two different ILS topologies would also be expected to appear in equal proportions in the absence of admixture. However, we found a larger proportion of ((llama, alpaca), guanaco) (0.1480326) compared to ((guanaco, alpaca), llama) ILS (0.124) and a larger proportion of ((llama, vicuña), guanaco) (0.092) compared to ((guanaco, vicuña), llama) ILS (0.062). This larger proportion of ILS involving llama further suggests admixture between alpaca and llama.

Using LAI admixture tract length analysis, we obtained very similar estimates of admixture time for both domesticated species, with 121.17 generations for alpacas (113.99–130.04 95% CI; Fig. 2c) and 115.13 generations for llamas (107.4–126.02 95% CI; Fig. 2e). The generation length of llamas and alpacas has not been reported, although some widely divergent estimates are available for guanaco varying between 3 and 11 years [32, 33]. We assumed that domesticated species would have shorter generation lengths than their wild counterparts, a common feature of domestication and selective breeding and loss of seasonality [27, 34]. Given the range of guanaco estimates available, the implications of the LAI results for 3, 4, 5, and 6-year generation lengths (the mean estimate for guanaco being 7 years) are events occurring at 345, 461, 575, and 691 years ago, respectively, for llama and 364, 485, 606, and 727 years ago for alpaca. The most plausible explanation for a contemporaneous single-pulse admixture event occurring for both domestic species in this geographic region within this timeframe is the arrival of the Spanish Conquistadors (starting 1529). If this event explains the admixture tracts within the genomes of the domestic species, we can infer a generation length for both species at approximately 4 years.

### Demographic history

PSMC (Pairwise Sequential Markovian Coalescent) showed signatures of a strong demographic expansion from one million years before present (BP) for vicuña and alpaca, while guanaco and llama showed an expansion only after c 400,000 years, having undergone a decline immediately prior to this expansion (Additional file 1: Figure S5). All species showed evidence of a subsequent strong decline from 100,000–200,000 years BP to 25,000 years BP, overlapping with the last two major glacial periods. The alpaca genome showed evidence of a stronger and longer expansion than the vicuña; however, an even more extreme expansion for alpaca (to effective size *N*_e_ < 50,000) was also detected in a previous study [8], but this study did not remove admixture tracts prior to analysis, potentially inflating the *N*_e_ estimates of the simulation [28]. To detect more recent *N*_e_ changes, we used MSMC (Multiple Sequence Markovian Coalescent), taking advantage of a sample of 6 (alpaca and llama) or 8 (vicugna and guanaco) resequenced genomes in this study and found concordant evidence for declines in all four species from 100,000 to 10,000 years BP. Higher *N*_e_ estimates could be inferred for *V*. *v*. *vicugna* compared to *V*. *v*. *mensalis*, consistent with their larger geographic range, but no such pattern was found for *L*. *g*. *guanicoe* compared to *L*. *g*. *cacsilensis*, with the variance among samples being larger than for *Vicugna* (Additional file 1: Figure S6). SNeP (SNP linkage disequilibrium-based *N*_e_ estimation), the resolution of which enables *N*_e_ trends and their variance to be inferred over the last 200 generations [35, 36], showed a consistent signature of further population decline (Fig. 2d), with the largest decline detected in alpaca, both when only the most highly significant vicuña ancestry portions of the alpaca genome were included (43% of the genome retained) and when only the most highly significant guanaco ancestry portions of the alpaca genome were removed (64% retained). Estimated *N*_e_ values ranged from 2500 (llama) to 3500 (vicuña-ancestry alpaca), closely resembling those values from 1000 years BP in both species’ MSMC simulations. NeS analysis [36] identified considerable variation in the *N*_e_ decline slope, especially in the recent past (50 generations), but interestingly identified an increase in the rate of decline for both domestic species at approximately 110 generations ago, which coincides closely with the LAI tract-based estimate of time of admixture (Fig. 2f).

### Selection signatures for domestication and introgressed segments

Combining *F*_ST_ outlier and extended haplotype homozygosity (XP-EHH) analysis, we analyzed signatures of domestication by directly comparing wild ancestor and domestic descendant pairs (vicuña versus alpaca and guanaco versus llama). For this analysis, we did not use the genome data for which admixture tracts had been removed to enable us to compare introgression “landmark” signatures with domestication signatures in the same analysis. For vicuña versus alpaca, mean *F*_ST_ across the genome was 0.086 ± 0.153. For domestication signals in alpaca, we identified 92 and 128 regions, comprising 647 and 817 genes, using *F*_ST_ and XP-EHH, respectively (Additional file 1: Table S14–15; Figure S7). However, only four regions comprising 35 genes showed overlapping peaks between the two methods (Additional file 1: Table S16). Although overlap between the two methods was limited, aside from the expected introgressed segments inferred to have originated from llama, putative domestication regions were linked to fecundity/development of secondary sexual characters, coat color, hypoxia, meat quality, and olfaction [37] (Table 2). For guanaco versus llama, the mean *F*_ST_ across the genome was 0.064 ± 0.128. For domestication signals, we identified 141 and 126 regions, comprising 999 and 800 genes, using *F*_ST_ and XP-EHH, respectively (Additional file 1: Table S17–18; Figure S8). Fifteen regions comprising 79 genes showed overlapping peaks between the two methods (Additional file 1: Table S19). Although overlap between the two methods was again limited, aside from the expected introgressed segments inferred to have originated from alpaca, putative domestication regions were linked to blood pressure control, spatial memory and social behavior, skin and coat color, domestic performance traits, and dietary restriction and appetite [34] (Table 2).

**Table 2:** Genes, location, inferred or potential function, and origin of domestication signatures in alpaca and llama, revealed by *F*_ST_ outlier and extended haplotype homozygosity (XP-EHH) analysis

Gene	Chromosome	Function	Origin	Domestic species	*F*_ST_, XP-EHH, or both
*Anthrax toxin receptor 2*	2	Anthrax resistance/susceptibility [15]	Introgressed from llama	Alpaca	Both
*Fibroblast growth factor 5*	2	Longwool/shortwool fleece [16]	Introgressed from llama	Alpaca	Both
*C4orf22*	2	ANTXR2, FGF5, C4orf22 region linked to high blood pressure susceptibility [17]	Llama–Alpaca	Alpaca	Both
*Luteinizing hormone receptor*	ChrUN	Fecundity and development of male secondary sexual characters [38]	Domestication	Alpaca	Both
*Agouti signaling protein*	19	Pigmentation [21, 22]	Domestication	Alpaca	Both
*Hypoxia upregulated 1*	33	Cytoprotection under hypoxic conditions [39]	Domestication	Alpaca	XP-EHH
*Myomesin 1*	24	Meat quality in cattle, sheep, and pigs [40–42]	Domestication	Alpaca	*F*_ST_
*Melanocortin 5 receptor*	23	Meat quality [43]	Domestication	Alpaca	Both
*Melanocortin 2* (*adrenocorticotropic*) *receptor*	24	Circadial rhythm [44]	Domestication	Alpaca	*F*_ST_
*Melanocortin 1 receptor*	24	Light fiber color [45]	Domestication	Alpaca	*F*_ST_
*Olfactory receptor* (*OR5*) *family*	ChrUN	Olfaction/dietary choice [14]	Introgression from llama	Alpaca	*F*_ST_
*Olfactory receptor* (*OR2*) *family*	ChrUN	Olfaction/dietary choice [14]	Domestication	Alpaca	*F*_ST_
*G protein-coupled estrogen receptor 1*	ChrUN	Spatial memory and social behavior [46]	Domestication	Llama	Both
*Premelanosome protein*	ChrUN	Skin and coat color in wild and domestic mammals [47]	Domestication	Llama	*F*_ST_
*Anthrax toxin receptor*	2	Anthrax resistance/susceptibility [15]	Domestication	Llama	Both
*Fibroblast growth factor 5*	2	Longwool/shortwool fleece [16]	Domestication	Llama	Both
*Endothelin 3*	19	Blue-eyed, white coat phenotype in Waardenburg syndrome of human [19]	Introgression from alpaca	Llama	*F*_ST_
*Agouti signaling protein*	19	Pigmentation [21, 22]	Introgression from alpaca	Llama	XP-EHH
*GNAS complex*	19	Performance traits in cattle (imprinted) [24]	Introgression from alpaca	Llama	*F*_ST_
*Agouti-related neuropeptide*	9	Dietary restriction and appetite in cattle [37]	Domestication	Llama	*F*_ST_

### Evidence for adaptive introgression

**Fig. 4:**
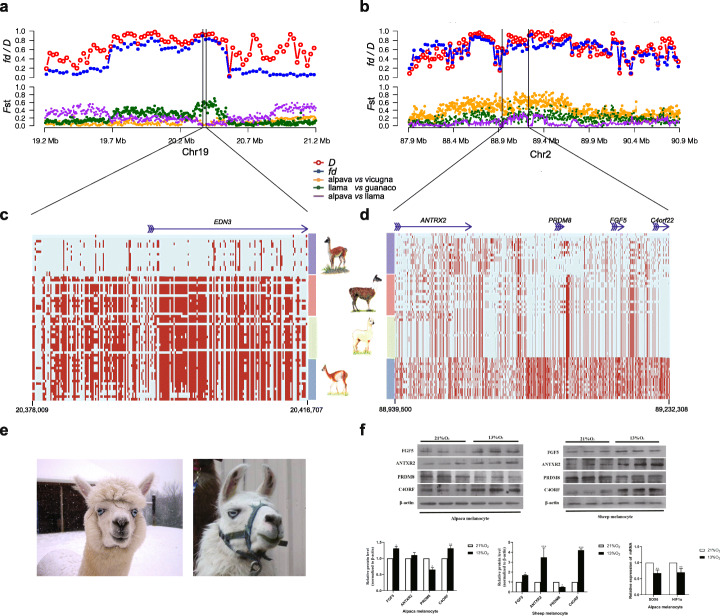
Evidence for adaptive introgression and functional analysis. **a***D* and *f*_d_ statistics for ((*alpaca*, *vicuña*), *llama*, *Bactrian camel*) and the respective *F*_ST_ values for alpaca versus vicuña, llama versus guanaco, and alpaca versus llama. **b***D* and *f*_d_ statistics for ((*llama*, *guanaco*), *vicuña*, *Bactrian camel*) and the respective *F*_ST_ values for alpaca versus vicuña, llama versus guanaco, and alpaca versus llama. **c***EDN3* introgression profile, inferred from alpaca to llama and with origin in vicuña, controlling the blue-eyed, white fleece phenotype now found in both species. The rectangles with red color indicate the same allele with the reference genome, while the light blue rectangles indicate the alternative allele. **d***ANTRX2*, *PRDM8*, *FGF5*, and *C4orf22* introgression profile, inferred from llama to alpaca and with origin in guanaco, potentially controlling anthrax susceptibility, longwool/shortwool fleece, and blood pressure, now found in both species. **e** The typical phenotype of blue-eyed alpaca and llama. **f** Upper: Western Blot result of FGF5, ANTXR2, PRDM8, and C4orf22 in alpaca and sheep melanocytes. Lower: (left and middle) protein expression levels by densitometric analysis of FGF5, ANTXR2, PRDM8, and C4orf22 in alpaca and sheep melanocytes. Protein abundance was normalized relative to the abundance of β-actin. Bars represent mean values ± standard error (*N* = 3). *P < 0.05, **P < 0.01, ***P < 0.001. The experiment was repeated for three times. Lower: (right) qPCR for *HIF1α* and *SOX6*. Resulting mRNA abundance for *HIF1α* and *SOX6* was normalized using *18S rRNA*. All reactions were performed 4 times. mRNA abundance of *HIF1α* and *SOX6* was significantly decreased at 13% O_2_ compared to 21% (*P < 0.05 and **P < 0.01, respectively)

We then attempted to combine an understanding of both introgression and selection identified via the co-detected segments in our domestication analysis by focusing on four introgressed regions (*ANTRX2/PRDM8/FGF5/C4orf22* and *OR5* family for llama to alpaca, *EDN3* and *OR2* family for alpaca to llama). We examined phased haplotypes along the inferred introgressed regions to confirm both their ancestry from vicuña or guanaco and the variance in *F*_ST_ between alpaca and llama to establish which segments showed the strongest signatures of selection (Fig. 4a–d).

For the *ANTRX2*/*PRDM8/FGF5*/*C4orf22*, the guanaco origin of the domestic haplotype is clearly evident, strongly supporting the inference that it was introgressed from the llama into the alpaca (Fig. 4b). Inspection of the *F*_ST_ distribution along the region shows a highly heterogeneous pattern, with almost no genetic differentiation between llama and alpaca coincident with 3′ end of the *C4orf22* gene and downstream (Fig. 4b). Further, SNP variation is, represented by genetic diversity (*θ*_*π*_), as expected, highest in guanaco, followed by llama and with alpaca nearly devoid of SNPs in this region (*θ*_*π*_-guanaco = 0.17; *θ*_*π*_-llama = 0.13; *θ*_*π*_-alpaca = 0.02), suggesting serial bottlenecks and/or selection occurring first at the domestication stage (guanaco–llama, where *F*_ST_ and XP-EHH scores were 0.106 and 0.269, respectively) and second at the introgression stage (llama–alpaca, where LAI and *f*_*d*_ scores were 0.912 and 0.867, respectively). In contrast, for the *EDN3* gene, while the alpaca origin of the domestic haplotype is clear (Fig. 4c), supporting its vicuña origin, there is no evidence for a loss of diversity between vicuña and alpaca (*EDN3* does not feature in either the *F*_ST_ or XP-EHH candidate list; Fig. 4a) or at the stage of admixture (alpaca–llama, where LAI and *f*_*d*_ scores were 0.400 and 0.664, respectively), suggestive of recent artificial selection for this phenotype in the domestic forms (white fleece, blue eyes [48]; Fig. 4e). In both alpacas and llamas, white individuals with blue eyes are often deaf, the phenotype of which is known to be regulated by *KIT* [35]. However, in the phenotypically similar Waardenburg syndrome in humans, these phenotypes are associated with *EDN3*, *SOX10*, and *EDNRB* [19]. While the linkage of these phenotypes to *EDN3* in SAC was previously unsuspected, studies have suggested *EDN3* is linked to white pelage in wild cats [49] and the co-occurrence of deafness (congenital deafness) and white pigmentation is a common observation in domestic animals [50].

We also found that the number of SAC functional olfactory receptor (*OR*) genes was substantially lower than for other domestic livestock (approximately 50% of cattle and horse, at 566–602; Additional file 1: Table S20). For *OR5*, while the wild origin of the haplotype (especially 5′ end) in the guanaco was clear for both domestic forms, a mosaic pattern of similarity is evident across the family, with no consistent changes in *F*_ST_ being evident (Additional file 1: Figure S9 left). For *OR2*, a vicuña origin is clear, but again, no consistent patterns are discernible across the region (Additional file 1: Figure S9 right).

Given the extensive admixture detected between llama and alpaca, a wider investigation of introgressed segments was carried out (*HoxD* cluster, *EVX2*, *OLA1*, and a 1.5 Mb region on Chr10 from llama to alpaca, including *C2CD3*, *RAB6C*, *ATG16L2*, and *RNF121*; *CORIN*, *FGF21*, *GNAS*, and *TAGLN* from alpaca to llama; see Additional file 1: Figure S10–12). For *HoxD* (*HoxD3*, *HoxD4*, *HoxD10*, *HoxD12*, and *HoxD13*), *EVX2* and *OLA1*, we found clear evidence of introgression from llama to alpaca (Additional file 1: Figure S10). Mutations in *HoxD*, *EVX2*, and *OLA1* genes are known to result in abnormal skeleton development [51, 52] and introgression of *HoxD*, *EVX2*, and *OLA1* from llama to alpaca could therefore influence the development of the alpaca’s larger body size compared with vicuña, as evidenced, for example, by obvious shoulder height differences [53] (Additional file 1: Figure S10–12).

### Functional analysis

We found that the coding sequences (CDS) for *FGF5* in male and female Suri and Huacaya alpaca skins were identical except for two *T* - *G* substitutions at sites 50 and 346 (Additional file 1: Figure S13a). To establish whether *FGF5* was expressed differentially in the skin of Suri (longwool) and Huacaya (shortwool) alpaca, quantitative real-time PCR (qPCR) analysis was carried out and showed that *FGF5* mRNA showed significantly elevated expression in Suri alpaca, both for males and females (two-way ANOVA *P* < 0.001) (Additional file 1: Figure S13b), a result replicated in concomitant Western Blot assays (two-way ANOVA *P* < 0.001) (Additional file 1: Figure S13c, d). Expression profiles for the 20 single-copy genes identified during the introgression and selection signature analysis were compared within alpaca and among the three de novo sequenced specimens. We found very high expression levels for transgelin (7x higher compared to all other genes) in alpaca and llama skin (expressed at a similar level to alpaca, although in blood, while being effectively absent from the wild species). This gene was identified as being introgressed from alpaca to llama has been linked to skin and wool follicle development in merino fine-wool sheep [23]. To investigate whether *FGF5*, *ANTXR2*, *PRDM8*, *C4orf22* haplotype could be related to hypoxic response, as some evidence has already been found in other mammals [54, 55], their expression was investigated in alpaca and sheep melanocytes incubated under normoxic (21% O_2_) and hypoxic (13% O_2_) conditions. While *FGF5*, *ANTXR2*, and *C4orf22* were more highly expressed in hypoxic conditions, surprisingly *PRDM8* at 13% O_2_ was significantly less expressed than at 21% O_2_ in both cell lines. Similarly, mRNA expression of Sry-related HMG-box-containing transcription factor 6 (*SOX6*) and its downstream regulated hypoxia-related gene *HIF1*α [56] were downregulated at 13% compared to those at 21% O_2_ (Fig. 4f).

## Discussion

These first genome-scale results for wild and domestic South American camelids convincingly support the hypothesis that the alpaca was domesticated from the vicuña and that the llama was domesticated from the guanaco. Figure 1b shows that despite the wide spread of alpaca lineages in the *Vicugna* clade, they group together with 100% bootstrap support, despite the > 30% introgression from llama that was detected. When we recalculated the phylogeny with the introgression segments removed from the alpaca genome (Additional file 1: Figure S14), all individuals grouped together and with *V*. *v*. *mensalis* with 100% bootstrap support, pointing towards this subspecies as the origin of the alpaca. Interestingly, however, all alpaca grouped within a separate clade from *mensalis*, suggesting that the population of *V*. *v*. *mensalis* that was originally domesticated is not covered by our *mensalis* samples. It remains possible, however, that residual introgression segments which we were unable to reliably detect using LAI have influenced the shape of the phylogeny and could underlie the separation of alpaca from *mensalis* (Fig. 1a). Figure 1a also reveals that *Lama guanicoe cacsilensis* is most similar to *L*. *glama*, which is recapitulated in Fig. 1c, where the two northern *cacsilensis* samples group with llama (*k* = 4). However, while we consider the phylogenetic origins of these domestic species to be resolved, we did not attempt to address how many times each species was domesticated nor the likely precise origins of domestication, which will require greater sampling of extant populations, especially in Peru, combined with an ancient DNA approach.

The level of admixture detected in the genomes of all alpacas sequenced is among the highest detected in a domesticated animal to date: all estimates exceeded 30%. Figure 1a shows the admixture to be pervasive and remarkably consistent across individuals, implying that admixture happened in a consistent manner. Although a much smaller proportion of the llama genome is admixed (c 5%), hybridization has clearly been bidirectional. All methods gave similar results but the LAI analysis of admixture tract lengths (Fig. 2c, e) allowed us to conclude that a single major event occurred contemporaneously for both species and is relatively recent, occurring just over 100 generations ago, consistent with the low variance in ancestry components detected [57]. While a precise estimate of the llama and alpaca generation length is not available, estimates using data from wild SACs would place the event at 400–600 years ago. The one major event that is known to have occurred during this period was the Spanish conquest. Within a century of the conquest of Cuzco in 1532, administrative and taxation documents record the “*precipitous decline and virtual disappearance of previously extensive herds throughout the Andes*”, and while early Spanish writers noted the importance of SACs in the Andean pastoral system, they failed to distinguish between the llama and alpaca in their writings [3]. At a time of mass human and SAC mortality and replacement, under a subjugate system that failed to acknowledge the distinctiveness of the two domestic species, and with indigenous records and knowhow in breeding being passed down by oral tradition only, it seems likely that in the years after the conquest, established breeding practices broke down, including the maintenance of species boundaries and fleece types.

The demographic history of SACs since the divergence of Lama and Vicugna c 2.5 M years before present shows evidence for a strong expansion, albeit earlier for the vicuña, with the expansion of the genus *Lama* coinciding with the Mindel-Riss/Holstein interglacial period followed by a decline over the last 200,000 years, which has continued largely to the present day (Additional file 1: Figure S5 and S6). The difference between the two genera may reflect their altitudinal partitioning and reliance of the guanaco on multiple Andean habitats, which would have opened up during this interglacial period, potentially allowing the population to expand. These results are interesting in the context of recent studies on the genetics of the wild species [57, 58] that have shown both a signature of demographic expansion in vicuña and guanaco and a recent demographic decline in vicuña, and our results support the latter conclusion [52]. However, the multi-locus microsatellite data used in these studies did detect a strong bottleneck 2000–3000 years ago, which coincides the period of strongest decline in our MSMC plots (Additional file 1: Figure S6). We were most interested in understanding fine-scale changes in effective population size in the last 1000 years for the domestic species and SNeP and NeS analysis both confirmed a continuing decline from 200 generations ago to the present day (Fig. 2d), to a current effective size of less than 1000. Interestingly, a coincident acceleration in this decline was detected approximately 110 generations ago using NeS (Fig. 2f), very close to the date estimated for the admixture pulse and in line with the recorded decline following the Spanish conquest [3]. So, taken together, the admixture tract and SNeP analysis point to a single event in both species occurring 100–110 generations ago. The most plausible explanation for this decline in population size and the onset of substantial bidirectional introgression in these domestic species is the chaos and loss of traditional management practices in Andean communities immediately after the conquest.

The phenotypic consequences of the admixture pulse have never been studied, despite evidence of its importance. Annotation of the introgressed regions (Table 1) revealed several large haplotypes containing a number of genes that could be both separately or coincidentally introgressed. These include a haplotype including the *Anthrax toxin receptor 2* gene (*ANTXR2*) and *fibroblast growth factor 5* (*FGF5* - introgressed from llama to alpaca). This haplotype has been implicated in fiber characteristics in alpaca and blood pressure control in humans (Table 1) and *Bacillus anthracis* is also known to be prevalent in the soil at elevations below 4000 m ASL in the Andes, but to be absent above this altitude [59]. Other genes detected included *olfactory receptor families 2* and 5 (*OR2* and *OR5*), genes linked to dietary choice [14] and coat color. The extent to which this introgression was deliberate or occurred accidentally is not known and merits further study, especially using historical records.

Signatures of domestication, identified by comparing the genomes of the domestic species with their wild ancestors (Table 2), as expected highlighted genes involved in coat color variation, sexual and reproductive traits, and dietary choice as well as a number of genes implicated in production traits (both meat and milk) in other livestock species. Interestingly, the alpaca whose wild ancestor, vicuña, lives at high altitudes, showed selection signatures for the hypoxia upregulated gene [39], while llama (whose wild ancestor, the guanaco, lives in small social groups and undertakes seasonal altitudinal migration) showed a signature for a G-coupled estrogen receptor previously linked spatial memory and social behavior [46]. As expected, the inferred introgressed segments described above were also identified as domestication signatures, implying that some are operating in parallel in the two domestic forms.

To further investigate the adaptive or selective value of the most significant introgressed segments in this study, we evaluated their sequence diversity in more detail. First, while admixture has been bidirectional, the generally lower levels of introgression in the X-chromosome suggest a potential barrier to hybridization which might have further impacted on effective population size trajectory after the conquest. This phenomenon has been observed in comparisons between taurine and indicine cattles [60], but the ancestors of those two bovine forms are generally thought to have been subspecies, not species from separate genera, for which the cost of introgression is expected to be considerably higher.

Analysis of the sequences that is syntenic to the human HSA4q21 locus comprising *ANTRX2*/*PRDM8/FGF5*/*C4orf22* (llama–alpaca), *EDN3* (alpaca–llama), and the *OR5* and *OR2* gene families revealed contrasting patterns of genetic erosion, ranging from the expected serial loss of SNP diversity from wild ancestor to domesticated descendant and then into the introgressed species (see *ANTRX2*; Fig. 4d), to very little difference detected in SNP diversity across all three species (*EDN3*), to a complex mosaic of SNP diversity across the *OR* families though we cannot rule out the possibility that some *OR* SNPs may be artifacts due to misaligned sequences. Our results thus suggest multiple evolutionary processes at play and the need to examine their evolution on a case-by-case basis. The evolutionary distinctiveness of the wild ancestors of these two domestic forms means that detecting introgression events is feasible; however, understanding their functional significance is less straightforward than, for example, can be carried out within and among livestock breeds within the same species which have an extensive history of QTL investigation, e.g., in cattle [61]. Intriguingly, the HSA4q21 locus has been linked using GWAS, to blood pressure homeostasis in Asians and Europeans. A mouse model has been used to identify the causative gene in humans, which was found to be *ANTXR2*, whereas blood pressure did not differ between *FGF5* knockout and wild-type mice [17, 62]. Systematic studies have identified a positive link between altitude (and thus hypoxia) with the frequency of hypertension in humans [63] and *PRDM8* belongs to a family of histone methyltransferases that are known as negative regulators of transcription. Thus, in contrast to the other upregulated genes in the “HSA4q21” region, the downregulation of *PRDM8* at high altitude may confer cytoprotection by upregulating the expression of other genes in a manner analogous to the hypoxia-inducible factor gene (e.g., *HIF2*α) [64]. Our qPCR results showed that mRNA expression levels for the genes *SOX6* and *HIF1α* are similarly downregulated. *SOX6* is one of the targets of *PRDM8* in the retina and its expression is controlled by *PRDM8* [65]. The response of *HIF1α* may therefore be indirect via the expression of the transcription factors SOX6 (and/or SOX5 and SOX9), depending on levels of atmospheric oxygen availability [56]. Under hypoxia, mitochondrial O_2_ consumption is inhibited or intracellular Fe and prolyl hydroxylase domain (PHD) activity increases, which decreases the expression of *HIF1α* [56]. Therefore, *FGF5*, *ANTXR2*, and *C4orf22* may play a key role in regulating hypoxia stress, while *PRDM8* is a novel gene associated with hypoxic adaptation, the expression of which may be mirrored by decreased expression of *SOX6* and *HIF1α*. *FGF5* was originally reported as a key human oncogene [66], associated with a number of cancers [67], angiogenesis in human aortic endothelial cells [68], and trichomegaly in humans [69]. *C4orf22* is expressed in human neural progenitor cells expressing MEF2CA [70]. Functional analysis of the *FGF5* gene in Suri and Huacaya alpacas has previously shown its potential importance in regulating fleece length [71], and preliminary analysis of expression profiles in domestic SACs has shown the potential importance of a novel QTL for fine fiber production in merino sheep [23]. Further studies should focus on investigating these genes and the QTL they underpin.

## Conclusions

That domesticated South American camelids have undergone genetic erosion is clear from the decline in both species’ effective sizes over the recent 1000 years, and it is plausible that the Spanish conquest and its aftermath have contributed to this substantially. However, it is also the case that some of the introgressed segments we have identified have potentially allowed new avenues for selection, for example, for coat color, fiber characteristics, and adaptation to high altitude and harsh environment. Therefore, introgression, which has negative connotations in species management or conservation, but which genome data are showing to be far more pervasive than previously appreciated, should be afforded a more nuanced interpretation in domestic species and does not necessarily contribute to genetic erosion.

## Methods

### Sampling and DNA sequencing

Blood and tissue samples were obtained from eight wild guanacos, vicuñas, and domestic llamas and from seven alpacas. Sampling of guanacos and vicuñas represents the entire range of geographic distribution of each species (Additional file 1: Table S8). One adult male guanaco from Putre (Chile) and adult female vicuña from Lauca National Park (Chile) were selected for de novo assembly, representing the northern subspecies of wild South American camelids, *Lama guanicoe cacsilensis* and *Vicugna vicugna mensalis*, respectively. Llama tissues were obtained from Putre and alpaca from Temuco (Chile; Additional file 1: Table S8). Genomic DNA was extracted using the Puregene Tissue Core Kit A (Qiagen). See Additional Material S3 for further information and permit details.

### De novo assembly, alignment, and annotation

De novo assembly for *L*. *guanicoe*, *V*. *vicugna*, and *L*. *glama* used Illumina HiSeq 2000 and HiSeq 2500 platforms (see Additional file 1: Supplementary text for full description). Paired-end libraries with insert sizes of approximately 170, 500, and 800 bp, and mate pair libraries with insert sizes of 2, 5, 10, 20, and 40 kb were constructed. We generated 335, 285, and 262 Gb of high-quality data for each species, respectively (Additional file 1: Table S1). SOAPdenovo (v2.04) [9] was used to assemble the genomes (Additional file 1: Table S2). Annotation used a standard combination of ab initio gene prediction, homolog searching, and EST/unigene-based prediction. To aid gene prediction and to enable transcript data to be analyzed at the species level, we generated RNA-seq data for blood samples from the vicuña, llama, and guanaco used for de novo sequencing and from skin samples for a set of 17 alpaca samples from China (Additional file 1: Table S8). Expression patterns were compared within and among species for specific genes by aligning to the guanaco reference gene set using Bowtie2 [72] and then RSEM [73] was used to estimate the *Fragments Per Kilobase of transcript per Million mapped reads* (FPKM). For the de novo gene predictions, AUGUSTUS (v2.5.5) [74] and GENESCAN (v1.0) [75] were used to identify candidate protein-encoding genes in the masked genome with self-trained model parameters. For homology-based predictions, we used the genomes of *B*. *taurus*, *C*. *bactrianus*, *C*. *dromedarius*, *E*. *caballus*, *H*. *sapiens*, *M*. *musculus*, and *V*. *pacos*. These were mapped to a repeat masked assembly using TBLASTN2.2.23 [76] with an *E*-value threshold of 1E−5. Subsequently, homologous genome sequences were aligned against the matching proteins to define gene models using GENEWISE (v2.2.0) [77]. For the EST-based method, unigenes were mapped to the genome using BLAT (identity ≥ 90%, coverage ≥ 90%), and overlaps among the spliced alignments were filtered and linked using PASA [78]. Gene models supported by the three methods were integrated to yield the final gene-set using GLEAN (Additional file 1: Table S7a–c). To assign functions to the gene models, we used the SwissProt and TrEMBL protein databases (Uniprot release 2011-01) using BLASTP with an *E*-value threshold of ≤ 10^−5^. Domain-based comparisons were performed and searched to identify conserved domains/families. Functional annotation was carried out using Blast2GO [79]. Metabolic pathway annotations were performed by sequence comparisons with KEGG proteins (Release 76) using BLASTP.

### Resequencing of individuals of four camelid species

Resequencing produced 35–49 Gb of raw data per individual. We removed reads with ≥ 10% unidentified nucleotides, > 10 nt aligned to the adaptor, with ≤ 10% mismatches allowed, with > 50% bases having phred quality < 5 and putative PCR duplicates generated during library construction. Details of the throughput and read lengths are summarized in Additional file 1: Table S9.

### Chromosome level reference genome and SNP calling

To obtain the chromosome level reference genome, we aligned our assembled guanaco scaffolds against the VicPac3.1 [80] using LAST [81] and get the chromosome information for each scaffold. Due to the lack of ordering information for scaffolds within each chromosome of their assembly, we joined our scaffolds together after sorted them by length within each VicPac3.1 chromosome. For unmapped scaffolds, we connected them together and classified them as “ChrUN”. In total, ~ 2.1 Gb of assembled scaffolds in our study could be mapped to chromosomes in VicPac3.1 and ~ 499.7 Mb were classified as “ChrUN,” the same level as those reported by VicPac3.1. We also mapped the reads of two selected individuals (one male and one female) onto our assembled scaffolds and identified X fragments based on the sequence depth ratio (male/female ≥ 0.5). We next connected the identified X fragments together and used the assembled sequence as our X chromosome sequence.

High-quality reads were then aligned to the above reference genome using Burrows-Wheeler Alignment MEM (BWA-MEM) [82], and alignment statistics were obtained using SAMtools (v1.2) [83]; reads were filtered with a map quality lower than 5 and a mismatch longer than 5 bp. The reads of female individuals from our study were mapped onto the X chromosome respectively. Base Quality Score recalibration and indel realignment were performed prior to variant calling, which was carried out using Picard (http://broadinstitute.github.io/picard). BAM files were adjusted using the Genome Analysis Toolkit (GATK, v3.4) [84] with the HaplotypeCaller method, using a hidden Markov model likelihood function.

### Phylogenetic, admixture, and lineage sorting analyses

To examine the evolutionary relationships among SACs across the genome, a sliding window with length and step of 250 bp was used to select SNPs, mapped to the guanaco reference genome. One SNP was selected per window, resulting in 5,901,447 positions being analyzed. A neighbor-joining tree (sample location in Fig. 1a) was then constructed using uncorrected *P*-distances in TreeBestv1.9.2 [85] with 100 bootstraps (Fig. 1a). We also performed population clustering analyses using the software package ADMIXTURE [86]. Specifically, we used PLINK [87] to extract variant sites that were not in significant linkage disequilibrium (LD) with any other site within a 100 kb window. We then ran ADMIXTURE with *k* = 2, 3, and 4 (Fig. 1c). We explored the detected admixture further in a phylogenetic context by using *Treemix* [88], which allows the evaluation of the effects of genetic drift and gene flow on a maximum likelihood phylogeny. This software models the relationship among the sample populations with their ancestral population using genome-wide allele frequency data and a Gaussian approximation of genetic drift. The *f* index representing the fraction of the variance in the sample covariance matrix ($\hat{w}$) accounted for by the model covariance matrix (*w*) was used to identify the information contribution of each migration vector added to the tree. Up to 20 possible migration vertices were computed.

We more explored introgression among SAC species in more detail at the genomic level, first by using an ABBA/BABA approach [89] (see Additional file 1: Supplementary Text). The *Bactrian* camel was used as an outgroup, and all possible four-taxon topologies of alpaca, vicuña, llama, guanaco were analyzed. We calculated both Patterson’s *D* [90, 91] and modified *f* statistics (*f*_d_) [89]. The frequency of the derived allele on each locus in each species was used instead of binary counts of fixed ABBA and BABA. A significant positive *D* value signifies an excess of shared derived alleles between taxa while a significant negative value indicates the presence of gene flow. A block size of 30 Mb was selected to calculate standard errors of *D* by bootstrapping. To identify introgressed segments from llama to alpaca, a 100-kb window size was chosen for calculating the *f*_d_ across the whole genome (Fig. 2a, b). We separately examined *D* and *f*_d_ across the constructed X-chromosome to detect whether the pattern of admixture differed from the autosomes.

Finally, we used Local Ancestry Inference and estimates of LA tract length to simultaneously estimate admixture times and LA using a Hidden Markov Model [92]. In the absence of a recombination map and chromosome-scale assembly for SACs, we estimated a genome-wide recombination rate by assuming one cross-over per generation per chromosome, obtaining 1.82e−8 crossovers/base pair; owing to the genetic divergence between guanaco and vicuña, we could extract sites where allele frequencies differed by at least 50% between the ancestral populations. We required that 90% of samples had a read depth of 10 or more at each site and that successive ancestry informative sites be separated by at least 10 kb, in order to reduce the impacts of LD on admixture time estimation, although we note that LD has a minimal impact on these estimates for genetically divergent ancestral populations such as guanaco and vicuña. For all llama and alpaca samples, we imported the filtered read counts from the *VCF* files as the primary dataset for Local Ancestry Inference. Annotations of inferred introgressed regions were investigated for putative functions of relevance in SACs, other livestock species, and model mammals, including humans. For each domesticated population, we fitted a single pulse admixture model to genome-wide variation data, using 1000 block bootstraps of size 5000 ancestry informative sites. From these analyses, we simultaneously estimated local ancestry proportions and time since admixture, assuming a single pulse, for llama and alpaca (Fig. 2a, b).

Incomplete lineage sorting among phylogenetically related species, such as the vicuña and guanaco, can potentially obscure signatures of introgression between them (or here, between their domestic descendants, alpaca and llama). We compared the estimates of admixture generated above with estimates using ILS analysis with CoalHMM [93], a maximum likelihood approach that allows the simultaneous estimate of ILS, divergence time, and ancestral effective population size. Only alignment blocks with the *L*. *glama* scaffold were included, to enrich for syntenic regions and constrain the number of scaffolds to be analyzed—this resulted in 556 syntenic blocks being included. We called hidden states along the genomic alignment with highest posterior probability and computed the proportion of reference genome positions in each state in windows of 10 kb, 100 kb, and 1 Mb. We only considered windows where more than 30% was covered by the analyzed alignment.

### Demographic history simulation

We used the sequential Markovian coalescent to evaluate the demographic history of the four SACs, starting with the Pairwise method (PSMC) [94], which reconstructs long-term changes in effective population size over the evolutionary history of the species (here approximately 2 M to 10 K years ago), assuming a generation time of 5 years and a mutation rate of 7E−9 per site per generation which was used in the reference [8]. Because admixture is known to bias PSMC outputs in favor of detecting demographic declines [95], we removed the admixture tracts identified using LAI (see the “Results” section) before carrying out the simulations. Regions with LAI probability of 0.8 or greater of originating from introgression were removed and the flanking region reconnected where three or more contiguous sites of non-introgressed ancestry. De-introgressed sequences were then realigned for downstream analysis. We aligned short reads to their respective reference genomes using BWA-MEM (v0.5.9) with the setting aln –I –o 0 –l 31 –k 2 –t 4 and aligned the SNPs using SAMtools (v0.1.17), dividing them into non-overlapping 100 bp bins, scored as heterozygous if there was a heterozygote in the bin, or as homozygous otherwise. The resultant bin sequences were used as the input for PSMC. Bootstrapping was performed by randomly resampling 100 sequences from the original batch and the reconstructed population history was plotted using gnuplot4.4. Next, we used multi-sample sequential Markovian coalescent (MSMC) [96] to investigate more recent changes (up to 2 K years ago). SNP *VCF* files were obtained using GATK and all sites were phased using SHAPEIT2 [97]; mask files were constructed using *BAM* files, created during the SNP calling step. Two models, “*fixedRecombination*” and “*skipAmbiguous*,” were used to infer population size changes and divergences. For bootstrapping, pairs of samples within-population and cross-population were randomly selected to infer their population size and coalescence rates. MSMC was run with the default parameters and fixed recombination rate for 20 M iterations. Results were plotted in *R* using *ggplot2* and scaled with a generation time of 5 years and the mutation of 0.7E−8 per site per year. To better understand very recent trends in effective population size for all species (i.e., within the last c1,000 years), we implemented the linkage disequilibrium-based method of SNeP v1.11 [35] that uses high density genome-wide data and can take account of variation in sample size, mutation, phasing, and recombination rate. Using this approach, we further documented short-term changes in *N*e by implementing NeS [36], which records the change in slope of the inferred *N*e trend obtained from SNeP, a more detailed picture of population changes over the period studied. A constant rate of change is shown as a flat line proximal to 0 in the *Y*-axis, whereas deviations above and below 0 represent relative increases and reductions in *N*e, respectively. Alpaca genomes were analyzed after removal of regions of high guanaco ancestry, retaining 64% of the original genome, and a more stringent approach was taken of only retaining the regions with high vicuña ancestry, retaining 43% of the original genome.

### Selection signatures of environmental adaptation, domestication, and introgression

We took a population genomics approach to identify selection during SAC domestication, by comparing the (ancestral) *V*. *v*. *mensalis* samples with alpaca and the (ancestral) *L*. *g*. *cacsilensis* samples with llama, using the whole-genome datasets where introgressed tracts were removed. First, selection signatures were explored using *F*_ST_ outliers, comparing them to the null distribution generated in windows of 100 kb, using VCFtools [28]. We used a windowed *F*_ST_ as a test statistic, retaining windows with values exceeding the 99% upper quantile as potential locations for selection. Since *F*_ST_ analysis does not differentiate between ancestor or domestic signatures of selection, we also used Selscan 1.1.0b [98] to implement extended haplotype homozygosity analysis (XP-EHH). XP-EHH scores were standardized across the genome and those exceeding the top 1% of the distribution were identified as potential signals of positive selection. Contiguous significant SNPs were integrated into a common signature or region within each comparison, allowing for one non-significant SNP and including half of the physical distance to the neighboring non-significant marker on both sides. Since XP-EHH searches for unusually long haplotypes, isolated significant SNPs were discarded. Positively selected regions were examined for the presence of genes of known adaptive significance first in llama and alpaca, then using the literature for other livestock species, and finally for evidence from model mammal species and humans. Finally, to search for putatively adaptively introgressed segments of non-native ancestry in the genomes of alpaca and llama, we compared the results of the selection signature analysis with the introgression analysis to look for common signatures and searched the genomes for sites where all sequenced alpacas and llamas were homozygous for introgressed guanaco or vicuña-like ancestry, respectively. We required that at least four consecutive markers support a region as introgressed. We then investigated fine-scale patterns of DNA variation in four regions containing the *ANTXR2*/*PRDM8/FGF5*/*C4orf22*, *EDN3*, *OR5* family, and *OR2* family genes to compare sequence variation in vicuña, guanaco, llama, and alpaca to both verify the ancestry of these segments and to examine *F*_ST_ distribution along the segments when comparing llama and alpaca. This was carried out to localize the regions of the gene responsible for the high overall *F*_ST_ and LAI scores and to examine their distribution to infer locus-specific selection processes in these genes.

### Functional analysis of target genes

To further investigate potential function of genes identified in the selection signature analysis, we first focused on a gene within our top ranking admixed locus (in the direction of llama to alpaca), comprising a set of candidate genes for adaptive introgression and artificial selection (*ANTXR2/PRDM8/FGF5*/*C4orf22*; see the “Results” section) [99, 100] and in particular on *FGF5*. Since functional variation at *FGF5* (also known as the angora gene) is known to correlate with short- or longwool phenotypes in sheep and goat [101, 102], we examined sequence differences in this gene in short and longwool alpaca. Six healthy 2 year-old longwool (Suri) alpacas (3:3) and shortwool (Huacaya) alpacas (3:3) were selected for mRNA and protein expression testing using the skin samples. A skin section (diameter 3 cm) from the neck was surgically obtained and immediately stored in liquid nitrogen. For mRNA PCR and qPCR, 1 μg of total RNA from Suri and Huacaya alpaca skins was extracted using TRIzol (Invitrogen) and treated with DNase I (Sigma). The mRNA was converted to cDNA using a cDNA synthesis kit (Takara, Dalian, China) for PCR and qPCR using SYBR Green (Takara) on a 7500 Fast Real-Time PCR system (Applied Biosystems) using *FGF5* primers (Additional file 1: Table S21). The PCR reaction is conducted as follows: 95 °C for 5 min, followed by 40 cycles of 95 °C 15 s, 56 °C or 58 °C for 30 s, and 72 °C for 30 s. PCR products were sequenced using standard Sanger methods and analyzed using an ABI 3730XL semi-automatic DNA analyzer. For Western Blot analysis, protein samples were separated using 10% SDS-PAGE and transferred to PVDF membranes. The membranes were blocked with 5% skimmed milk for 2 h and incubated for overnight at 4 °C with the diluted primary rabbit antibodies FGF5 (1:500) and β-actin (1:1000). After washing four times for 5 min each with TBST, the membranes were incubated for 1 h at 37 °C with horseradish peroxidase (HRP) secondary antibodies against rabbit IgG (1:10000). After washing four times for 5 min each with TBST, the bound antibodies were visualized by chemiluminescence using ECL. Immunoblots were scanned on a ChemiDOC™ XRS+ imager (Bio-Rad), and protein levels were quantified using Image-Pro Plus (Olympus). Finally, we examined the expression profiles (FPKM) of 20 single copy genes identified in the introgression and selection analysis (see above; Additional file 1: Table S22). Comparative inference from expression profiles was limited because of different tissue source (skin samples from 17 alpacas from China and blood samples from each of the de novo sequenced vicuña, guanaco, and llama).

To investigate the potential effect of hypoxia on the expression of the HSA4q21 syntenic locus (*ANTXR2/PRDM8/FGF5/C4orf22*), alpaca melanocytes and sheep melanocytes were cultured for 72 h under normoxic (21% O_2_) and hypoxic (13% O_2_) conditions respectively. Alpaca melanocytes and sheep melanocytes were lysed to get protein by RIPA (Beyotime, Shanghai, China) for Western Blot analysis with the diluted primary rabbit antibodies FGF5, ANTXR2, PRDM8, C4orf22 (all at a ratio of 1:500), and β-actin (1:1000) using the same protocol above described. Total RNA of alpaca melanocytes was extracted by Trizol (Invitrogen, Carlsbad, CA, USA) for qPCR analysis of *SOX6* and *HIF1*α (the primers were listed in Table S21).

## Supplementary information

**Additional file 1: Figure S1**. *K*-mer distribution. **Figure S2**. Detailed sampling map of all sequenced individual. **Figure S3**. *Treemix* phylogeny and variance plot. **Figure S4**. Introgression segments into llama (*f*_d_). **Figure S5**. PSMC plot for all four SAC species. **Figure S6**. MSMC plots for (a) guanaco; (b) llama; (c) vicuña; (d) alpaca. **Figure S7**. Manhattan plot of selection signatures detected in the comparison between vicuña and alpaca (XP-EHH upper, FST bottom). **Figure S8**. Manhattan plot of selection signatures detected in the comparison between guanaco and llama (XP-EHH upper, FST bottom). **Figure S9**. Comparison between region-wide FST and SNP distribution among wild ancestors and domestic relatives for the OR5 and OR2 olfactory receptor family. **Figure S10**. Comparison between region-wide FST and SNP distribution among wild ancestors and domestic relatives for the HoxD gene clusters and OLA1 gene related to morphology development. **Figure S11**. SNP distribution among wild ancestors and domestic relatives with evidence of introgression from llama to alpaca on Chr10. **Figure S12**. SNP distribution among wild ancestors and domestic relatives with evidence of introgression from alpaca to llama: CORIN, FGF21, GNAS, TAGLN. **Figure S13**. cDNA sequence, expression histogram, Western Blot and protein expression histogram for the alpaca skin FGF5 expression analysis. **Figure S14**. The phylogeny of South American camelids with segments of high guanaco ancestry removed from alpaca genomes. **Table S1**. Statistics of the clean data for the de novo genomes. **Table S2**a-c. Assembly statistics for each species. **Table S3**. Genome coverage assessed by transcriptome unigenes. **Table S4**. The BUSCO results of the three new assembled SAC genomes. **Table S5**. K-mer analysis. **Table S6**. Aligned sequence data for de novo genomes. **Table S6**a. Aligned sequence between the de novo genomes and related species: pairwise whole-genome alignment was performed using LASTZ. **Table S6**b. Synteny analysis for aligned de novo genomes. **Table S7**a-c. the number of predicted genes for each species. **Table S8**. Sampling details. **Table S9**. Resequencing data summary. **Table S10**. ABBA-BABA statistics. **Table S11**. Introgressed segments from llama into alpaca using fd and LAI. **Table S12**. Introgressed segments from alpaca into llama using fd and LAI. **Table S13**. Introgressed segment and genes showing low and high LAI introgression on the inferred X-chromosome of alpaca. **Table S14**. Selection signatures detected in comparisons between vicuña and alpaca (XP-EHH). **Table S15**. Selection signatures detected in comparisons between vicuña and alpaca (FST). **Table S16**. Selection signatures detected in comparisons between vicuña and alpaca (overlap between methods). **Table S17**. Selection signatures detected in comparisons between guanaco and llama (XP-EHH). **Table S18**. Selection signatures detected in comparisons between guanaco and llama (FST). **Table S19**. Selection signatures detected in comparisons between guanaco and llama (overlap between methods). **Table S20**. Olfactory receptor gene numbers in South American camelids, compared to the cow. **Table S21**. PCR and qPCR primers. **Table S22**. Blood and skin tissue expression counts for de novo sequenced SACs and Chinese alpaca, respectively. Supplementary Note S1 - De novo Genome Sequencing a. Genome size estimation. b. De novo sequencing, assembly and annotation. Supplementary Note S2 - Introgression analysis. Supplementary Note S3- Sample collection, DNA extraction and permits.

**Additional file 2.** Review history.

## Data Availability

The genome assembly generated and analyzed during the current study are available in the NCBI under BioProject ID PRJNA427832 [103], PRJNA421373 [104], and PRJNA427644 [105]. The SAC whole-genome shotgun project has been deposited at the GenBank under the accession number PRJNA612032 [106]. The RNA-seq data of Alpaca could be accessed through PRJNA636766 [107]. Other results are included in this published article and its Additional material files.

## Abbreviations

AlpGua: Alpaca genome that removed guanaco ancestry sites
AlpVic: Alpaca genome that only remain vicuña ancestry sites
ASIP: Agouti signaling protein
ANTXR: Anthrax toxin receptor
CORIN: Atrial natriuretic peptide-converting enzyme
BAM: Binary Alignment/Map
BP: Before present
BUSCO: Benchmarking Universal Single-Copy Orthologs
BLAT: BLAST-like Alignment Tool
BWA-MEM: Burrows-Wheeler Alignment MEM
CSTZ: Cathepsin Z
CDS: Coding sequence
C4orf22: Chromosome 4 open reading frame 22
ECL: Electrochemiluminescence
XP-EHH: Extended haplotype homozygosity
EDN3: Endothelin 3
FGF: Fibroblast growth factor
FPKM: Fragments Per Kilobase of transcript per Million mapped reads
GATK: Genome Analysis Toolkit
GO: Gene Ontology
HRP: Horseradish peroxidase
HIF: Hypoxia-inducible factor
PHD: Prolyl hydroxylase domain
SOX: Sry-related HMG-box-containing transcription factor
KEGG: Kyoto Encyclopedia of Genes and Genomes
LD: Linkage disequilibrium
ILS: Incomplete lineage sorting
LAI: Local Ancestry Inference
*f*_d_: Modified *f* statistics
MYA: Millions of years ago
MSMC: Multiple Sequence Markovian Coalescent
OR: Olfactory receptor
OLA1: Obg-like ATPase 1
PSMC: Pairwise Sequential Markovian Coalescent
PVDF: Polyvinylidene fluoride
PRDM8: PR domain containing 8
qPCR: Quantitative real-time PCR
QTL: Quantitative trait loci
SAMtools: Sequence Alignment/Map (SAM) tools
SNP: Single-nucleotide polymorphism
SACs: South American camelids
SNeP: SNP linkage disequilibrium-based *N*e estimation
TAGLN: Transgelin
VCFtools: Variant call format tools
GWAS: Whole-genome association studies
