# Supplementary material for: Genomic analysis of the domestication and post-Spanish conquest evolution of the llama and alpaca
Source: Genome Biol. 2020 Jul 2;21:159. doi: 10.1186/s13059-020-02080-6 (PMC7331169; doi:10.1186/s13059-020-02080-6)
Supplement: Supplementary file 1 — Additional file 1: Figure S1. K-mer distribution. Figure S2. Detailed sampling map of all sequenced individual. Figure S3. Treemix phylogeny and variance plot. Figure S4. Introgression segments into llama (fd). Figure S5. PSMC plot for all four SAC species. Figure S6. MSMC plots for (a) guanaco; (b) llama; (c) vicuña; (d) alpaca. Figure S7. Manhattan plot of selection signatures detected in the comparison between vicuña and alpaca (XP-EHH upper, FST bottom). Figure S8. Manhattan plot of selection signatures detected in the comparison between guanaco and llama (XP-EHH upper, FST bottom). Figure S9. Comparison between region-wide FST and SNP distribution among wild ancestors and domestic relatives for the OR5 and OR2 olfactory receptor family. Figure S10. Comparison between region-wide FST and SNP distribution among wild ancestors and domestic relatives for the HoxD gene clusters and OLA1 gene related to morphology development. Figure S11. SNP distribution among wild ancestors and domestic relatives with evidence of introgression from llama to alpaca on Chr10. Figure S12. SNP distribution among wild ancestors and domestic relatives with evidence of introgression from alpaca to llama: CORIN, FGF21, GNAS, TAGLN. Figure S13. cDNA sequence, expression histogram, Western Blot and protein expression histogram for the alpaca skin FGF5 expression analysis. Figure S14. The phylogeny of South American camelids with segments of high guanaco ancestry removed from alpaca genomes. Table S1. Statistics of the clean data for the de novo genomes. Table S2a-c. Assembly statistics for each species. Table S3. Genome coverage assessed by transcriptome unigenes. Table S4. The BUSCO results of the three new assembled SAC genomes. Table S5. K-mer analysis. Table S6. Aligned sequence data for de novo genomes. Table S6a. Aligned sequence between the de novo genomes and related species: pairwise whole-genome alignment was performed using LASTZ. Table S6b. Synteny analysis for aligned de novo ge [file 13059_2020_2080_MOESM1_ESM.docx]

**Additional Materials**

**Supplementary Figures**

1. Figure S1 - *K*-mer distribution
2. Figure S2 *-* Detailed sampling map of all sequenced individual
3. Figure S3 - Treemix phylogeny and variance plot
4. Figure S4 - Introgression segments into llama (*f*_d_)
5. Figure S5 - PSMC plot for all four SAC species
6. Figure S6 - MSMC plots for (a) guanaco; (b) llama; (c) vicuña; (d) alpaca
7. Figure S7 - Manhattan plot of selection signatures detected in the comparison between vicuña and alpaca (XP-EHH upper, *F*_ST_ bottom)
8. Figure S8 - Manhattan plot of selection signatures detected in the comparison between guanaco and llama (XP-EHH upper, *F*_ST_ bottom)
9. Figure S9 - Comparison between region-wide *F*_ST_ and SNP distribution among wild ancestors and domestic relatives for the *OR5* and *OR2* olfactory receptor family
10. Figure S10 - Comparison between region-wide *F*_ST_ and SNP distribution among wild ancestors and domestic relatives for the *HoxD* gene clusters and *OLA1* gene related to morphology development
11. Figure S11 - SNP distribution among wild ancestors and domestic relatives with evidence of introgression from llama to alpaca on Chr10
12. Figure S12 - SNP distribution among wild ancestors and domestic relatives with evidence of introgression from alpaca to llama: *CORIN*, *FGF21*, *GNAS*, *TAGLN*
13. Figure S13 - cDNA sequence, expression histogram, Western Blot and protein expression histogram for the alpaca skin *FGF5* expression analysis
14. Figure S14 - The phylogeny of South American camelids with segments of high guanaco ancestry removed from alpaca genomes

**Supplementary Tables**

1. Table S1 - Statistics of the clean data for the *de novo* genomes
2. Table S2a-c - Assembly statistics for each species
3. Table S3 - Genome coverage assessed by transcriptome unigenes
4. Table S4 - The BUSCO results of the three new assembled SAC genomes
5. Table S5 - *K*-mer analysis
6. Table S6 - Aligned sequence data for *de novo* genomes

Table S6a - Aligned sequence between the *de novo* genomes and related species: pairwise whole-genome alignment was performed using LASTZ

Table S6b - Synteny analysis for aligned *de novo* genomes

1. Table S7a-c - the number of predicted genes for each species
2. Table S8 - Sampling details
3. Table S9 - Resequencing data summary
4. Table S10 - ABBA-BABA statistics
5. Table S11 - Introgressed segments from llama into alpaca using *f_d_* and LAI
6. Table S12 - Introgressed segments from alpaca into llama using *f_d_* and LAI
7. Table S13 - Introgressed segment and genes showing low and high LAI introgression on the inferred X-chromosome of alpaca
8. Table S14 - Selection signatures detected in comparisons between vicuña and alpaca (XP-EHH)
9. Table S15 - Selection signatures detected in comparisons between vicuña and alpaca (*F*_ST_)
10. Table S16 - Selection signatures detected in comparisons between vicuña and alpaca (overlap between methods)
11. Table S17 - Selection signatures detected in comparisons between guanaco and llama (XP-EHH)
12. Table S18 - Selection signatures detected in comparisons between guanaco and llama (*F*_ST_)
13. Table S19 - Selection signatures detected in comparisons between guanaco and llama (overlap between methods)
14. Table S20 - Olfactory receptor gene numbers in South American camelids, compared to the cow
15. Table S21 - PCR and qPCR primers
16. Table S22 - Blood and skin tissue expression countsfor *de novo* sequenced SACs and Chinese alpaca, respectively

**Supplementary Text**

1. S1 *- De novo* Genome Sequencing

a. Genome size estimation

b. *De novo* sequencing, assembly and annotation

1. S2 - Introgression analysis
2. S3 - Sample collection, DNA extraction and permits

**Supplementary Figures**


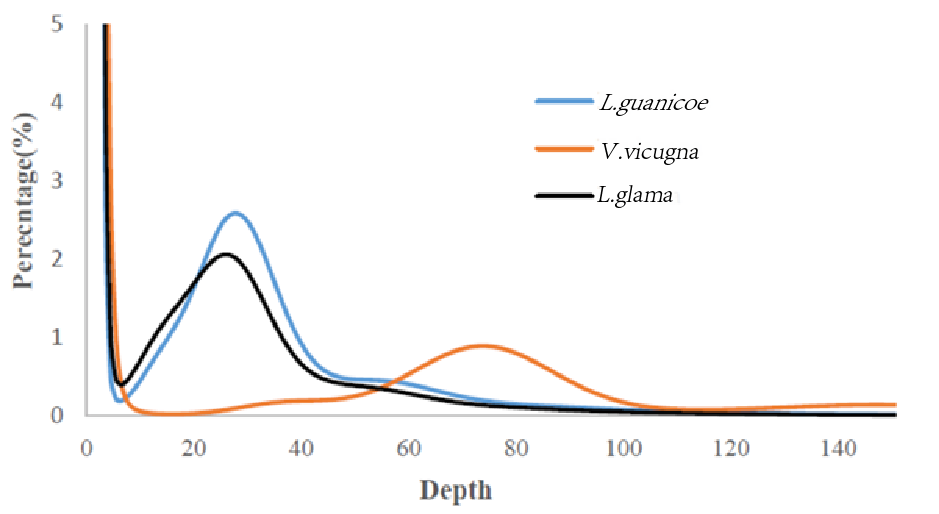


**Figure S1. *K*-mer distribution.** The *X*-axis represents the sequencing depth, the *Y*-axis represents the proportion of a *K*-mer count in total *K*-mer counts at a given sequencing depth. Estimated genome sizes for *Lama guanicoe*, *Vicugna vicugna* and *Lama glama* are 2.58 Gb, 2.60 Gb and 2.57 Gb, respectively.


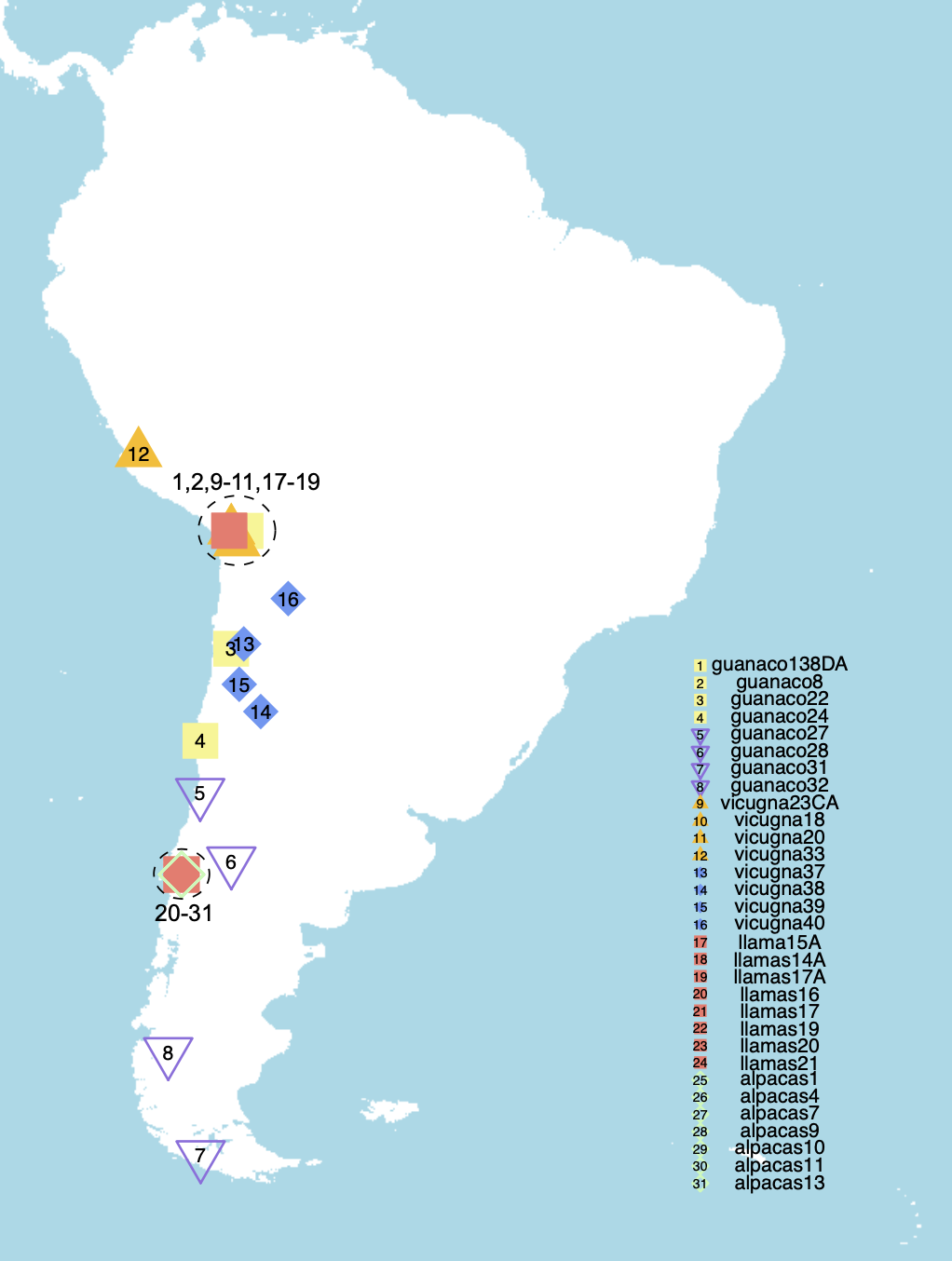


**Figure S2. Detailed sampling map of all sequenced individuals (*N* = 31)**.

a


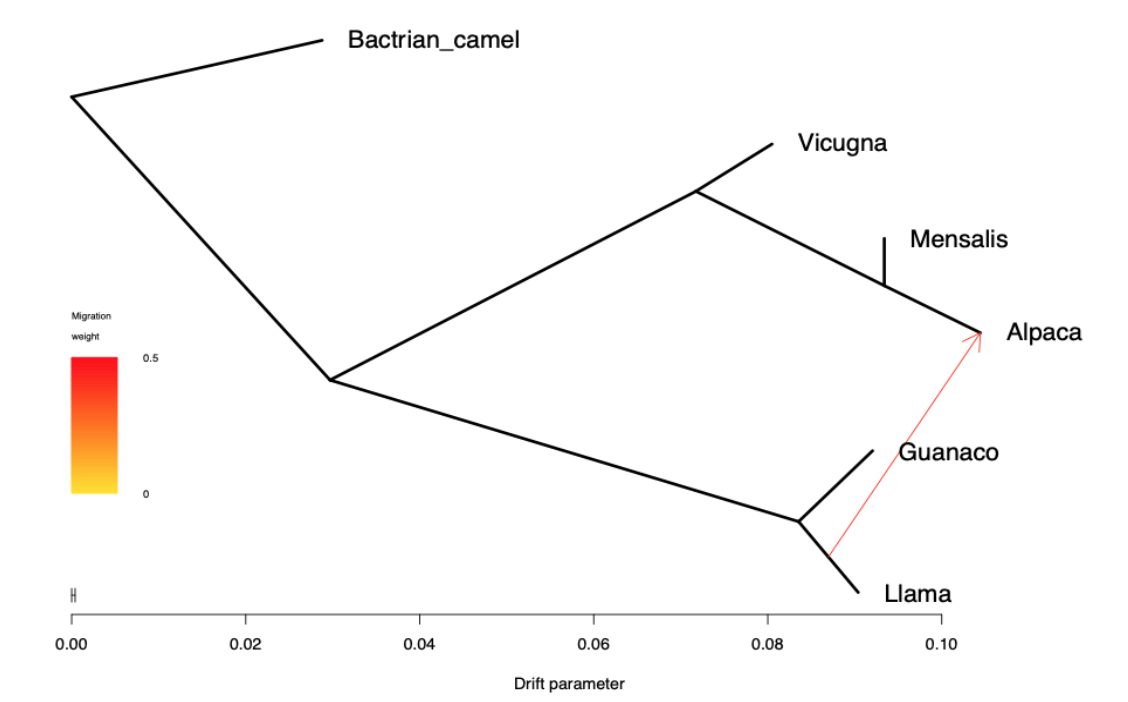


b


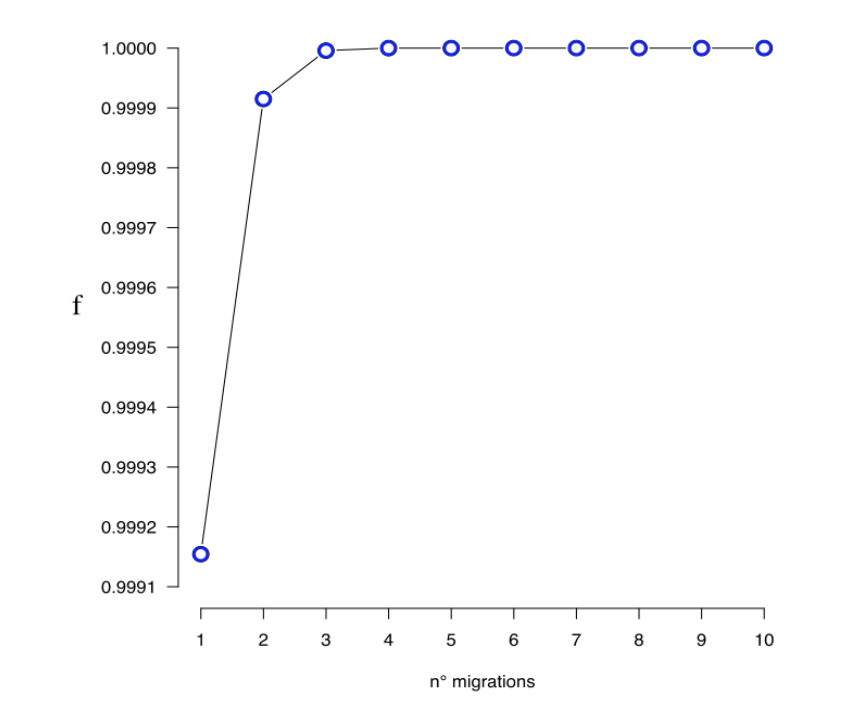


**Figure S3. Treemix phylogeny and variance plot.** a) Treemix phylogeny of South American Camelids showing unidirectional gene-flow from llama to alpaca and the relationship between alpaca and *V. v. mensalis*. b) *f* index representing the fraction of the variance, as a function of the number of modelled migration events.


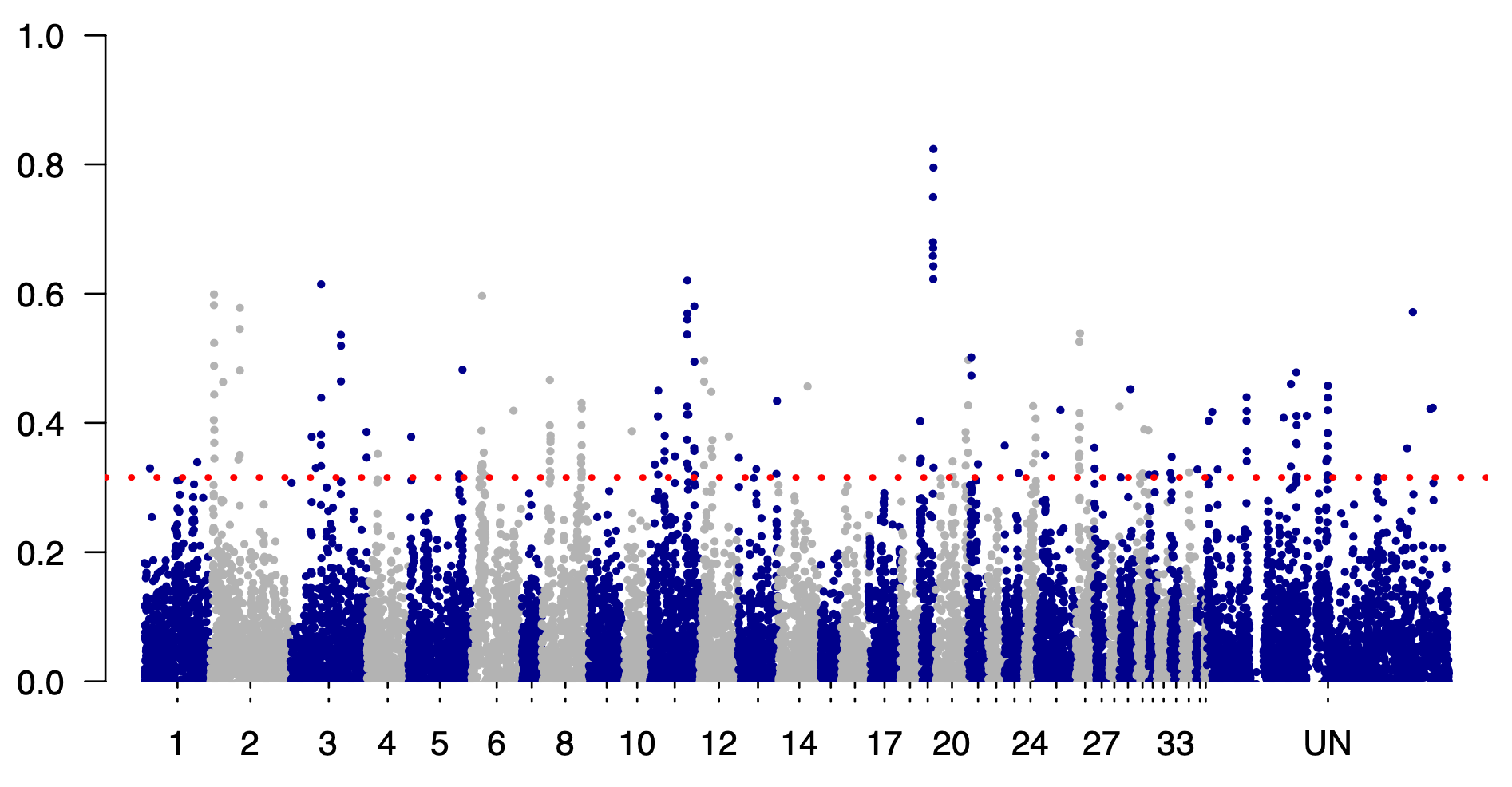


**Figure S4**. Manhattan Plot of **introgression segments into llama (***f*_d_**).** Middle line indicates mean introgression rate across the genome, red dotted line indicates the top 1% in terms of probability of llama ancestry. The X axis indicates the chromosome information and Y axis indicates the *f*_d_ statistics.


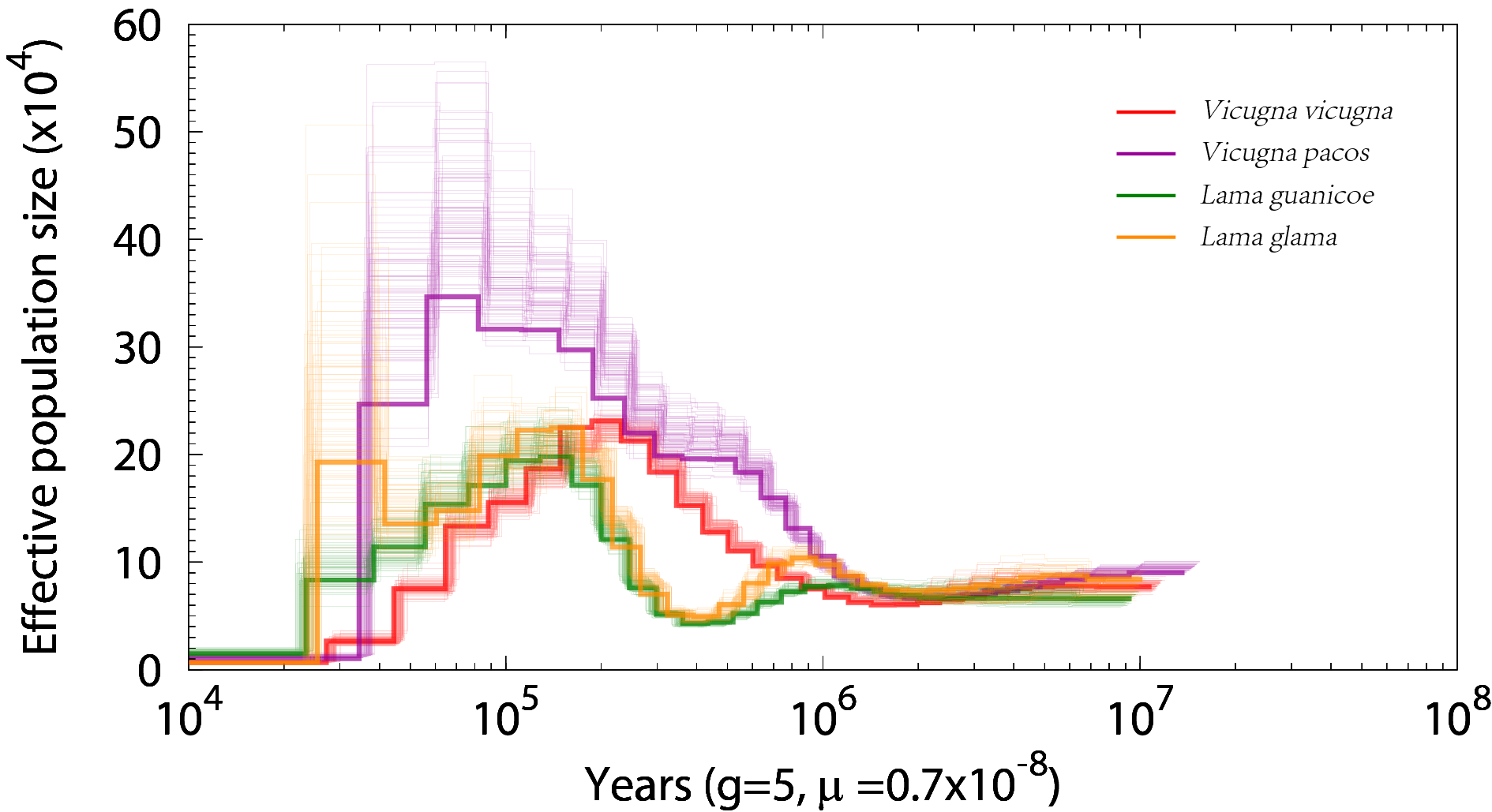


**Figure S5**. **Pairwise Sequential Markovian Coalescent (PSMC) plot of** **h****istorical changes in effective population size.** The reference genome sequenced in Wu *et al*.’s (2014) camelid study^1^ was refined in this study to remove regions of high llama ancestry (see main text methods).

**
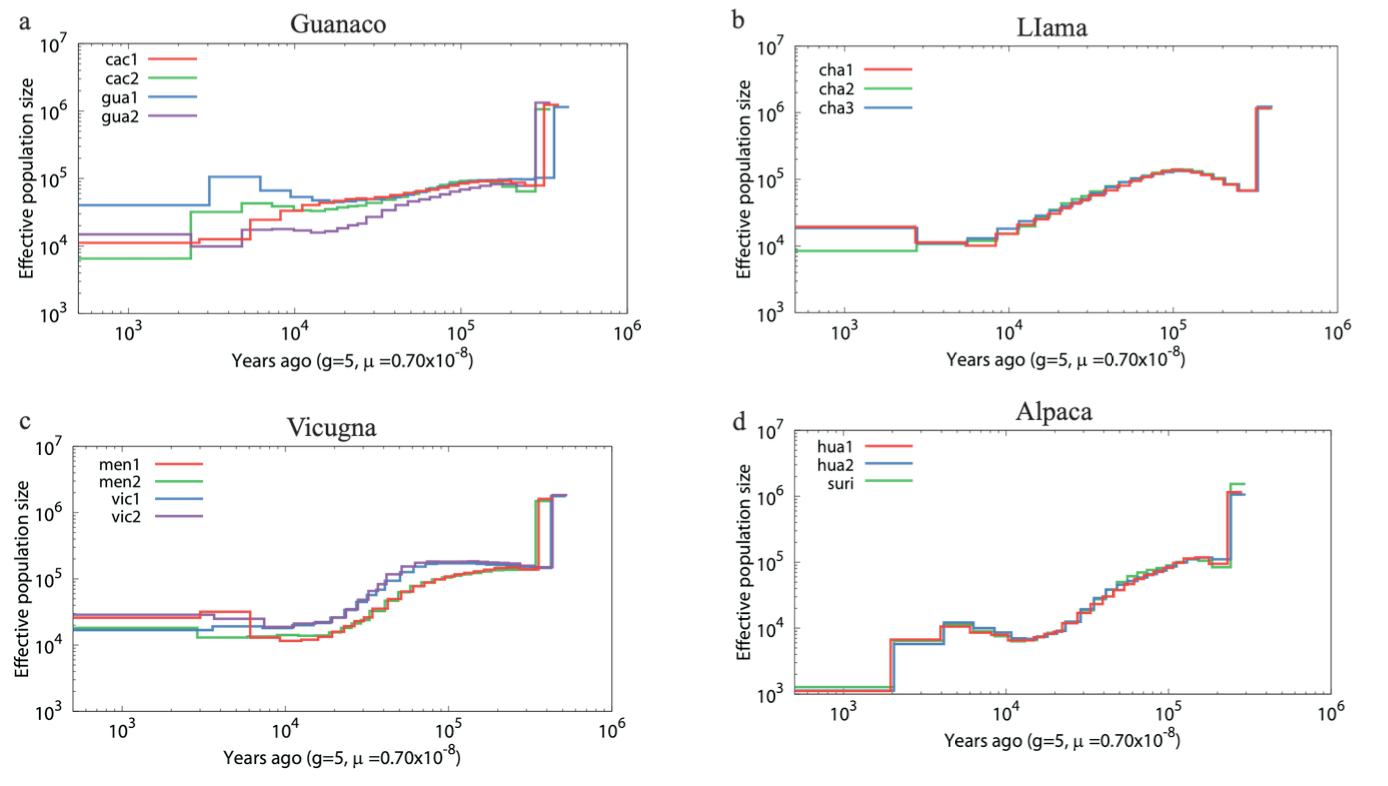
**

**Figure S6. Multiple sequentially Markovian coalescent(MSMC) plots historical changes in effective population size for guanaco, llama, vicuña and alpaca.** (a) guanaco (Individual guanaco138DA, 8,22, 24, 27, 28, 31, 32;see Table S8); (b) llama (llama16, 17,17A,19, 20, 21;see Table S8); (c) vicuña (vicugna23CA,18, 20, 33, 37, 38,39, 40: see Table S8); (d) alpaca (alpaca4,7, 9, 10,11, 13: see Table S8).

**
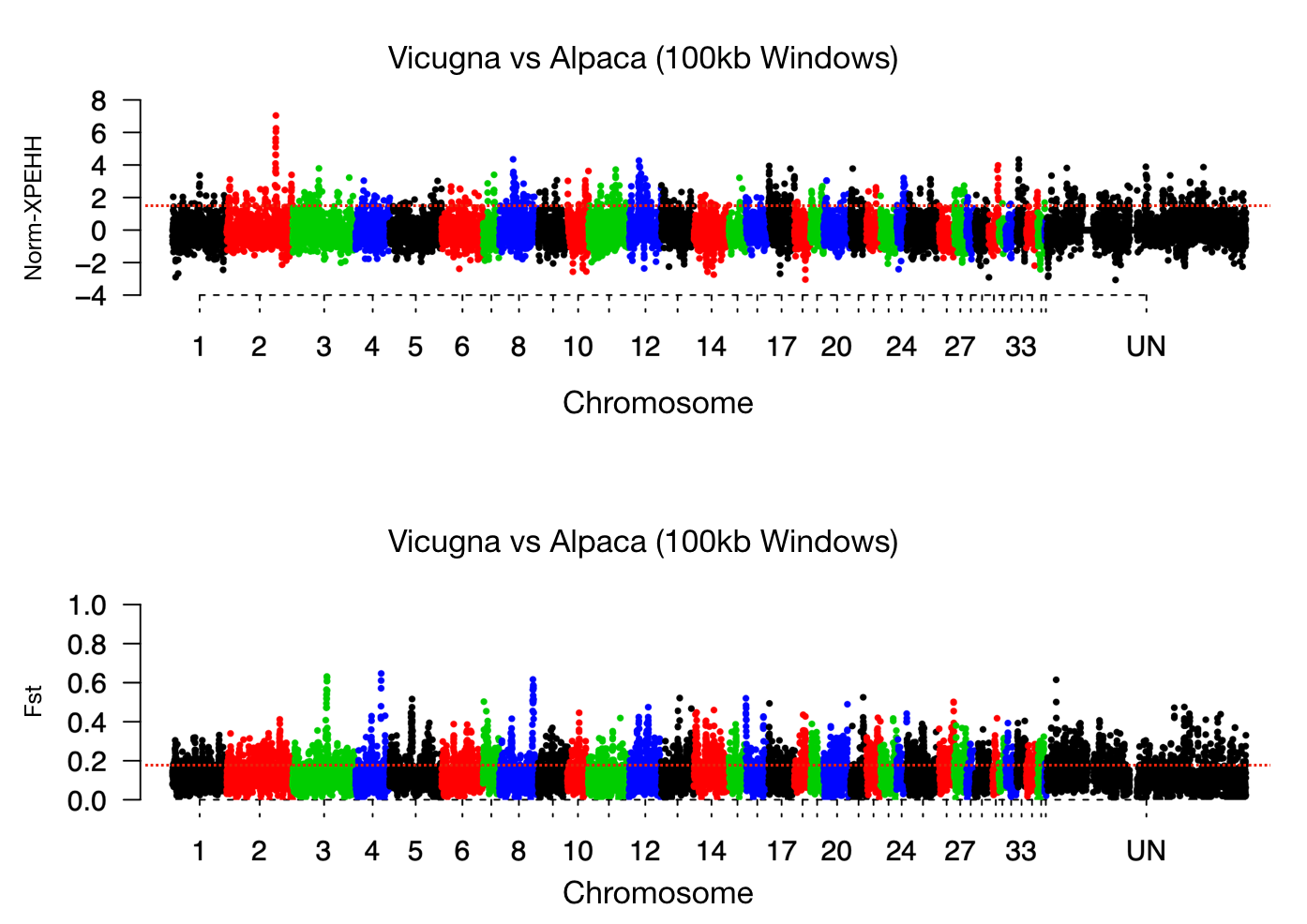
**

**Figure S7**. **Manhattan plot of selection signatures detected in the comparison between vicuña and alpaca (XPEHH upper, *F*_ST_ bottom).** The red dotted lines indicate the top 1% threshold.

**
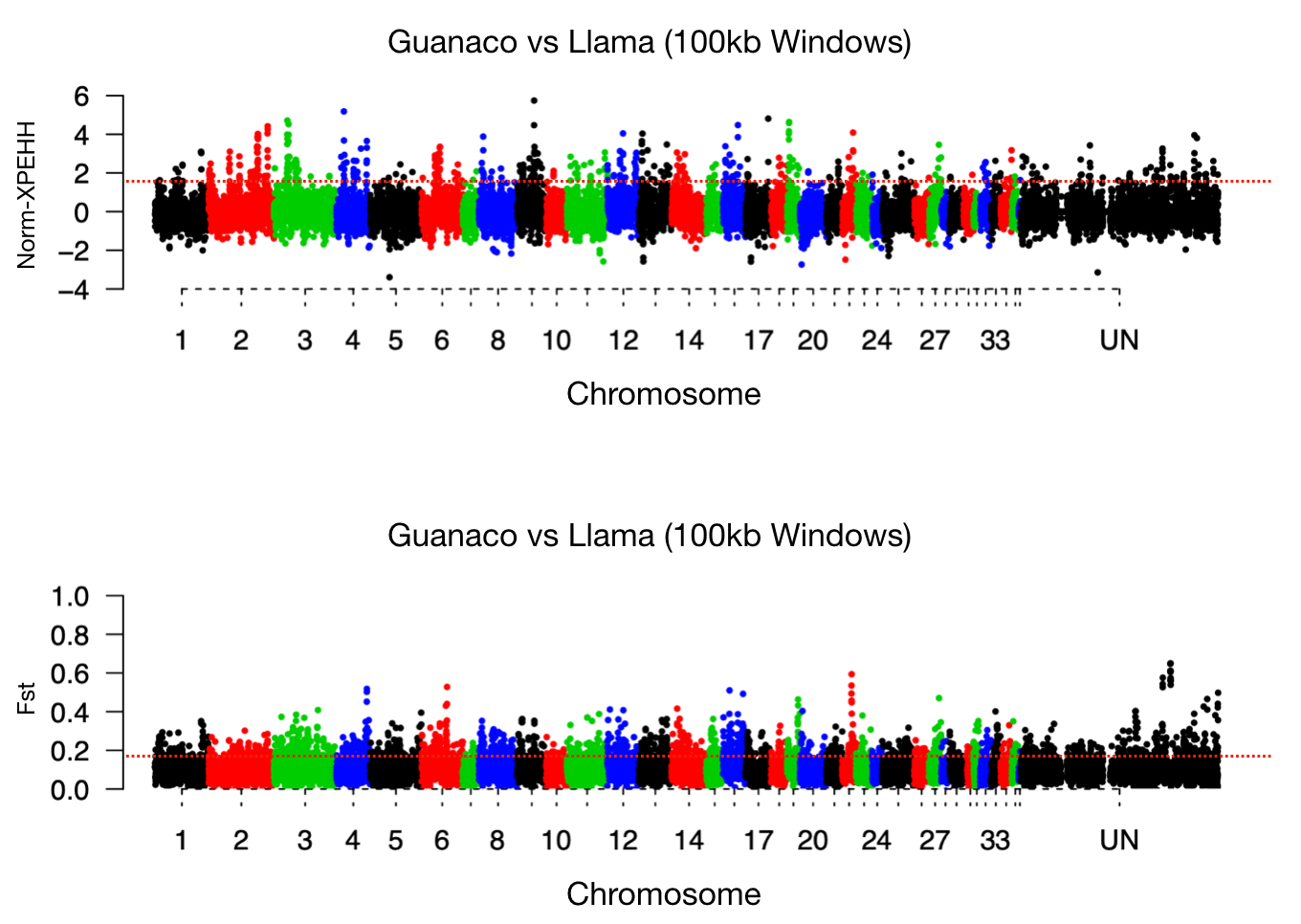
**

**Figure S8**. **Manhattan plot of selection signatures detected in the comparison between guanaco and llama (XPEHH upper, *F*_ST_ bottom).** The red dotted lines indicate the top 1% threshold.

**
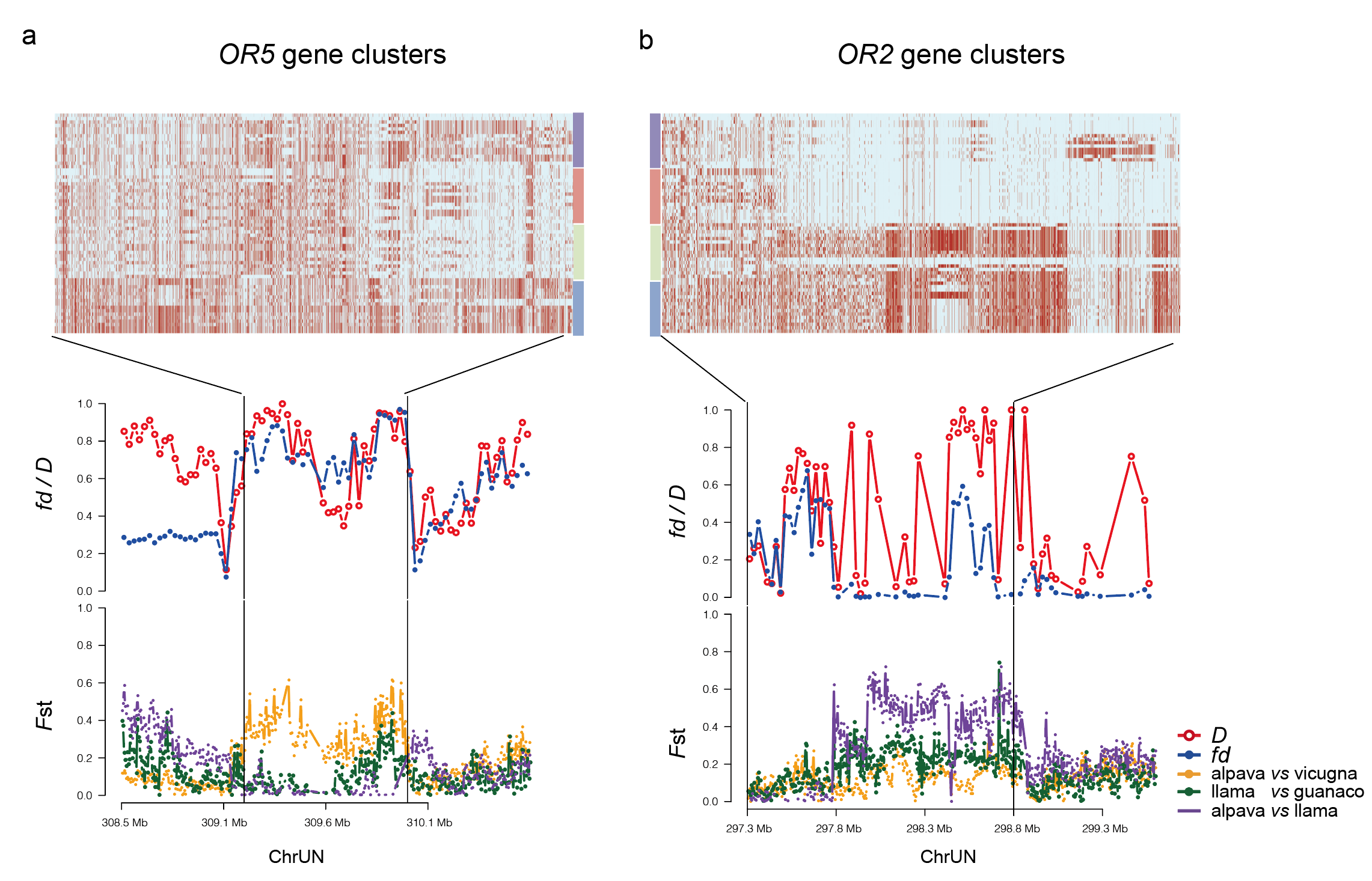
**

**Figure S9. Comparison between region-wide *F*_ST_ and SNP distribution among wild ancestors and domestic relatives for the *OR5* and *OR2* olfactory receptor family.** The haplotype heatmap among among camelid species for evidence of introgression from llama to alpaca (middle). The calculated *D* and *f_d_* statistics for ((*llama, guanaco), vicuña, Bactrian camel*) and the respective *F*_ST_ values for alpaca *versus* vicuña, llama *versus* guanaco and alpaca *versus* llama (bottom). Rectangles indicate the same allele with reference, while light blue rectangles indicate the alternative allele.

**
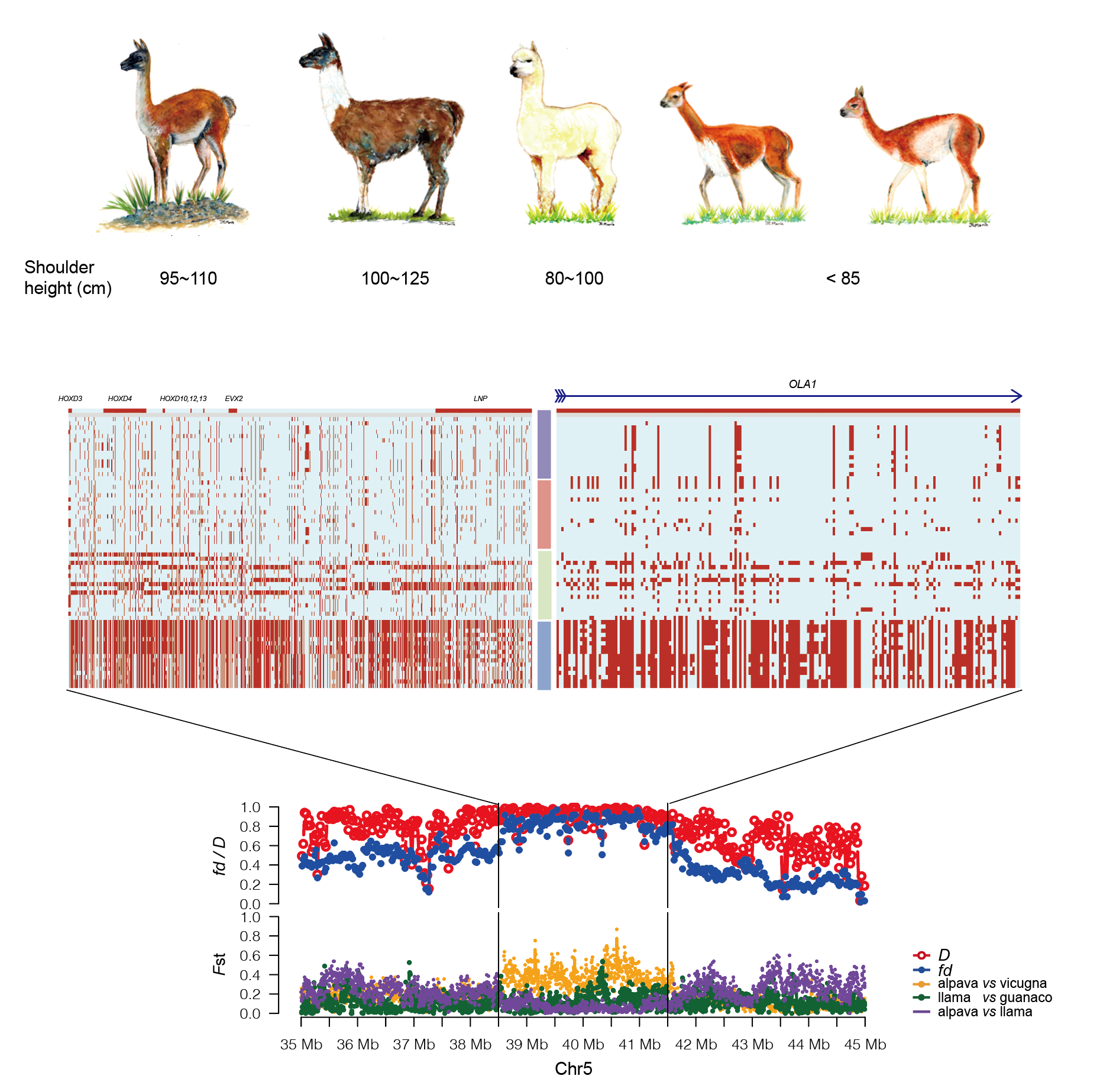
**

**Figure S10. Comparison between region-wide *F*_ST_ and haplotype heatmap among wild and domestic camelids for the *HOXD* gene cluster and *OLA1* gene related to morphology development.** Morphological difference among SACs (top). Haplotype heatmap among among SACs for the evidence of introgression from llama to alpaca (middle). *D* and *f_d_* statistics for ((*llama, guanaco), vicuña, Bactrian camel*) and respective *F*_ST_ values for alpaca *versus* vicuña, llama *versus* guanaco and alpaca *versus* llama (bottom). Red rectangles indicate the same allele with respect to the reference genome, while light blue rectangles indicate the alternative allele.

**
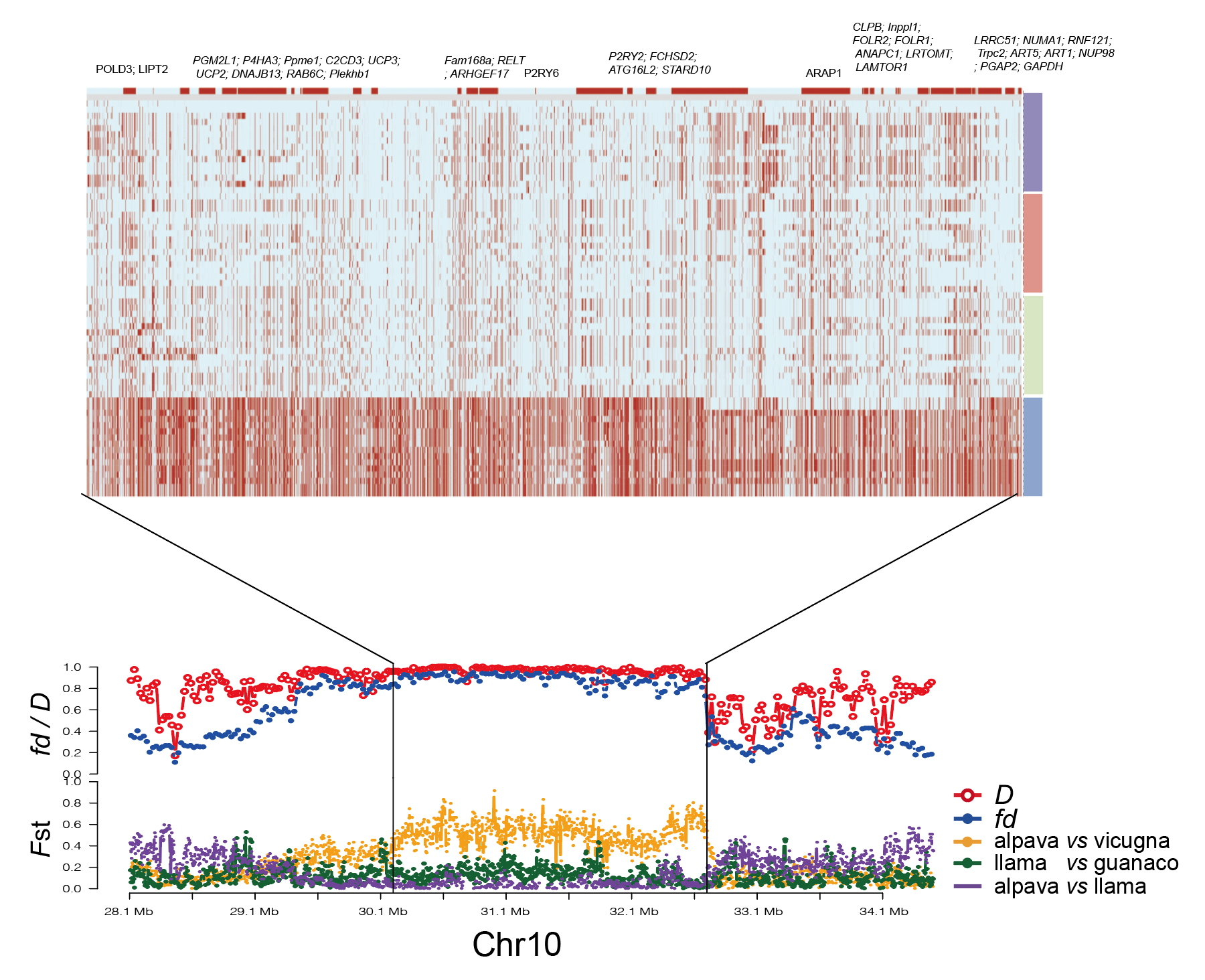
**

**Figure S11. The introgression pattern of represent region (~1.5 Mb) in Chromosome 10.** The haplotype heatmap among wild ancestors and domestic relatives for with evidence of introgression from llama to alpaca on Chr10 (top). The calculated *D* and *f_d_* statistics for ((*llama, guanaco), vicuña, Bactrian camel*) and the respective *F*_ST_ values for alpaca *versus* vicuña, llama *versus* guanaco and alpaca *versus* llama (bottom). The rectangles with red color indicate the same allele with reference, while light blue rectangles indicate the alternative allele.


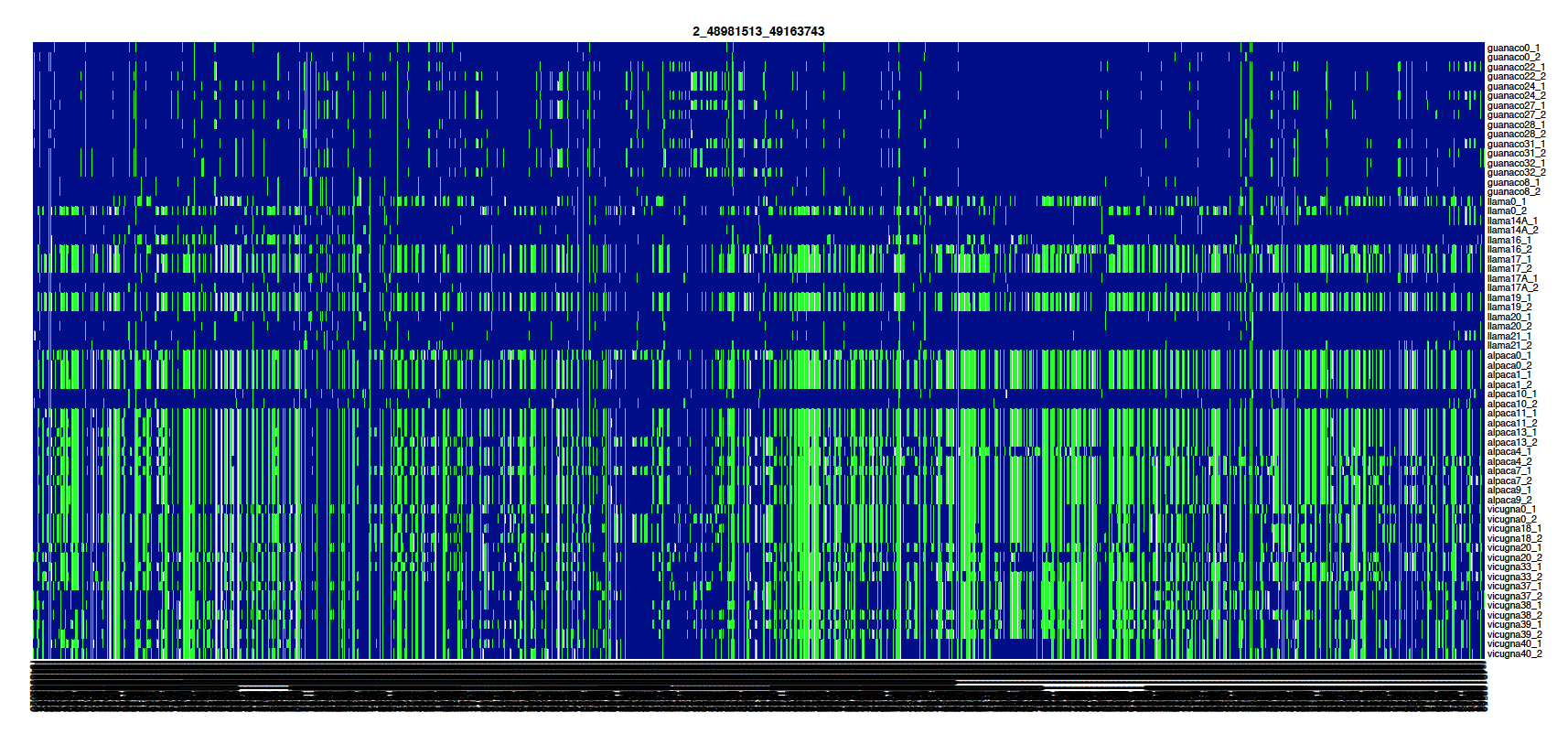

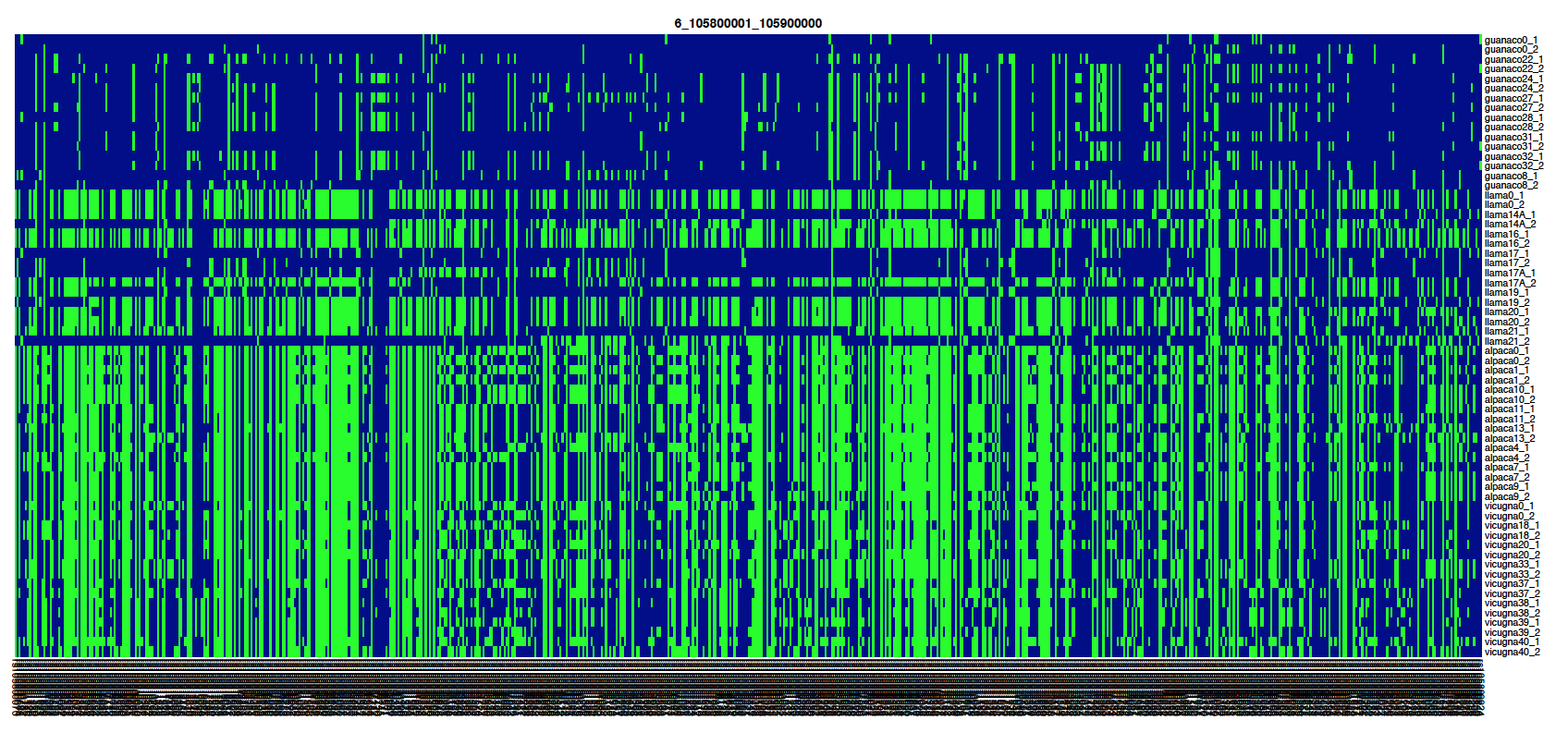

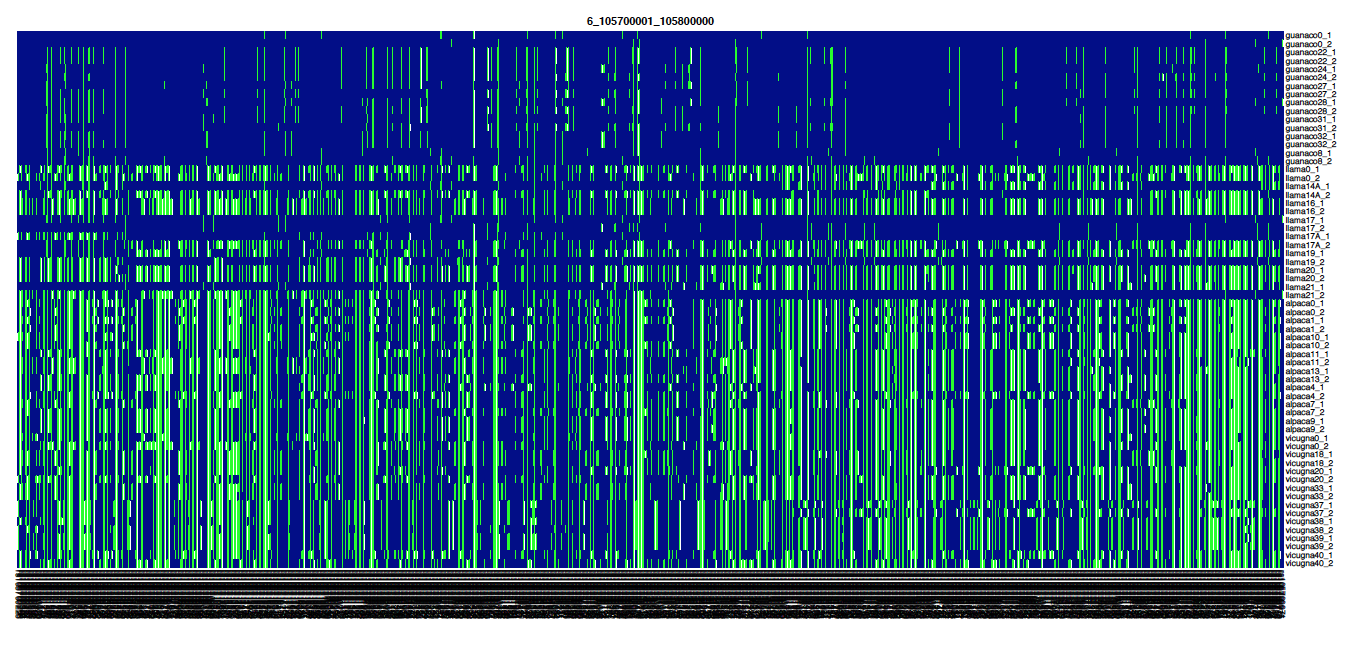

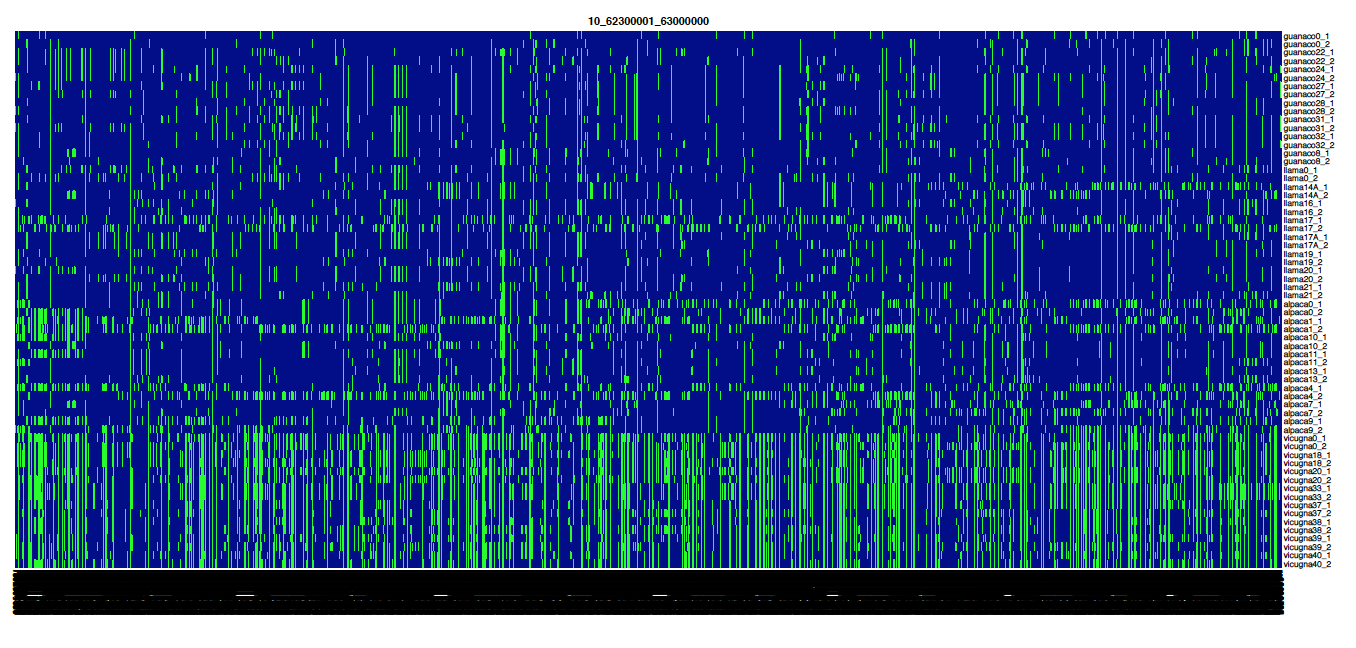


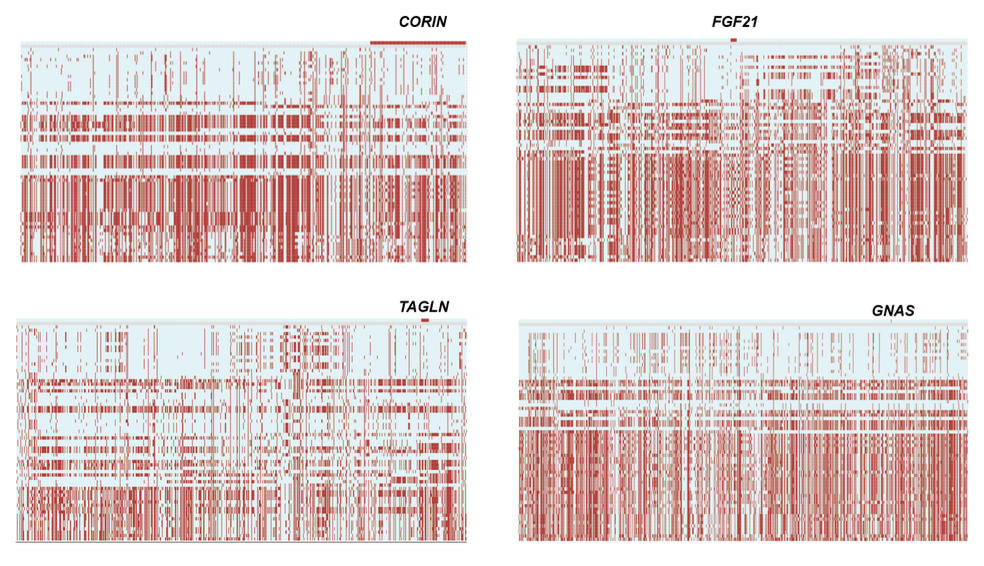


**Figure S12. The haplotype heatmap among wild ancestors and domestic relatives for with evidence of introgression from alpaca to llama: *CORIN*, *FGF21*, *TAGLN, GNAS.*** The rectangles with red color indicate the same allele with reference, while light blue rectangles indicate the alternative allele.

**
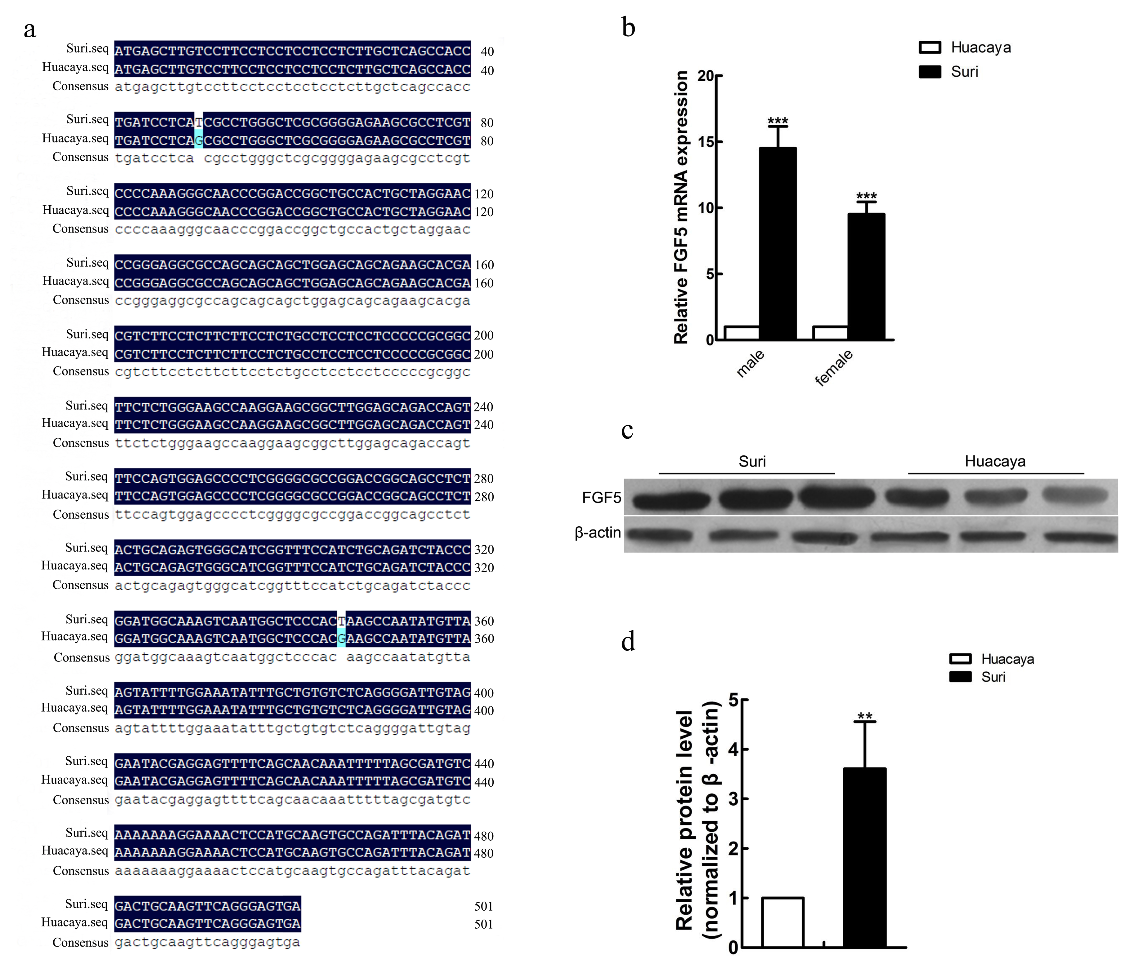
**

**Figure S13. cDNA sequence, expression histogram, Western Blot and protein expression histogram for the alpaca skin *FGF5* expression analysis .** (a) CDS region sequences of *FGF5* in Suri and Huacaya alpaca skins. (b) mRNA expression histogram of *FGF5* in Suri and Huacaya alpaca skins by qPCR. (c) Protein expression histogram of FGF5 in Suri and Huacaya alpaca skins by Western Blot. (d) Protein expression level of FGF5 in Suri and Huacaya alpaca skins. All samples were run in triplicate and the relative mRNA and protein expression was normalized to the expression levels of 18s mRNA and β-actin (1:1000), respectively. The difference in abundance of FGF5 was determined by the variance analysis (SPSS 11.5 software, Chicago, IL).

**
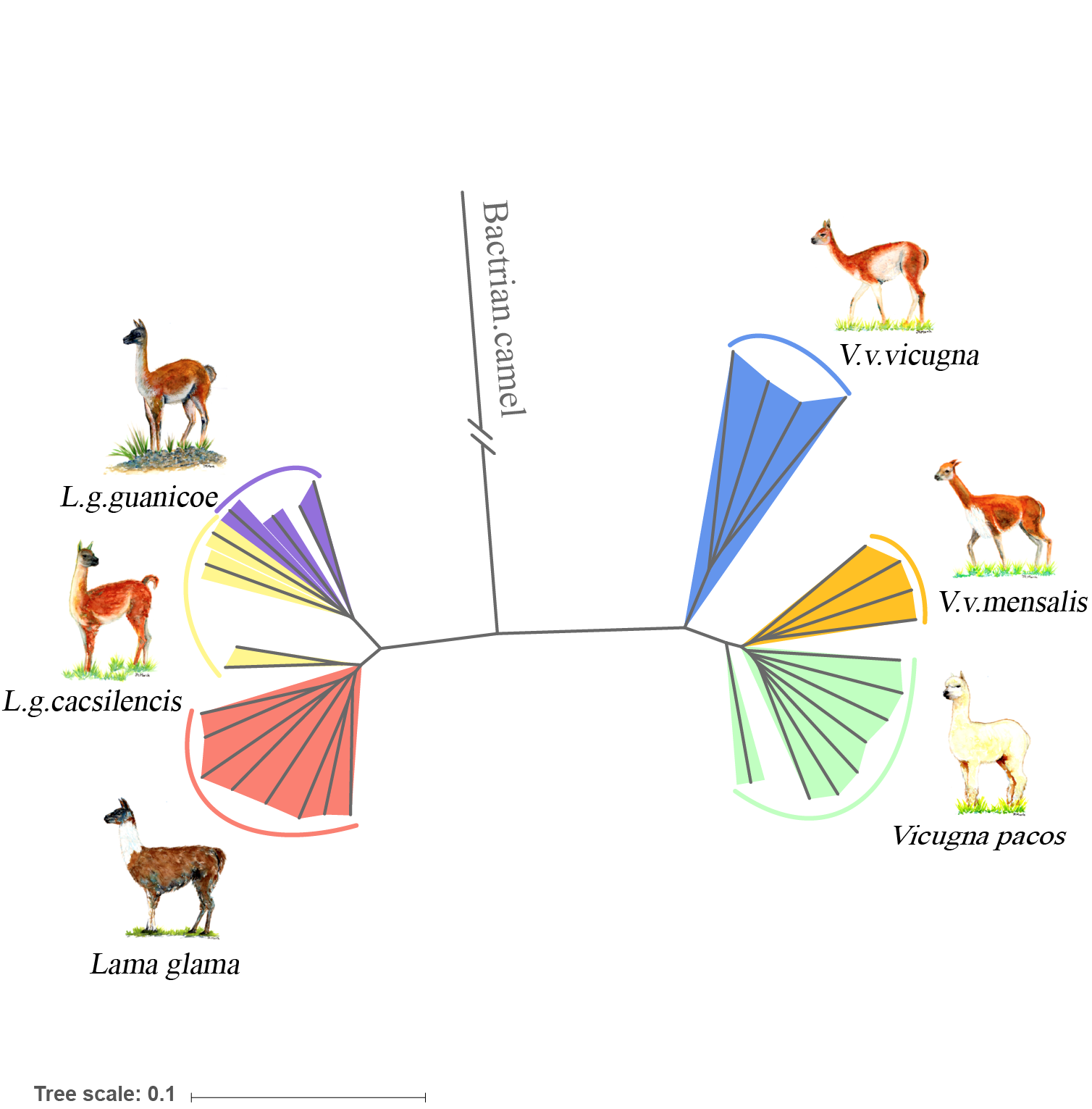
**

**Figure S14. Phylogeny of South American camelids with segments of high probability guanaco ancestry removed from alpaca genomes(retaining 64% of the original sequence).**

**Table S1. Statistics of the clean data for the *de novo* genomes.**Genomic DNA libraries with varied insert sizes were constructed.

| Species | Platform | Insert Size | Read Length (bp) | Total Data (Gb) | Sequence Depth (x) | Physical Depth (x) |
| --- | --- | --- | --- | --- | --- | --- |
| *Lama guanicoe* |  | 170bp | 125 | 109.55 | 42.14 | 28.66 |
|  | Hiseq 2000 | 500bp | 125 | 104.43 | 40.17 | 80.34 |
|  |  | 800bp | 125 | 46.65 | 17.94 | 57.47 |
|  |  | 2Kb | 49 | 26.65 | 10.25 | 209.18 |
|  |  | 5Kb | 49 | 20.61 | 7.93 | 404.59 |
|  |  | 10Kb | 49 | 11.1 | 4.27 | 435.71 |
|  |  | 20Kb | 49 | 9.5 | 3.65 | 744.9 |
|  |  | 40Kb | 49 | 7.18 | 2.76 | 1126.53 |
|  | Total |  |  | 335.67 | 129.1 | 3087.38 |
| *Vicugna vicugna* | Hiseq 2000 | 170bp | 125 | 99.21 | 38.16 | 25.95 |
|  |  | 500bp | 125 | 67.18 | 25.84 | 51.68 |
|  |  | 800bp | 125 | 48.72 | 18.74 | 59.96 |
|  |  | 2Kb | 49 | 25.87 | 9.95 | 203.06 |
|  |  | 5Kb | 49 | 19.88 | 7.65 | 390.11 |
|  |  | 10Kb | 49 | 7.79 | 3 | 305.73 |
|  |  | 20Kb | 49 | 6.36 | 2.45 | 499.22 |
|  |  | 40Kb | 49 | 7.18 | 2.76 | 1127.16 |
|  | Total | ---- | ---- | 282.19 | 108.53 | 2662.86 |
| *Lama glama* | Hiseq 2500 | 270bp | 150 | 84.33 | 32.44 | 29.2 |
|  |  | 800bp | 125 | 108.57 | 41.76 | 133.63 |
|  |  | 2kb | 48 | 28.91 | 11.12 | 231.67 |
|  |  | 5kb | 48 | 29.84 | 11.48 | 597.92 |
|  |  | 10kb | 48 | 13.48 | 5.19 | 540.63 |
|  | Total | -- | -- | 265.13 | 101.97 | 1533.05 |

**Table 2**. **Assembly statistics for each species**

**Table S2a. Assembly statistics for *Lama guanicoe*.**

|  | Contig  Size (bp) Number | | Scaffold  Size (bp) Number | |
| --- | --- | --- | --- | --- |
| N90 | 17,946 | 24,934 | 1,583,091 | 192 |
| N80 | 36,446 | 17,068 | 4,417,026 | 122 |
| N70 | 53,649 | 12,414 | 7,082,961 | 85 |
| N60 | 71,503 | 9,074 | 10,145,317 | 61 |
| N50 | 91,651 | 6,507 | 14,639,450 | 44 |
| Longest | 803,867 | ---- | 2,782,636 | ---- |
| Total Size | 2,073,525,543 | ---- | 2,086,935,219 | ---- |
| Total Number(≥100bp) | ---- | 382,544 | ---- | 324,321 |
| Total Number(≥2kb) | ---- | 42,554 | ---- | 3,624 |

**Table S2b**. **Assembly statistics for *Vicugna vicugna***

|  | Contig  Size (bp) Number | | Scaffold  Size (bp) Number | |
| --- | --- | --- | --- | --- |
| N90 | 22,394 | 23,079 | 912,915 | 393 |
| N80 | 39,467 | 16,449 | 2,187,903 | 253 |
| N70 | 55,400 | 12,170 | 3,396,254 | 178 |
| N60 | 72,272 | 8,998 | 4,853,305 | 130 |
| N50 | 91,189 | 6524 | 6,158,010 | 93 |
| Longest | 627,215 | ---- | 40,737,153 | ---- |
| Total Size | 2,012,487,251 | ---- | 2,026,893,968 | ---- |
| Total Number(≥100bp) | ---- | 73,066 | ---- | 12,432 |
| Total Number(≥2kb) | 22,394 | 23,079 | 912,915 | 393 |

**Table S2c**. **Assembly statistics for *Lama glama***

|  | Contig  Size (bp) Number | | Scaffold  Size (bp) Number | |
| --- | --- | --- | --- | --- |
| N90 | 10,523 | 48,630 | 542,334 | 749 |
| N80 | 19,047 | 34,546 | 1,203,272 | 501 |
| N70 | 27,039 | 25,619 | 1,771,832 | 360 |
| N60 | 35,214 | 19,027 | 2,414,184 | 261 |
| N50 | 44,134 | 13,858 | 3,218,230 | 186 |
| Longest | 333,241 | ---- | 16,767,056 | ---- |
| Total Size | 2,036,633,356 | ---- | 2,060,869,063 | ---- |
| Total Number(≥100bp) | ---- | 332,930 | ---- | 244,170 |
| Total Number(≥2kb) | ---- | 73,110 | ---- | 5,034 |

**Table S3**. **Genome coverage assessed by transcriptome unigenes**

| Species | Dataset | Number | Total length (bp) | Covered by assembly (%) | with >90% sequence in one scaffold | | with >50% sequence in one scaffold | |
| --- | --- | --- | --- | --- | --- | --- | --- | --- |
|  |  |  |  |  | Number | Percent (%) | Number | Percent (%) |
| *Lama guanicoe* | >0bp | 123567 | 126875406 | 98.84 | 119715 | 96.88 | 123331 | 99.81 |
|  | >200bp | 93837 | 121779727 | 98.82 | 90863 | 96.83 | 93644 | 99.79 |
|  | >500bp | 50518 | 108800395 | 98.76 | 48491 | 95.99 | 50396 | 99.76 |
|  | >1000bp | 35556 | 98198733 | 98.71 | 33971 | 95.54 | 35464 | 99.74 |
| *Vicugna vicugna* | >0bp | 204916 | 95306349 | 87.96 | 182908 | 89.26 | 187343 | 91.42 |
|  | >200bp | 126658 | 81942149 | 87.15 | 111338 | 87.9 | 114332 | 90.27 |
|  | >500bp | 36524 | 55861460 | 85.46 | 30680 | 84 | 31852 | 87.21 |
|  | >1000bp | 17879 | 43060468 | 84.81 | 14687 | 82.15 | 15402 | 86.15 |
| *Lama glama* | >0bp | 593,388 | 353,106,158 | 98.35 | 568,958 | 95.88 | 589,096 | 99.28 |
|  | >200bp | 412,499 | 322,014,630 | 98.29 | 393,324 | 95.35 | 409,466 | 99.26 |
|  | >500bp | 141,041 | 241,623,168 | 98.12 | 131,768 | 93.43 | 139,689 | 99.04 |
|  | >1000bp | 74,673 | 195,906,489 | 98.04 | 69,183 | 92.65 | 73,879 | 98.94 |

**Table S4. The BUSCO results of the three new assembled SAC species**

| Camels | Complete | Fragmented | Missing |
| --- | --- | --- | --- |
| Guanicoe | 94.70% | 3.30% | 2.00% |
| Glama | 93.70% | 3.90% | 2.40% |
| Vicugna | 95.80% | 2.00% | 2.20% |

**Table S5**. ***K*-mer analysis of the SAC genomes.**

| Species | *K* | *K*-mer  number | Peak  depth | Genome size | Bases used | Used reads | Coverage |
| --- | --- | --- | --- | --- | --- | --- | --- |
| *Lama guanicoe* | 17 | 67,200,000,000 | 26 | 2,584,615,384 | 80,000,000,000 | 800,000,000 | 31 |
| *Vicugna vicugna* | 17 | 187,346,919,393 | 72 | 2,602,040,547 | 214,847,384,625 | 1,718,779,077 | 83 |
| *Lama glama* | 17 | 64,244,213,848 | 25 | 2,569,768,553 | 78,134,854,680 | 868,165,052 | 30 |

**Table S6. Aligned sequence data for *de novo* genomes**

**Table S6a**. **Aligned sequence between the *de novo* genomes and related species: pairwise whole-genome alignment was performed using LASTZ.**

| Query_species *vs* Target_species | Number of syntenic segments | Average length of syntenic segments |
| --- | --- | --- |
| *V. pacos vs C. bactrianus* | 1,656,031 | 791.58 |
| *V. pacos vs C. dromedarius* | 1,670,422 | 785.33 |
| *V. pacos vs H. sapiens* | 2,215,344 | 457.90 |
| *V. pacos vs L. glama* | 1,550,134 | 862.13 |
| *V. pacos vs L. guanicoe* | 1,640,973 | 826.18 |
| *L. guanicoe vs C. bactrianus* | 1,668,743 | 773.16 |
| *L. guanicoe vs C. dromedarius* | 1,669,077 | 768.42 |
| *L. guanicoe vs H. sapiens* | 2,133,358 | 458.73 |
| *V. vicugna vs C. bactrianus* | 1,618,724 | 790.19 |
| *V. vicugna vs C. dromedarius* | 1,650,899 | 777.75 |
| *V. vicugna vs H. sapiens* | 2,175,076 | 459.02 |
| *V. vicugna vs L. guanicoe* | 1,578,710 | 841.44 |
| *V. vicugna vs V. pacos* | 1,516,416 | 871.186 |
| *L. glama vs C. bactrianus* | 1,574,972 | 818.86 |
| *L. glama vs C. dromedarius* | 1,610,460 | 823.37 |
| *L. glama vs H. sapiens* | 2,236,162 | 456.48 |
| *L. glama vs L. guanicoe* | 1,517,986 | 875.11 |
| *L. glama vs V. pacos* | 1,550,134 | 862.13 |
| *L. glama vs V. vicugna* | 1,521,136 | 859.16 |
| *V. pacos vs B. taurus* | 2,386,058 | 437.028 |
| *V. pacos vs O. aries* | 2,341,563 | 439.75 |
| *L. guanicoe vs B. taurus* | 2,270,751 | 439.26 |
| *L. guanicoe vs O. aries* | 2,238,269 | 441.098 |
| *V. vicugna vs B. taurus* | 2,317,376 | 439.99 |
| *V. vicugna vs O. aries* | 2,283,524 | 440.88 |
| *L. glama vs B. taurus* | 2,409,718 | 438.91 |
| *L. glama vs O. aries* | 2,363,906 | 440.18 |

**Table S6b. Synteny between *C. bactrianus, L. guanicoe and V.vicugna***

| Species | Total syntenic length (bp) | Number of Genes50 | Percentage | Number of Genes70 | Percentage |
| --- | --- | --- | --- | --- | --- |
| *C. dromedarius* | 1,227,054,638 | 17,907 | 88.45% | 12,480 | 61.62% |
| *L. glama* | 1,176,815,446 | 16,287 | 75.89% | 10,028 | 46.7% |
| *L. guanicoe* | 1,110,721,184 | 15,330 | 71.51% | 10,320 | 48.14% |
| *V. vicugna* | 1,161,319,807 | 16,120 | 74.09% | 10,316 | 47.41% |
| *V. pacos* | 1,171,568,387 | 15,823 | 75.83% | 10,147 | 48.63% |

Number of Genes50 = the number of genes with 50% coverage overlapping with syntenic regions, Number of Genes70 = 70% coverage.

**Table S7**. **The number of predicted genes for each species**

**Table S7a. The number of predicted genes for *Lama guanicoe***

| Annotation methods | | Gene  number | Average  gene  length | Average CDS length | Average  Exon number | Average  Exon length | Average intron length |
| --- | --- | --- | --- | --- | --- | --- | --- |
| *de novo* | AUGUSTUS | 27,424 | 21,142.33 | 1,230.13 | 6.59 | 186.59 | 3,560.37 |
|  | GeneScan | 47,506 | 30,014.14 | 1,287.99 | 7.87 | 163.75 | 4,184.17 |
| Homology | *Bos taurus* | 20,406 | 23,116.50 | 1,528.34 | 8.61 | 177.49 | 2,836.46 |
|  | *Camelus bactrianus* | 21,117 | 24,638.85 | 1,529.95 | 8.78 | 174.21 | 2,969.38 |
|  | *Camelus dromedarius* | 21,186 | 24,667.21 | 1,524.14 | 8.77 | 173.88 | 2,980.26 |
|  | *Equus caballus* | 21,022 | 22,345.78 | 1,456.41 | 8.25 | 176.54 | 2,881.34 |
|  | *Homo sapiens* | 20,056 | 25,174.09 | 1,569.24 | 8.82 | 177.98 | 3,019.73 |
|  | *Mus musculus* | 20,088 | 24,528.51 | 1,544.12 | 8.62 | 179.03 | 3,014.35 |
|  | *Vicugna pacos* | 20,508 | 25,369.62 | 1,538.45 | 8.9 | 172.86 | 3,016.66 |
| RNA-seq | | 119,824 | 16,944.57 | 1,079.37 | 3.72 | 290.24 | 5,835.14 |
| Glean | | 21,435 | 24,599.35 | 1,529.73 | 8.39 | 182.27 | 3,120.60 |

**Table S7b**. **The number of predicted genes for *Vicugna vicugna***

| Annotation methods | | Gene  number | | Average  gene  length | Average CDS length | | Average  Exon number | | Average  Exon length | Average intron length |
| --- | --- | --- | --- | --- | --- | --- | --- | --- | --- | --- |
| *de novo* | AUGUSTUS | | 28,971 | 19,445.25 | 1,180.75 | 6.22 | | 189.84 | | 3,499.03 |
|  | GeneScan | | 47,563 | 29,515.32 | 1,275.81 | 7.8 | | 163.51 | | 4,151.35 |
| Homology | *Bos taurus* | | 20,315 | 22,965.36 | 1,531.13 | 8.62 | | 177.64 | | 2,813.22 |
|  | *Camelus*  *bactrianus* | | 20,972 | 24,578.98 | 1,535.41 | 8.81 | | 174.28 | | 2,950.53 |
|  | *Camelus dromedarius* | | 20,885 | 24,597.36 | 1,527.29 | 8.79 | | 173.72 | | 2,960.79 |
|  | *Equus*  *caballus* | | 20,847 | 22,230.08 | 1,461.13 | 8.28 | | 176.46 | | 2,852.84 |
|  | *Homo sapiens* | | 20,131 | 24,997.41 | 1,565.92 | 8.81 | | 177.71 | | 2,999.64 |
|  | *Mus musculus* | | 20,006 | 24,439.34 | 1,547.05 | 8.64 | | 179.02 | | 2,995.74 |
|  | *Vicugna pacos* | | 20,296 | 25,250.08 | 1,541.50 | 8.92 | | 172.77 | | 2,992.73 |
| RNA-seq | | 119,824 | | 179,097 | 6,302.52 | | 480.33 | | 247.78 | 6,203.52 |
| Glean | | 21,435 | | 21,757 | 24,260.03 | | 1,522.19 | | 183.54 | 3,117.48 |

**Table S7c**. **The number of predicted genes for *Lama glama***

| Annotation methods | | Gene  number | Average  gene  length | Average CDS length | Average  Exon number | | Average  Exon length | | Average intron length |
| --- | --- | --- | --- | --- | --- | --- | --- | --- | --- |
| *de novo* | AUGUSTUS | 28,555 | 17,220.50 | 1,186.09 | 6.4 | 185.24 | | 2,967.68 | |
|  | GeneScan | 52,478 | 26,579.64 | 1,232.16 | 7.6 | 162.1 | | 3,839.84 | |
| Homology | *Bos taurus* | 20,290 | 22,768.49 | 1,512.95 | 8.55 | 177.05 | | 2,816.97 | |
|  | *Camelus bactrianus* | 21,002 | 24,277.54 | 1,516.61 | 8.71 | 174.22 | | 2,954.04 | |
|  | *Camelus dromedarius* | 21,082 | 24,355.31 | 1,511.41 | 8.7 | 173.63 | | 2,964.85 | |
|  | *Equus caballus* | 20,795 | 22,119.75 | 1,438.35 | 8.23 | 174.82 | | 2,861.46 | |
|  | *Homo sapiens* | 20,034 | 24,905.10 | 1,557.88 | 8.79 | 177.33 | | 2,998.86 | |
|  | *Mus musculus* | 20,042 | 24,077.58 | 1,526.39 | 8.55 | 178.46 | | 2,985.67 | |
|  | *Vicugna pacos* | 20,483 | 24,998.88 | 1,524.88 | 8.82 | 172.82 | | 3,000.37 | |
| RNA-seq | | 119,824 | 539,292 | 5,354.51 | 618.18 | | 278.84 | | 3,891.91 |
| Glean | | 21,435 | 21,460 | 24,063.78 | 1,520.17 | | 183.91 | | 3,102.62 |

**Table S8. Sampling details.**

| Sample name | Species | Subspecies/  variety | Genome | Fleece color | Sex* | Tissue | Location | |
| --- | --- | --- | --- | --- | --- | --- | --- | --- |
|  |  |  |  |  |  |  | Latitude | Longitude |
| Guanaco | | | | | | | | |
| 138DA | *Lama guanicoe* | *cacsilensis* | *de novo* | wild type | male | Blood, muscle & biopsy tissue | -18.19 | -69.54 |
| 8 | *Lama guanicoe* | *cacsilensis* | Resequence | wild type | male | Blood | -18.21 | -69.56 |
| 22 | *Lama guanicoe* | *cacsilencis* | Resequence | wild type | female | Blood | -25.02 | -70.43 |
| 24 | *Lama guanicoe* | *cacsilencis* | Resequence | wild type | * | Blood | -30.36 | -72.37 |
| 27 | *Lama guanicoe* | *guanicoe* | Resequence | wild type | * | Muscle | -33.39 | -71.38 |
| 28 | *Lama guanicoe* | *guanicoe* | Resequence | wild type | female | Blood | -37.35 | -69.43 |
| 31 | *Lama guanicoe* | *guanicoe* | Resequence | wild type | * | Blood | -54.37 | -71.36 |
| 32 | *Lama guanicoe* | *guanicoe* | Resequence | wild type | male | Liver | -48.40 | -73.39 |
| Vicuña | | | | | | | | |
| 23CA | *Vicugna vicugna* | *mensalis* | *de Novo* | wild type | female | Blood, muscle & biopsy tissue | -18.17 | -69.43 |
| 18 | *Vicugna vicugna* | *mensalis* | Resequence | wild type | * | Blood | -18.17 | -69.43 |
| 20 | *Vicugna vicugna* | *mensalis* | Resequence | wild type | * | Blood | -18.87 | -69.09 |
| 33 | *Vicugna vicugna* | *mensalis* | Resequence | wild type | * | Blood | -13.70 | -75.25 |
| 37 | *Vicugna vicugna* | *vicugna* | Resequence | wild type | * | Blood | -24.75 | -68.65 |
| 38 | *Vicugna vicugna* | *vicugna* | Resequence | wild type | female | Blood | -28.67 | -67.58 |
| 39 | *Vicugna vicugna* | *vicugna* | Resequence | wild type | female | Muscle | -27.09 | -68.93 |
| 40 | *Vicugna vicugna* | *vicugna* | Resequence | wild type | male | Blood | -22.12 | -65.86 |
| Llama | | | | | | | | |
| 15A | *Lama glama* | chaku | *de Novo* | dark brown and white | male | skin | -18.19 | -69.56 |
| 14A | *Lama glama* | cara | Resequence | white | male | skin | -18.19 | -69.56 |
| 17A | *Lama glama* | chaku | Resequence | light brown | female | skin | -18.19 | -69.56 |
| 16 | *Lama glama* | *chaku* | Resequence | white | female | Blood | -38.1 | -72.55 |
| 17 | *Lama glama* | *chaku* | Resequence | beige | male | Blood | -38.1 | -72.55 |
| 19 | *Lama glama* | *chaku* | Resequence | brown | male | Blood | -38.1 | -72.55 |
| 20 | *Lama glama* | *chaku* | Resequence | light brown | male | Blood | -38.1 | -72.55 |
| 21 | *Lama glama* | *chaku* | Resequence | brown | male | Blood | -38.1 | -72.55 |
| Alpaca | | | | | | | | |
| 1 | *Vicugna pacos* | *huacaya* | Resequence | white | male | Blood | -38.1 | -72.55 |
| 4 | *Vicugna pacos* | *suri* | Resequence | white | male | Blood | -38.1 | -72.55 |
| 7 | *Vicugna pacos* | *huacaya* | Resequence | white | female | Blood | -38.1 | -72.55 |
| 9 | *Vicugna pacos* | *huacaya* | Resequence | light brown | female | Blood | -38.1 | -72.55 |
| 10 | *Vicugna pacos* | *huacaya* | Resequence | dark brown | male | Blood | -38.1 | -72.55 |
| 11 | *Vicugna pacos* | *huacaya* | Resequence | dark brown | female | Blood | -38.1 | -72.55 |
| 13 | *Vicugna pacos* | *suri* | Resequence | black | female | Blood | -38.1 | -72.55 |
| 17 Alpacas | | | | | | | | |
| 1 | *Vicugna pacos* | *huacaya* | Resequence | Black | male | Skin | 37.73 | 112.73 |
| 2 | *Vicugna pacos* | *huacaya* | Resequence | Brown | male | Skin | 37.73 | 112.73 |
| 3 | *Vicugna pacos* | *huacaya* | Resequence | Black | female | Skin | 37.73 | 112.73 |
| 4 | *Vicugna pacos* | *huacaya* | Resequence | Brown | female | Skin | 37.73 | 112.73 |
| 5 | *Vicugna pacos* | *huacaya* | Resequence | Brown | female | Skin | 37.73 | 112.73 |
| 6 | *Vicugna pacos* | *huacaya* | Resequence | White | male | Skin | 37.73 | 112.73 |
| 7 | *Vicugna pacos* | *huacaya* | Resequence | White | female | Skin | 37.73 | 112.73 |
| 8 | *Vicugna pacos* | *huacaya* | Resequence | White | female | Skin | 37.73 | 112.73 |
| 9 | *Vicugna pacos* | *huacaya* | Resequence | Brown | male | Skin | 37.73 | 112.73 |
| 10 | *Vicugna pacos* | *huacaya* | Resequence | White | female | Skin | 37.73 | 112.73 |
| 11 | *Vicugna pacos* | *huacaya* | Resequence | White | female | Skin | 37.73 | 112.73 |
| 12 | *Vicugna pacos* | *huacaya* | Resequence | White | female | Skin | 37.73 | 112.73 |
| 13 | *Vicugna pacos* | *Suri* | Resequence | Brown | male | Skin | 37.73 | 112.73 |
| 14 | *Vicugna pacos* | *Suri* | Resequence | White | male | Skin | 37.73 | 112.73 |
| 15 | *Vicugna pacos* | *Suri* | Resequence | White | male | Skin | 37.73 | 112.73 |
| 16 | *Vicugna pacos* | *Suri* | Resequence | White | male | Skin | 37.73 | 112.73 |
| 17 | *Vicugna pacos* | *Suri* | Resequence | White | male | Skin | 37.73 | 112.73 |

* inferred from sequence data

**Table S9**. **Resequencing data summary**

| **Species** | **Sample ID** | **Reads (x10^6^)** | **Bases (Gb)** | **Q30 rate (%)** | **Depth** |
| --- | --- | --- | --- | --- | --- |
| ***L. guanicoe*** | 31 | 284.59 | 42.7 | 95.4 | 20.43 |
|  | 28 | 261.94 | 39.29 | 95.34 | 18.80 |
|  | 8 | 237.62 | 35.65 | 93.89 | 17.06 |
|  | 27 | 212.53 | 31.88 | 94.77 | 15.25 |
|  | 24 | 256.07 | 38.41 | 95.32 | 18.38 |
|  | 32 | 267.14 | 40.07 | 95.4 | 19.17 |
|  | 22 | 250.77 | 37.62 | 95.41 | 18.00 |
| ***V. pacos*** | 7 | 254.25 | 38.14 | 95.28 | 18.79 |
|  | 9 | 254.2 | 38.13 | 95.19 | 18.78 |
|  | 1 | 227.35 | 34.1 | 95.59 | 16.80 |
|  | 13 | 255.43 | 38.32 | 95.36 | 18.88 |
|  | 4 | 235.81 | 35.37 | 95.2 | 17.42 |
|  | 10 | 275.35 | 41.3 | 95.3 | 20.34 |
|  | 11 | 259.42 | 38.92 | 95.12 | 19.17 |
| ***L. glama*** | 17 | 256.89 | 38.53 | 94.95 | 18.70 |
|  | 17A | 223.29 | 33.5 | 94.35 | 16.26 |
|  | 19 | 275.46 | 41.32 | 95.18 | 20.06 |
|  | 16 | 246.79 | 37.01 | 95.23 | 17.97 |
|  | 21 | 250.21 | 37.53 | 95.25 | 18.22 |
|  | 20 | 266.24 | 39.93 | 95.16 | 19.38 |
|  | 14A | 303.75 | 45.57 | 94.5 | 22.12 |
| ***V. vicugna*** | 40 | 277.13 | 41.57 | 95.49 | 20.28 |
|  | 38 | 299.96 | 44.99 | 95.7 | 21.95 |
|  | 18 | 221.68 | 33.25 | 94.52 | 16.22 |
|  | 39 | 284.63 | 42.69 | 95.71 | 20.82 |
|  | 33 | 252.57 | 37.88 | 95.47 | 18.48 |
|  | 37 | 248.04 | 37.2 | 95.64 | 18.15 |
|  | 20 | 222.39 | 33.36 | 94.54 | 16.27 |

*Q*, is defined as *Q* = -10log_10_(*e*), where *e* is the estimated probability of an incorrect base call.  A quality score of 30 (*Q*30) represents an error rate of 1 in 1000, with a corresponding call accuracy of 99.9%.

**Table S10. ABBA-BABA statistics.**

| *P*_1_ | *P*_2_ | *P*_3_ | *O* | \| *f*_d_ \| | *D*_statistics_ | *sd* |
| --- | --- | --- | --- | --- | --- | --- |
| Alpaca | Vicuña | Llama | Bactrian | 0.389 | 0.565 | 0.006 |
| Alpaca | Vicuña | GL* | Bactrian | 0.324 | 0.55 | 0.007 |
| Alpaca | Vicuña | Guanaco | Bactrian | 0.265 | 0.539 | 0.006 |
| Guanaco | Llama | Alpaca | Bactrian | 0.133 | -0.265 | 0.004 |
| Guanaco | Llama | VA* | Bactrian | 0.089 | -0.231 | 0.005 |
| Guanaco | Llama | Vicuña | Bactrian | 0.045 | -0.185 | 0.006 |

Bactrian camel was used as the outgroup. *GL = Guanaco/Ilama lineage, *VA =Vicuña/Alpaca lineage

**Table S11. Introgressed segments from llama into alpaca by using f_d_ and LAI (Top1%, 100kb windows)**

| Chromosome_start-end | Gene | *f_d_* | LAI | Methods | |
| --- | --- | --- | --- | --- | --- |
| 3_60037430-60137429 | *Cnot6l* | 0.881 | 0.75 | Overlap | |
| 3_60237430-60337429 | *MRPL1* | 0.837 | 0.75 | Overlap | |
| 3_61037430-61137429 | *PAQR3* | 0.836 | 0.375 | *f****_d_*** | |
| 3_61337430-61437429 | *NAA11* | 0.651 | 0.89 | LAI | |
| 3_61437430-61537429 | *GK2* | 0.766 | 1 | LAI | |
| 3_61437430-61537429 | *Unknown* | 0.766 | 1 | LAI | |
| 3_61837430-61937429 | *ANTXR2* | 0.763 | 0.876 | LAI | |
| 3_61937430-62037429 | *Unknown* | 0.812 | 0.988 | LAI | |
| 3_62037430-62137429 | *PRDM8* | 0.847 | 1 | Overlap | |
| 3_62037430-62137429 | *FGF5* | 0.847 | 1 | Overlap | |
| 3_62137430-62237429 | *NA* | 0.916 | 1 | Overlap | |
| 3_62737430-62837429 | *PRKG2* | 0.871 | 0.625 | *f****_d_*** | |
| 11_43788735-43888734 | *SKP2* | 0.752 | 0.709 | LAI | |
| 11_43788735-43888734 | *NADK2* | 0.752 | 0.709 | LAI | |
| 11_43788735-43888734 | *RANBP3L* | 0.752 | 0.709 | LAI | |
| 11_44088735-44188734 | *SLC1A3* | 0.871 | 0.733 | Overlap | |
| chrUN_33181013-33281012 | *Celsr1* | 0.863 | 0.5 | *f****_d_*** | |
| chrUN_33181013-33281012 | *GRAMD4* | 0.863 | 0.5 | *f****_d_*** | |
| chrUN_33281013-33381012 | *CERK* | 0.903 | 0.5 | *f****_d_*** | |
| chrUN_33281013-33381012 | *TBC1D22A* | 0.903 | 0.5 | *f****_d_*** | |
| chrUN_41881013-41981012 | *FZD3* | 0.84 | 0.701 | Overlap | |
| chrUN_41881013-41981012 | *Fbxo16* | 0.84 | 0.701 | Overlap | |
| 10_20917711-21017710 | *IGFALS* | 0.847 | 0.683 | Overlap | |
| 10_20917711-21017710 | *NUS1* | 0.847 | 0.683 | Overlap | |
| 16_100001-200000 | *FASN* | 0.909 | 0.537 | *f****_d_*** | |
| 16_200001-300000 | *Dus1l* | 0.894 | 0.625 | *f****_d_*** | |
| 16_200001-300000 | *Gps1* | 0.894 | 0.625 | *f****_d_*** | |
| 16_200001-300000 | *Rfng* | 0.894 | 0.625 | *f****_d_*** | |
| 16_200001-300000 | *Cbr2* | 0.894 | 0.625 | *f****_d_*** | |
| 16_200001-300000 | *DCXR* | 0.894 | 0.625 | *f****_d_*** | |
| 16_200001-300000 | *Rac3* | 0.894 | 0.625 | *f****_d_*** | |
| 16_200001-300000 | *LRRC45* | 0.894 | 0.625 | *f****_d_*** | |
| 16_200001-300000 | *CENPX* | 0.894 | 0.625 | *f****_d_*** | |
| 16_200001-300000 | *Aspscr1* | 0.894 | 0.625 | *f****_d_*** | |
| 16_200001-300000 | *NOTUM* | 0.894 | 0.625 | *f****_d_*** | |
| 16_300001-400000 | *MYADML2* | 0.861 | 0.625 | *f****_d_*** | |
| 16_300001-400000 | *PYCR1* | 0.861 | 0.625 | *f****_d_*** | |
| 16_300001-400000 | *Mafg* | 0.861 | 0.625 | *f****_d_*** | |
| 16_300001-400000 | *SIRT7* | 0.861 | 0.625 | *f****_d_*** | |
| 16_300001-400000 | *PCYT2* | 0.861 | 0.625 | *f****_d_*** | |
| 16_300001-400000 | *NPB* | 0.861 | 0.625 | *f****_d_*** | |
| 16_300001-400000 | *ALYREF* | 0.861 | 0.625 | *f****_d_*** | |
| 16_300001-400000 | *ARHGDIA* | 0.861 | 0.625 | *f****_d_*** | |
| 16_300001-400000 | *P4HB* | 0.861 | 0.625 | *f****_d_*** | |
| 16_300001-400000 | *MCRIP1* | 0.861 | 0.625 | *f****_d_*** | |
| 16_400001-500000 | *GCGR* | 0.889 | 0.625 | *f****_d_*** | |
| 16_400001-500000 | *SLC25A10* | 0.889 | 0.625 | *f****_d_*** | |
| 16_400001-500000 | *MRPL12* | 0.889 | 0.625 | *f****_d_*** | |
| 16_400001-500000 | *HGS* | 0.889 | 0.625 | *f****_d_*** | |
| 16_400001-500000 | *HGS* | 0.889 | 0.625 | *f****_d_*** | |
| 16_400001-500000 | *ARL16* | 0.889 | 0.625 | *f****_d_*** | |
| 16_400001-500000 | *CCDC137* | 0.889 | 0.625 | *f****_d_*** | |
| 16_400001-500000 | *OXLD1* | 0.889 | 0.625 | *f****_d_*** | |
| 16_400001-500000 | *PDE6G* | 0.889 | 0.625 | *f****_d_*** | |
| 16_400001-500000 | *TSPAN10* | 0.889 | 0.625 | *f****_d_*** | |
| 16_400001-500000 | *NPLOC4* | 0.889 | 0.625 | *f****_d_*** | |
| 16_500001-600000 | *Unknown* | 0.871 | 0.625 | *f****_d_*** | |
| 16_500001-600000 | *FAAP100* | 0.871 | 0.625 | *f****_d_*** | |
| 16_500001-600000 | *FSCN2* | 0.871 | 0.625 | *f****_d_*** | |
| 16_500001-600000 | *actg1* | 0.871 | 0.625 | *f****_d_*** | |
| 16_600001-700000 | *BAHCC1* | 0.9 | 0.625 | *f****_d_*** | |
| 16_700001-800000 | *SLC38A10* | 0.877 | 0.625 | *f****_d_*** | |
| 16_700001-800000 | *SLC38A10* | 0.877 | 0.625 | *f****_d_*** | |
| 16_700001-800000 | *Ndufaf8* | 0.877 | 0.625 | *f****_d_*** | |
| 16_700001-800000 | *Tepsin* | 0.877 | 0.625 | *f****_d_*** | |
| 16_700001-800000 | *CEP131* | 0.877 | 0.625 | *f****_d_*** | |
| 16_900001-1000000 | *CHMP6* | 0.874 | 0.5 | *f****_d_*** | |
| 16_900001-1000000 | *Rptor* | 0.874 | 0.5 | *f****_d_*** | |
| 9_21852453-21952452 | *AKNAD1* | 0.843 | 0.625 | *f****_d_*** | |
| 9_21852453-21952452 | *RPL9P9* | 0.843 | 0.625 | *f****_d_*** | |
| 9_21852453-21952452 | *GPSM2* | 0.843 | 0.625 | *f****_d_*** | |
| 9_23052453-23152452 | *RBM15* | 0.868 | 0.625 | *f****_d_*** | |
| 9_23052453-23152452 | *SLC16A4* | 0.868 | 0.625 | *f****_d_*** | |
| 2_96476766-96576765 | *C4orf33* | 0.875 | 0.5 | *f****_d_*** | |
| 24_6013996-6113995 | *TRAPPC8* | 0.853 | 0.401 | *f****_d_*** | |
| chrUN_94880520-94980519 | *EXOC4* | 0.756 | 0.748 | LAI | |
| chrUN_95380520-95480519 | *EXOC4* | 0.844 | 0.625 | *f****_d_*** | |
| chrUN_95780520-95880519 | *CHCHD3* | 0.846 | 0.625 | *f****_d_*** | |
| chrUN_101580520-101680519 | *POT1* | 0.596 | 0.838 | LAI | |
| chrUN_101680520-101780519 | *GPR37* | 0.786 | 0.75 | LAI | |
| 28_7489923-7589922 | *MRPS9* | 0.754 | 0.676 | LAI | |
| 28_7489923-7589922 | *GPR45* | 0.754 | 0.676 | LAI | |
| 24_18338448-18438447 | *Unknown* | 0.899 | 0.625 | *f****_d_*** | |
| 24_18338448-18438447 | *PDGFA* | 0.899 | 0.625 | *f****_d_*** | |
| 24_18338448-18438447 | *Unknown* | 0.899 | 0.625 | *f****_d_*** | |
| 24_18438448-18538447 | *FAM210A* | 0.959 | 0.625 | *f****_d_*** | |
| 24_18438448-18538447 | *MC5R* | 0.959 | 0.625 | *f****_d_*** | |
| 24_18538448-18634228 | *MC2R* | 0.938 | 0.405 | *f****_d_*** | |
| 24_18538448-18634228 | *Paip1* | 0.938 | 0.405 | *f****_d_*** | |
| 24_18538448-18634228 | *FRG1* | 0.938 | 0.405 | *f****_d_*** | |
| chrUN_114938671-115038670 | *NTRK2* | 0.841 | 0.645 | *f****_d_*** | |
| 6_60387980-60487979 | *GBP4* | 1.84 | 0 | *f****_d_*** | |
| 26_9427099-9527098 | *TRIML2* | 0.838 | 0.786 | Overlap | |
| 26_9427099-9527098 | *ZFP42* | 0.838 | 0.786 | Overlap | |
| 26_17093358-17193357 | *IKBKB* | 0.806 | 0.731 | LAI | |
| 11_62117369-62217368 | *C1orf131* | 0.838 | 0.383 | *f****_d_*** | |
| 11_62117369-62217368 | *GNPAT* | 0.838 | 0.383 | *f****_d_*** | |
| 11_62117369-62217368 | *EXOC8* | 0.838 | 0.383 | *f****_d_*** | |
| 11_62117369-62217368 | *SPRTN* | 0.838 | 0.383 | *f****_d_*** | |
| 11_62117369-62217368 | *EGLN1* | 0.838 | 0.383 | *f****_d_*** | |
| 7_28797379-28897378 | *Unknown* | 0.838 | 0.455 | *f****_d_*** | |
| 7_28797379-28897378 | *YAE1D1* | 0.838 | 0.455 | *f****_d_*** | |
| 21_23015279-23115278 | *MAP1LC3B* | 0.906 | 0.5 | *f****_d_*** | |
| 21_23015279-23115278 | *ZCCHC14* | 0.906 | 0.5 | *f****_d_*** | |
| 16_31831517-31931516 | *SMG8* | 0.823 | 0.741 | LAI | |
| 16_31831517-31931516 | *PRR11* | 0.823 | 0.741 | LAI | |
| 16_31831517-31931516 | *TRIM37* | 0.823 | 0.741 | LAI | |
| 16_32031517-32131516 | *PPM1E* | 0.877 | 0.781 | Overlap | |
| 16_32131517-32231516 | *PPM1E* | 0.899 | 0.75 | Overlap | |
| 16_32131517-32231516 | *RAD51C* | 0.899 | 0.75 | Overlap | |
| 16_32231517-32331516 | *TEX14* | 0.872 | 0.554 | *f****_d_*** | |
| chrUN_230937540-231037539 | *RPL29* | 0.845 | 0.558 | *f****_d_*** | |
| chrUN_230937540-231037539 | *EPCAM* | 0.845 | 0.558 | *f****_d_*** | |
| chrUN_230937540-231037539 | *MSH2* | 0.845 | 0.558 | *f****_d_*** | |
| 30_14907467-14916093 | *MSH6* | 0.811 | 0.71 | LAI | |
| 30_14907467-14916093 | *Fbxo11* | 0.811 | 0.71 | LAI | |
| 26_22408004-22508003 | *MCPH1* | 0.8 | 0.688 | LAI | |
| 26_22708004-22808003 | *AGPAT5* | 0.736 | 0.748 | LAI | |
| 26_22708004-22808003 | *XKR5* | 0.736 | 0.748 | LAI | |
| 23_28500866-28600865 | *IKBKE* | 0.811 | 0.823 | LAI | |
| 23_28600866-28700865 | *Rassf5* | 0.802 | 0.875 | LAI | |
| chrUN_249057006-249157005 | *Unknown* | 0.849 | 0.375 | *f****_d_*** | |
| chrUN_249057006-249157005 | *RASA3* | 0.849 | 0.375 | *f****_d_*** | |
| chrUN_251230484-251330483 | *APBA2* | 0.76 | 0.709 | LAI | |
| chrUN_251230484-251330483 | *FAM189A1* | 0.76 | 0.709 | LAI | |
| chrUN_258330484-258430483 | *Ints1* | 0.842 | 0.435 | *f****_d_*** | |
| chrUN_258330484-258430483 | *MICALL2* | 0.842 | 0.435 | *f****_d_*** | |
| 26_24876188-24976187 | *Csmd1* | 0.742 | 0.75 | LAI | |
| 26_24876188-24976187 | *Unknown* | 0.742 | 0.75 | LAI | |
| chrUN_287913310-288013309 | *GABRB3* | 0.791 | 0.722 | LAI | |
| chrUN_288013310-288113309 | *GABRA5* | 0.832 | 0.838 | LAI | |
| 26_26058629-26104728 | *ZNF596* | 0.84 | 0.375 | *f****_d_*** | |
| 26_26104729-26204728 | *TDRP* | 0.898 | 0.75 | Overlap | |
| 26_26104729-26204728 | *ERICH1* | 0.898 | 0.75 | Overlap | |
| 26_26204729-26304728 | *Unknown* | 0.908 | 0.75 | Overlap | |
| 26_26304729-26404728 | *Unknown* | 0.905 | 0.75 | Overlap | |
| chrUN_309170225-309270224 | *OR51B6* | 0.723 | 0.962 | LAI | |
| chrUN_309170225-309270224 | *OR51B5* | 0.723 | 0.962 | LAI | |
| chrUN_309170225-309270224 | *OR51B5* | 0.723 | 0.962 | LAI | |
| chrUN_309170225-309270224 | *OR51B2* | 0.723 | 0.962 | LAI | |
| chrUN_309170225-309270224 | *HBE1* | 0.723 | 0.962 | LAI | |
| chrUN_309170225-309270224 | *HBE1* | 0.723 | 0.962 | LAI | |
| chrUN_309170225-309270224 | *HBB* | 0.723 | 0.962 | LAI | |
| chrUN_309270225-309370224 | *OR52Z1* | 0.859 | 1 | Overlap | |
| chrUN_309270225-309370224 | *OR52A1* | 0.859 | 1 | Overlap | |
| chrUN_309270225-309370224 | *OR52A5* | 0.859 | 1 | Overlap | |
| chrUN_309270225-309370224 | *OR52E2* | 0.859 | 1 | Overlap | |
| chrUN_309270225-309370224 | *OR51V1* | 0.859 | 1 | Overlap | |
| chrUN_309270225-309370224 | *OR52K2* | 0.859 | 1 | Overlap | |
| chrUN_309270225-309370224 | *OR52D1* | 0.859 | 1 | Overlap | |
| chrUN_309270225-309370224 | *OR51L1* | 0.859 | 1 | Overlap | |
| chrUN_309370225-309470224 | *OR51L1* | 0.698 | 1 | LAI | |
| chrUN_309370225-309470224 | *OR51F2* | 0.698 | 1 | LAI | |
| chrUN_309370225-309470224 | *OR52A1* | 0.698 | 1 | LAI | |
| chrUN_309370225-309470224 | *OR52D1* | 0.698 | 1 | LAI | |
| chrUN_309370225-309470224 | *OR52P1P* | 0.698 | 1 | LAI | |
| chrUN_309370225-309470224 | *OR52A1* | 0.698 | 1 | LAI | |
| chrUN_309370225-309470224 | *OR52A1* | 0.698 | 1 | LAI | |
| chrUN_309470225-309570224 | *OR52A1* | 0.614 | 1 | LAI | |
| chrUN_309470225-309570224 | *OR52D1* | 0.614 | 1 | LAI | |
| chrUN_309470225-309570224 | *OR52E4* | 0.614 | 1 | LAI | |
| chrUN_309470225-309570224 | *OR52E1* | 0.614 | 1 | LAI | |
| chrUN_309470225-309570224 | *OR52J3* | 0.614 | 1 | LAI | |
| chrUN_309470225-309570224 | *OR52E2* | 0.614 | 1 | LAI | |
| chrUN_309470225-309570224 | *OR52D1* | 0.614 | 1 | LAI | |
| chrUN_309470225-309570224 | *OR52E1* | 0.614 | 1 | LAI | |
| chrUN_309570225-309670224 | *OR52E2* | 0.661 | 0.987 | LAI | |
| chrUN_309570225-309670224 | *OR52E2* | 0.661 | 0.987 | LAI | |
| chrUN_309570225-309670224 | *OR52J3* | 0.661 | 0.987 | LAI | |
| chrUN_309570225-309670224 | *OR52J3* | 0.661 | 0.987 | LAI | |
| chrUN_309570225-309670224 | *OR51A7* | 0.661 | 0.987 | LAI | |
| chrUN_309570225-309670224 | *OR51G2* | 0.661 | 0.987 | LAI | |
| chrUN_309570225-309670224 | *OR51L1* | 0.661 | 0.987 | LAI | |
| chrUN_309570225-309670224 | *MMP26* | 0.661 | 0.987 | LAI | |
| chrUN_309670225-309770224 | *OR51A4* | 0.724 | 0.859 | LAI | |
| chrUN_309670225-309770224 | *OR51G1* | 0.724 | 0.859 | LAI | |
| chrUN_309670225-309770224 | *OR51G2* | 0.724 | 0.859 | LAI | |
| chrUN_309670225-309770224 | *OR51A7* | 0.724 | 0.859 | LAI | |
| chrUN_309670225-309770224 | *OR51A7* | 0.724 | 0.859 | LAI | |
| chrUN_309670225-309770224 | *OR51T1* | 0.724 | 0.859 | LAI | |
| chrUN_309670225-309770224 | *OR51L1* | 0.724 | 0.859 | LAI | |
| chrUN_309670225-309770224 | *OR51H1* | 0.724 | 0.859 | LAI | |
| chrUN_309670225-309770224 | *OR51H1* | 0.724 | 0.859 | LAI | |
| chrUN_309670225-309770224 | *OR51S1* | 0.724 | 0.859 | LAI | |
| chrUN_309770225-309870224 | *OR51S1* | 0.809 | 1 | LAI | |
| chrUN_309770225-309870224 | *OR51F2* | 0.809 | 1 | LAI | |
| chrUN_309770225-309870224 | *OR52R1* | 0.809 | 1 | LAI | |
| chrUN_309770225-309870224 | *OR51F1* | 0.809 | 1 | LAI | |
| chrUN_309770225-309870224 | *OR51F1* | 0.809 | 1 | LAI | |
| chrUN_309770225-309870224 | *OR51F2* | 0.809 | 1 | LAI | |
| chrUN_309770225-309870224 | *OR51F2* | 0.809 | 1 | LAI | |
| chrUN_309770225-309870224 | *OR51F2* | 0.809 | 1 | LAI | |
| chrUN_309870225-309970224 | *OR51F2* | 0.941 | 1 | Overlap | |
| chrUN_309870225-309970224 | *Or51e2* | 0.941 | 1 | Overlap | |
| chrUN_309870225-309970224 | *OR51I2* | 0.941 | 1 | Overlap | |
| chrUN_309870225-309970224 | *OR51I2* | 0.941 | 1 | Overlap | |
| chrUN_309870225-309970224 | *OR51E1* | 0.941 | 1 | Overlap | |
| chrUN_309870225-309970224 | *OR51D1* | 0.941 | 1 | Overlap | |
| chrUN_309870225-309970224 | *TRIM68* | 0.941 | 1 | Overlap | |
| chrUN_309870225-309970224 | *OR52I2* | 0.941 | 1 | Overlap | |
| chrUN_309870225-309970224 | *SSU72* | 0.941 | 1 | Overlap | |
| chrUN_309870225-309970224 | *OR52B4* | 0.941 | 1 | Overlap | |
| chrUN_316370225-316470224 | *KPNA2* | 0.827 | 0.734 | LAI | |
| chrUN_316370225-316470224 | *BLMH* | 0.827 | 0.734 | LAI | |
| chrUN_316370225-316470224 | *BLMH* | 0.827 | 0.734 | LAI | |
| chrUN_316370225-316470224 | *HACD1* | 0.827 | 0.734 | LAI | |
| chrUN_318277679-318370224 | *PHKA1* | 0.851 | 0 | *f****_d_*** | |
| chrUN_339170225-339270224 | *Unknown* | 0.858 | 0.479 | *f****_d_*** | |
| 5_39100001-39200000 | *EVX2* | 0.869 | 0.612 | *f****_d_*** | |
| 5_39100001-39200000 | *LNPK* | 0.869 | 0.612 | *f****_d_*** | |
| 5_39700001-39800000 | *Atp5mc3* | 0.755 | 0.75 | LAI | |
| 5_39800001-39900000 | *ATF2* | 0.87 | 0.75 | Overlap | |
| 5_39900001-40000000 | *CHN1* | 0.801 | 0.696 | LAI | |
| 5_40000001-40100000 | *CHRNA1* | 0.932 | 0.625 | *f****_d_*** | |
| 5_40200001-40300000 | *Wipf1* | 0.856 | 0.636 | *f****_d_*** | |
| 5_40300001-40400000 | *GPR155* | 0.758 | 0.75 | LAI | |
| 5_40300001-40400000 | *SCRN3* | 0.758 | 0.75 | LAI | |
| 5_40300001-40400000 | *CIR1* | 0.758 | 0.75 | LAI | |
| 5_40400001-40500000 | *SP9* | 0.904 | 0.75 | Overlap | |
| 5_40400001-40500000 | *OLA1* | 0.904 | 0.75 | Overlap | |
| 5_40500001-40600000 | *OLA1* | 0.873 | 0.75 | Overlap | |
| 5_40600001-40700000 | *Unknown* | 0.875 | 0.75 | Overlap | |
| 5_40700001-40800000 | *Sp3* | 0.877 | 0.75 | Overlap | |
| 12_14315433-14415432 | *Tmbim4* | 0.806 | 0.675 | LAI | |
| 12_34215433-34315432 | *SLC6A15* | 0.782 | 0.714 | LAI | |
| 6_8472824-8572823 | *BRMS1L* | 0.874 | 0.125 | *f****_d_*** | |
| 6_21972824-22072823 | *CGRRF1* | 0.859 | 0.625 | *f****_d_*** | |
| 25_503612-603611 | *MTERF3* | 0.879 | 0.633 | *f****_d_*** | |
| 25_503612-603611 | *PTDSS1* | 0.879 | 0.633 | *f****_d_*** | |
| 25_26603612-26703611 | *PHF20L1* | 0.854 | 0.62 | *f****_d_*** | |
| 25_26603612-26703611 | *TG* | 0.854 | 0.62 | *f****_d_*** | |
| 25_28203612-28303611 | *KHDRBS3* | 0.734 | 0.737 | LAI | |
| 25_28403612-28503611 | *Unknown* | 0.89 | 0.875 | Overlap | |
| 25_30603612-30703611 | *KCNK9* | 0.837 | 0.5 | *f****_d_*** | |
| 25_30603612-30703611 | *TRAPPC9* | 0.837 | 0.5 | *f****_d_*** | |
| 14_2744126-2844125 | *TRIM13* | 0.84 | 0.625 | *f****_d_*** | |
| 14_2744126-2844125 | *KCNRG* | 0.84 | 0.625 | *f****_d_*** | |
| 14_4544126-4644125 | *TPTE2* | 0.738 | 0.672 | LAI | |
| 14_4544126-4644125 | *SLC25A15* | 0.738 | 0.672 | LAI | |
| 14_4644126-4744125 | *MRPS31* | 0.819 | 0.727 | LAI | |
| 14_4644126-4744125 | *FOXO1* | 0.819 | 0.727 | LAI | |
| 14_4744126-4844125 | *FOXO1* | 0.819 | 0.75 | LAI | |
| 8_22100001-22200000 | *PRKN* | 0.858 | 0.537 | *f****_d_*** | |
| 23_6211217-6311216 | *Ptgs2* | 0.807 | 0.736 | LAI | |
| 23_23011217-23111216 | *RGS1* | 0.775 | 0.875 | LAI | |
| 23_23011217-23111216 | *RGS13* | 0.775 | 0.875 | LAI | |
| 23_23111217-23211216 | *Rgs2* | 0.721 | 0.851 | LAI | |
| 20_18872385-18972384 | *SUPT3H* | 0.794 | 0.719 | LAI | |
| 20_23172385-23272384 | *PKHD1* | 0.855 | 0.535 | *f****_d_*** | |
| 7_1300001-1400000 | *CBLL1* | 0.847 | 0.875 | Overlap | |
| 7_1300001-1400000 | *SLC26A4* | 0.847 | 0.875 | Overlap | |
| 7_1400001-1500000 | *DUS4L* | 0.895 | 0.638 | *f****_d_*** | |
| 7_1400001-1500000 | *COG5* | 0.895 | 0.638 | *f****_d_*** | |
| 7_5500001-5600000 | *Unknown* | 0.871 | 0.375 | *f****_d_*** | |
| 7_5500001-5600000 | *PHTF2* | 0.871 | 0.375 | *f****_d_*** | |
| 7_5500001-5600000 | *IFITM1* | 0.871 | 0.375 | *f****_d_*** | |
| 7_5700001-5800000 | *Magi2* | 0.875 | 0.375 | *f****_d_*** | |
| 7_7700001-7800000 | *CD36* | 0.877 | 0.497 | *f****_d_*** | |
| 7_8600001-8700000 | *HGF* | 0.843 | 0.729 | Overlap | |
| 7_8800001-8900000 | *CACNA2D1* | 0.734 | 0.737 | LAI | |
| 7_8900001-9000000 | *SRP14* | 0.816 | 0.75 | LAI | |
| 2_41451425-41551424 | *NFKB1* | 0.857 | 0.736 | Overlap | |
| 27_20953853-21053852 | *Unknown* | 0.867 | 0.538 | *f****_d_*** | |
| 15_13059970-13159969 | *Unknown* | 0.844 | 0.589 | *f****_d_*** | |
| 19_12125846-12225845 | *NCOA3* | 0.853 | 0.402 | *f****_d_*** | |
| 19_12125846-12225845 | *SULF2* | 0.853 | 0.402 | *f****_d_*** | |
| 13_30341542-30441541 | *Casz1* | 0.866 | 0.583 | *f****_d_*** | |
| 13_33741542-33841541 | *RSC1A1* | 0.878 | 0.526 | *f****_d_*** | |
| 13_33741542-33841541 | *PLEKHM2* | 0.878 | 0.526 | *f****_d_*** | |
| 13_33841542-33941541 | *SLC25A34* | 0.913 | 0.583 | *f****_d_*** | |
| 13_33841542-33941541 | *TMEM82* | 0.913 | 0.583 | *f****_d_*** | |
| 13_33841542-33941541 | *FBLIM1* | 0.913 | 0.583 | *f****_d_*** | |
| 13_33841542-33941541 | *Unknown* | 0.913 | 0.583 | *f****_d_*** | |
| 13_33941542-34041541 | *SPEN* | 0.896 | 0.625 | *f****_d_*** | |
| 13_33941542-34041541 | *Zbtb17* | 0.896 | 0.625 | *f****_d_*** | |
| 13_33941542-34041541 | *SRARP* | 0.896 | 0.625 | *f****_d_*** | |
| 11_25833452-25933451 | *BICC1* | 0.854 | 0.473 | *f****_d_*** | |
| 11_25833452-25933451 | *TFAM* | 0.854 | 0.473 | *f****_d_*** | |
| 20_35399569-35499568 | *Gfod1* | 0.869 | 0.729 | Overlap | |
| 20_35499569-35599568 | *TBC1D7* | 0.89 | 0.75 | Overlap | |
| 20_35499569-35599568 | *PHACTR1* | 0.89 | 0.75 | Overlap | |
| 20_43099569-43199568 | *Unknown* | 0.878 | 0.807 | Overlap | |
| 20_43099569-43199568 | *GMDS* | 0.878 | 0.807 | Overlap | |
| 22_6990055-7090054 | *FOXI1* | 0.721 | 0.667 | LAI | |
| 22_6990055-7090054 | *DOCK2* | 0.721 | 0.667 | LAI | |
| chrUN_8003618-8103617 | *Unknown* | 0.59 | 0.769 | LAI | |
| chrUN_16803618-16903617 | *FOXN2* | 0.929 | 0.75 | Overlap | |
| chrUN_16903618-17003617 | *PPP1R21* | 0.886 | 0.772 | Overlap | |
| chrUN_17003618-17103617 | *STON1* | 0.899 | 0.875 | Overlap | |
| chrUN_17003618-17103617 | *GTF2A1L* | 0.899 | 0.875 | Overlap | |
| chrUN_17103618-17203617 | *LHCGR* | 0.816 | 0.757 | LAI | |
| 18_9475754-9575753 | *RABGEF1* | 0.805 | 0.725 | LAI | |
| 18_15775754-15875753 | *FIS1* | 0.827 | 0.738 | LAI | |
| 18_15775754-15875753 | *CLDN15* | 0.827 | 0.738 | LAI | |
| 18_15775754-15875753 | *ZNHIT1* | 0.827 | 0.738 | LAI | |
| 18_15775754-15875753 | *PLOD3* | 0.827 | 0.738 | LAI | |
| 18_15775754-15875753 | *mogat2-b* | 0.827 | 0.738 | LAI | |
| 18_15775754-15875753 | *VGF* | 0.827 | 0.738 | LAI | |
| 18_15775754-15875753 | *SERPINE1* | 0.827 | 0.738 | LAI | |
| 5_70201271-70301270 | *DARS* | 0.6 | 0.804 | LAI | |
| 5_70201271-70301270 | *MCM6* | 0.6 | 0.804 | LAI | |
| 5_70301271-70401270 | *LCT* | 0.744 | 0.875 | LAI | |
| 5_70301271-70401270 | *UBXN4* | 0.744 | 0.875 | LAI | |
| 5_70401271-70501270 | *R3HDM1* | 0.898 | 0.736 | Overlap | |
| chrUN_60550937-60650936 | *Ift81* | 0.839 | 0.598 | *f****_d_*** | |
| chrUN_60750937-60850936 | *P2RX4* | 0.886 | 0.667 | Overlap | |
| chrUN_60750937-60850936 | *CAMKK2* | 0.886 | 0.667 | Overlap | |
| chrUN_60850937-60950936 | *ANAPC5* | 0.898 | 0.75 | Overlap | |
| chrUN_60850937-60950936 | *RNF34* | 0.898 | 0.75 | Overlap | |
| chrUN_60850937-60950936 | *KDM2B* | 0.898 | 0.75 | Overlap | |
| 31_9197753-9297752 | *SOSTDC1* | 0.672 | 0.68 | LAI | |
| 31_9197753-9297752 | *ISPD* | 0.672 | 0.68 | LAI | |
| 31_9197753-9297752 | *Unknown* | 0.672 | 0.68 | LAI | |
| 8_59789730-59889729 | *GDPD5* | 0.885 | 0.625 | *f****_d_*** | |
| 8_59789730-59889729 | *KLHL35* | 0.885 | 0.625 | *f****_d_*** | |
| 8_59789730-59889729 | *RPS3* | 0.885 | 0.625 | *f****_d_*** | |
| 8_59989730-60089729 | *SLCO2B1* | 0.845 | 0.6 | *f****_d_*** | |
| 8_59989730-60089729 | *OR2AT4* | 0.845 | 0.6 | *f****_d_*** | |
| 8_60389730-60489729 | *POLD3* | 0.861 | 0.75 | Overlap | |
| 8_60489730-60589729 | *LIPT2* | 0.899 | 0.769 | Overlap | |
| 8_60489730-60589729 | *KCNE3* | 0.899 | 0.769 | Overlap | |
| 8_60589730-60689729 | *PGM2L1* | 0.924 | 0.875 | Overlap | |
| 8_60589730-60689729 | *P4HA3* | 0.924 | 0.875 | Overlap | |
| 8_60689730-60789729 | *Ppme1* | 0.929 | 0.984 | Overlap | |
| 8_60689730-60789729 | *C2CD3* | 0.929 | 0.984 | Overlap | |
| 8_60889730-60989729 | *UCP3* | 0.874 | 1 | Overlap | |
| 8_60889730-60989729 | *UCP2* | 0.874 | 1 | Overlap | |
| 8_60889730-60989729 | *DNAJB13* | 0.874 | 1 | Overlap | |
| 8_60889730-60989729 | *COA4* | 0.874 | 1 | Overlap | |
| 8_60989730-61089729 | *RAB6C* | 0.923 | 1 | Overlap | |
| 8_61089730-61189729 | *Plekhb1* | 0.938 | 1 | Overlap | |
| 8_61289730-61389729 | *Fam168a* | 0.932 | 1 | Overlap | |
| 8_61289730-61389729 | *RELT* | 0.932 | 1 | Overlap | |
| 8_61289730-61389729 | *ARHGEF17* | 0.932 | 1 | Overlap | |
| 8_61389730-61489729 | *P2RY6* | 0.918 | 1 | Overlap | |
| 8_61389730-61489729 | *P2RY2* | 0.918 | 1 | Overlap | |
| 8_61489730-61589729 | *FCHSD2* | 0.928 | 1 | Overlap | |
| 8_61689730-61789729 | *ATG16L2* | 0.933 | 1 | Overlap | |
| 8_61789730-61889729 | *STARD10* | 0.9 | 1 | Overlap | |
| 8_61789730-61889729 | *ARAP1* | 0.9 | 1 | Overlap | |
| 8_62089730-62189729 | *CLPB* | 0.883 | 1 | Overlap | |
| 8_62189730-62193908 | *PHOX2A* | 0.854 | 1 | Overlap | |
| 8_62189730-62193908 | *Inppl1* | 0.854 | 1 | Overlap | |
| 8_62189730-62193908 | *FOLR2* | 0.854 | 1 | Overlap | |
| 4_45844534-45944533 | *FOLR1* | 0.876 | 1 | Overlap | |
| 4_45844534-45944533 | *ANAPC15* | 0.876 | 1 | Overlap | |
| 4_45844534-45944533 | *LRTOMT* | 0.876 | 1 | Overlap | |
| 4_45844534-45944533 | *LAMTOR1* | 0.876 | 1 | Overlap | |
| 4_45844534-45944533 | *LRRC51* | 0.876 | 1 | Overlap | |
| 4_45944534-46044533 | *Unknown* | 0.882 | 1 | Overlap | |
| 4_45944534-46044533 | *NUMA1* | 0.882 | 1 | Overlap | |
| 4_45944534-46044533 | *IL18BP* | 0.882 | 1 | Overlap | |
| 4_45944534-46044533 | *RNF121* | 0.882 | 1 | Overlap | |
| 4_46044534-46144533 | *Trpc2* | 0.775 | 1 | LAI | |
| 4_46044534-46144533 | *ART5* | 0.775 | 1 | LAI | |
| 4_46044534-46144533 | *ART1* | 0.775 | 1 | LAI | |
| 4_46044534-46144533 | *NUP98* | 0.775 | 1 | LAI | |
| 4_46144534-46244533 | *PGAP2* | 0.844 | 1 | Overlap | |
| 4_46144534-46244533 | *Rhog* | 0.844 | 1 | Overlap | |
| 4_46144534-46244533 | *GAPDH* | 0.844 | 1 | Overlap | |
| 4_46244534-46344533 | *Stim1* | 0.825 | 1 | LAI | |
| 4_46344534-46444533 | *RRM1* | 0.836 | 0.989 | LAI | |
| 4_53944534-54044533 | *Unknown* | 0.892 | 0.5 | *f_d_* | |
|  |  |  |  |  |  |

**Table S12. Introgressed segments from alpaca into llama by using *f_d_* and LAI**

| Chromosome_start-end | Gene | *f_d_* | LAI | Methods |
| --- | --- | --- | --- | --- |
| 11_40588735-40688734 | *CDH6* | 0.464 | 0.375 | Overlap |
| 11_40688735-40788734 | *DROSHA* | 0.519 | 0.375 | Overlap |
| 11_40788735-40888734 | *C5orf22* | 0.308 | 0.375 | LAI |
| 32_7232501-7332500 | *SHC4* | 0.403 | 0.031 | *f_d_* |
| 32_7332501-7432500 | *EID1* | 0.44 | 0 | *f_d_* |
| 32_7432501-7532500 | *SECISBP2L* | 0.418 | 0 | *f_d_* |
| 32_7432501-7532500 | *Cops2* | 0.418 | 0 | *f_d_* |
| 32_7532501-7632500 | *GALK2* | 0.341 | 0 | *f_d_* |
| chrUN_36581013-36681012 | *Clcn3* | 0.32 | 0 | *f_d_* |
| chrUN_36581013-36681012 | *HPF1* | 0.32 | 0 | *f_d_* |
| chrUN_45181013-45281012 | *NKX2-6* | 0.321 | 0.25 | Overlap |
| chrUN_45281013-45381012 | *NKX3-1* | 0.293 | 0.197 | LAI |
| chrUN_45281013-45381012 | *Slc25a37* | 0.293 | 0.197 | LAI |
| 10_28217711-28317710 | *THEMIS* | 0.346 | 0.077 | *f_d_* |
| 10_29317711-29417710 | *Lama2* | 0.326 | 0 | *f_d_* |
| 6_39071995-39171994 | *GALC* | 0.181 | 0.125 | LAI |
| 25_35498514-35598513 | *LARGE2* | 0.42 | 0.246 | Overlap |
| 25_35498514-35598513 | *PEX16* | 0.42 | 0.246 | Overlap |
| 25_35498514-35598513 | *Mapk8ip1* | 0.42 | 0.246 | Overlap |
| 25_35498514-35598513 | *Cry2* | 0.42 | 0.246 | Overlap |
| 25_35598514-35698513 | *SLC35C1* | 0.231 | 0.245 | LAI |
| 24_12538448-12638447 | *SMCHD1* | 0.107 | 0.215 | LAI |
| 24_13138448-13238447 | *Dlgap1* | 0.426 | 0.08 | *f_d_* |
| 24_17138448-17238447 | *Unknown* | 0.377 | 0 | *f_d_* |
| 26_4104120-4204119 | *PPP1R3B* | 0.347 | 0.125 | Overlap |
| 26_4304120-4404119 | *TNKS* | 0.239 | 0.195 | LAI |
| 26_4504120-4604119 | *Dusp4* | 0.237 | 0.25 | LAI |
| 26_4904120-5004119 | *SARAF* | 0.525 | 0.25 | Overlap |
| 26_5004120-5104119 | *LEPROTL1* | 0.352 | 0.25 | Overlap |
| 26_5004120-5104119 | *MBOAT4* | 0.352 | 0.25 | Overlap |
| 26_5004120-5104119 | *DCTN6* | 0.352 | 0.25 | Overlap |
| 26_5104120-5204119 | *RBPMS* | 0.415 | 0.25 | Overlap |
| 26_5604120-5704119 | *PURG* | 0.327 | 0.125 | Overlap |
| 26_5604120-5704119 | *WRN* | 0.327 | 0.125 | Overlap |
| 26_5904120-6004119 | *NRG2* | 0.539 | 0.125 | Overlap |
| chrUN_112305786-112338670 | *CLEC1B* | 0.16 | 0.164 | LAI |
| chrUN_112305786-112338670 | *CLEC12B* | 0.16 | 0.164 | LAI |
| chrUN_112305786-112338670 | *CLEC12A* | 0.16 | 0.164 | LAI |
| chrUN_114338671-114438670 | *UBQLN1* | 0.408 | 0 | *f_d_* |
| chrUN_114338671-114438670 | *GKAP1* | 0.408 | 0 | *f_d_* |
| chrUN_125127894-125227893 | *PYGO1* | 0.333 | 0.184 | Overlap |
| chrUN_125127894-125227893 | *DNAAF4* | 0.333 | 0.184 | Overlap |
| chrUN_125227894-125327893 | *Unknown* | 0.46 | 0.28 | Overlap |
| chrUN_125227894-125327893 | *CCPG1* | 0.46 | 0.28 | Overlap |
| chrUN_133026188-133126187 | *GBE1* | 0.369 | 0.124 | *f_d_* |
| chrUN_133026188-133126187 | *GBE1* | 0.369 | 0.124 | *f_d_* |
| 34_10785657-10885656 | *TMTC1* | 0.324 | 0 | *f_d_* |
| 6_62653414-62753413 | *NRXN3* | 0.419 | 0.125 | Overlap |
| 11_55117369-55217368 | *RAX2* | 0.337 | 0 | *f_d_* |
| 11_55117369-55217368 | *SULT2A1* | 0.337 | 0 | *f_d_* |
| 11_55117369-55217368 | *BSPH1* | 0.337 | 0 | *f_d_* |
| 11_55117369-55217368 | *ELSPBP1* | 0.337 | 0 | *f_d_* |
| 11_55217369-55317368 | *CABP5* | 0.413 | 0 | *f_d_* |
| 11_55217369-55317368 | *LIG1* | 0.413 | 0 | *f_d_* |
| 11_55217369-55317368 | *ZSWIM9* | 0.413 | 0 | *f_d_* |
| 11_55217369-55317368 | *ZNF391* | 0.413 | 0 | *f_d_* |
| 11_55317369-55417368 | *ZNF114* | 0.374 | 0.067 | *f_d_* |
| 11_55317369-55417368 | *CCDC114* | 0.374 | 0.067 | *f_d_* |
| 11_55417369-55517368 | *EMP3* | 0.537 | 0.25 | Overlap |
| 11_55417369-55517368 | *TMEM143* | 0.537 | 0.25 | Overlap |
| 11_55417369-55517368 | *SYNGR4* | 0.537 | 0.25 | Overlap |
| 11_55417369-55517368 | *Kdelr1* | 0.537 | 0.25 | Overlap |
| 11_55417369-55517368 | *Grin2d* | 0.537 | 0.25 | Overlap |
| 11_55417369-55517368 | *GRWD1* | 0.537 | 0.25 | Overlap |
| 11_55417369-55517368 | *KCNJ14* | 0.537 | 0.25 | Overlap |
| 11_55417369-55517368 | *CYTH2* | 0.537 | 0.25 | Overlap |
| 11_55517369-55617368 | *LMTK3* | 0.425 | 0.25 | Overlap |
| 11_55517369-55617368 | *Unknown* | 0.425 | 0.25 | Overlap |
| 11_55517369-55617368 | *Sult2b1* | 0.425 | 0.25 | Overlap |
| 11_55617369-55717368 | *Fam83e* | 0.56 | 0.25 | Overlap |
| 11_55617369-55717368 | *SPACA4* | 0.56 | 0.25 | Overlap |
| 11_55617369-55717368 | *Fam83e* | 0.56 | 0.25 | Overlap |
| 11_55617369-55717368 | *RPL18* | 0.56 | 0.25 | Overlap |
| 11_55617369-55717368 | *SPHK2* | 0.56 | 0.25 | Overlap |
| 11_55617369-55717368 | *DBP* | 0.56 | 0.25 | Overlap |
| 11_55617369-55717368 | *CA11* | 0.56 | 0.25 | Overlap |
| 11_55617369-55717368 | *NTN5* | 0.56 | 0.25 | Overlap |
| 11_55617369-55717368 | *NTN5* | 0.56 | 0.25 | Overlap |
| 11_55617369-55717368 | *FUT2* | 0.56 | 0.25 | Overlap |
| 11_55717369-55817368 | *FUT2* | 0.621 | 0.25 | Overlap |
| 11_55717369-55817368 | *MAMSTR* | 0.621 | 0.25 | Overlap |
| 11_55717369-55817368 | *RASIP1* | 0.621 | 0.25 | Overlap |
| 11_55717369-55817368 | *FUT1* | 0.621 | 0.25 | Overlap |
| 11_55717369-55817368 | *FGF21* | 0.621 | 0.25 | Overlap |
| 11_55717369-55817368 | *BCAT2* | 0.621 | 0.25 | Overlap |
| 11_55717369-55817368 | *HSD17B14* | 0.621 | 0.25 | Overlap |
| 11_55817369-55917368 | *PLEKHA4* | 0.569 | 0.177 | Overlap |
| 11_55817369-55917368 | *PPP1R15A* | 0.569 | 0.177 | Overlap |
| 11_55817369-55917368 | *TULP2* | 0.569 | 0.177 | Overlap |
| 11_55817369-55917368 | *NUCB1* | 0.569 | 0.177 | Overlap |
| 11_55817369-55917368 | *DHDH* | 0.569 | 0.177 | Overlap |
| 11_55817369-55917368 | *BAX* | 0.569 | 0.177 | Overlap |
| 11_55817369-55917368 | *FTL* | 0.569 | 0.177 | Overlap |
| 11_55817369-55917368 | *GYS1* | 0.569 | 0.177 | Overlap |
| 11_55817369-55917368 | *RUVBL2* | 0.569 | 0.177 | Overlap |
| 11_57217369-57317368 | *KLK9* | 0.33 | 0 | *f_d_* |
| 11_57217369-57317368 | *KLK13* | 0.33 | 0 | *f_d_* |
| 11_57217369-57317368 | *KLK14* | 0.33 | 0 | *f_d_* |
| 11_57217369-57317368 | *CTU1* | 0.33 | 0 | *f_d_* |
| 11_57317369-57417368 | *Ceacam18* | 0.413 | 0.117 | *f_d_* |
| 11_57317369-57417368 | *CD33* | 0.413 | 0.117 | *f_d_* |
| 11_57317369-57417368 | *Siglec5* | 0.413 | 0.117 | *f_d_* |
| 11_57317369-57417368 | *SIGLECL1* | 0.413 | 0.117 | *f_d_* |
| 11_57317369-57417368 | *Iglon5* | 0.413 | 0.117 | *f_d_* |
| chrUN_177910337-178010336 | *Rin2* | 0.341 | 0.125 | Overlap |
| chrUN_177910337-178010336 | *RIN2* | 0.341 | 0.125 | Overlap |
| chrUN_178010337-178110336 | *Naa20* | 0.222 | 0.216 | LAI |
| chrUN_178010337-178110336 | *Crnkl1* | 0.222 | 0.216 | LAI |
| chrUN_178010337-178110336 | *CFAP61* | 0.222 | 0.216 | LAI |
| chrUN_179310337-179410336 | *Unknown* | 0.345 | 0 | *f_d_* |
| chrUN_179310337-179410336 | *Unknown* | 0.345 | 0 | *f_d_* |
| chrUN_179310337-179410336 | *Unknown* | 0.345 | 0 | *f_d_* |
| chrUN_179710337-179810336 | *Unknown* | 0.364 | 0.125 | Overlap |
| chrUN_179710337-179810336 | *Foxa2* | 0.364 | 0.125 | Overlap |
| chrUN_179910337-180010336 | *Unknown* | 0.384 | 0.053 | *f_d_* |
| chrUN_179910337-180010336 | *THBD* | 0.384 | 0.053 | *f_d_* |
| chrUN_180010337-180110336 | *CD93* | 0.419 | 0.125 | Overlap |
| chrUN_180010337-180110336 | *Unknown* | 0.419 | 0.125 | Overlap |
| chrUN_180110337-180210336 | *NXT1* | 0.439 | 0.125 | Overlap |
| chrUN_180110337-180210336 | *GZF1* | 0.439 | 0.125 | Overlap |
| chrUN_180110337-180210336 | *Unknown* | 0.439 | 0.125 | Overlap |
| chrUN_180110337-180210336 | *Napb* | 0.439 | 0.125 | Overlap |
| chrUN_180110337-180210336 | *CSTL1* | 0.439 | 0.125 | Overlap |
| chrUN_180110337-180210336 | *Cst11* | 0.439 | 0.125 | Overlap |
| chrUN_180110337-180210336 | *NA* | 0.439 | 0.125 | Overlap |
| chrUN_180110337-180210336 | *Cst13* | 0.439 | 0.125 | Overlap |
| chrUN_180210337-180310336 | *Cst13* | 0.458 | 0.125 | Overlap |
| chrUN_180210337-180310336 | *CST9* | 0.458 | 0.125 | Overlap |
| chrUN_180210337-180310336 | *CST3* | 0.458 | 0.125 | Overlap |
| 35_2764578-2864577 | *PRPF18* | 0.328 | 0 | *f_d_* |
| 35_2764578-2864577 | *BEND7* | 0.328 | 0 | *f_d_* |
| 11_65986931-66086930 | *ACP7* | 0.361 | 0.074 | *f_d_* |
| 11_65986931-66086930 | *PAK4* | 0.361 | 0.074 | *f_d_* |
| 11_65986931-66086930 | *FTL* | 0.361 | 0.074 | *f_d_* |
| 11_65986931-66086930 | *NCCRP1* | 0.361 | 0.074 | *f_d_* |
| 11_65986931-66086930 | *SYCN* | 0.361 | 0.074 | *f_d_* |
| 11_65986931-66086930 | *IFNL3* | 0.361 | 0.074 | *f_d_* |
| 11_65986931-66086930 | *IFNL4* | 0.361 | 0.074 | *f_d_* |
| 11_66186931-66286930 | *PAF1* | 0.357 | 0 | *f_d_* |
| 11_66186931-66286930 | *MED29* | 0.357 | 0 | *f_d_* |
| 11_66186931-66286930 | *ZFP36* | 0.357 | 0 | *f_d_* |
| 11_66186931-66286930 | *PLEKHG2* | 0.357 | 0 | *f_d_* |
| 11_66186931-66286930 | *Rps16* | 0.357 | 0 | *f_d_* |
| 11_66186931-66286930 | *Unknown* | 0.357 | 0 | *f_d_* |
| 11_66186931-66286930 | *SUPT5H* | 0.357 | 0 | *f_d_* |
| 11_66186931-66286930 | *TIMM50* | 0.357 | 0 | *f_d_* |
| 11_66186931-66286930 | *DLL3* | 0.357 | 0 | *f_d_* |
| 11_66186931-66286930 | *SELENOV* | 0.357 | 0 | *f_d_* |
| 11_66286931-66386930 | *EID2* | 0.581 | 0.194 | Overlap |
| 11_66286931-66386930 | *CLC* | 0.581 | 0.194 | Overlap |
| 11_66286931-66386930 | *LEUTX* | 0.581 | 0.194 | Overlap |
| 11_66386931-66486930 | *DYRK1B* | 0.495 | 0.336 | Overlap |
| 11_66386931-66486930 | *Fbl* | 0.495 | 0.336 | Overlap |
| 11_66386931-66486930 | *FCGBP* | 0.495 | 0.336 | Overlap |
| 11_66986931-67086930 | *RAB4B* | 0.32 | 0.125 | Overlap |
| 11_66986931-67086930 | *EGLN2* | 0.32 | 0.125 | Overlap |
| 11_66986931-67086930 | *CYP2F3* | 0.32 | 0.125 | Overlap |
| 11_66986931-67086930 | *CYP2A6* | 0.32 | 0.125 | Overlap |
| 11_66986931-67086930 | *CYP2G1* | 0.32 | 0.125 | Overlap |
| 13_57304992-57404991 | *Fubp1* | 0.321 | 0.119 | *f_d_* |
| 13_57304992-57404991 | *NEXN* | 0.321 | 0.119 | *f_d_* |
| 13_57304992-57404991 | *MIGA1* | 0.321 | 0.119 | *f_d_* |
| 20_44649610-44749609 | *ZNF76* | 0.386 | 0.125 | Overlap |
| 20_44649610-44749609 | *Unknown* | 0.386 | 0.125 | Overlap |
| 20_44649610-44749609 | *SCUBE3* | 0.386 | 0.125 | Overlap |
| 20_44749610-44849609 | *TCP11* | 0.334 | 0.125 | Overlap |
| 20_44749610-44849609 | *ANKS1A* | 0.334 | 0.125 | Overlap |
| 20_45149610-45249609 | *SPDEF* | 0.374 | 0.125 | Overlap |
| chrUN_254930484-255030483 | *SDCBP2* | 0.315 | 0.203 | LAI |
| chrUN_254930484-255030483 | *FKBP1A* | 0.315 | 0.203 | LAI |
| chrUN_254930484-255030483 | *Unknown* | 0.315 | 0.203 | LAI |
| chrUN_254930484-255030483 | *NSFL1C* | 0.315 | 0.203 | LAI |
| chrUN_255030484-255130483 | *NSFL1C* | 0.225 | 0.25 | LAI |
| chrUN_255030484-255130483 | *SIRPB2* | 0.225 | 0.25 | LAI |
| chrUN_255030484-255130483 | *SIRPB1* | 0.225 | 0.25 | LAI |
| chrUN_255030484-255130483 | *SIRPA* | 0.225 | 0.25 | LAI |
| chrUN_255130484-255230483 | *Unknown* | 0.283 | 0.199 | LAI |
| 28_15410012-15510011 | *MRPL19* | 0.425 | 0 | *f_d_* |
| 28_15410012-15510011 | *EVA1A* | 0.425 | 0 | *f_d_* |
| 20_46522732-46581870 | *Patr-A* | 0.058 | 0.15 | LAI |
| 20_46522732-46581870 | *Patr-A* | 0.058 | 0.15 | LAI |
| 20_46581871-46681870 | *Patr-A* | -0.189 | 0.212 | LAI |
| 20_46581871-46681870 | *NA* | -0.189 | 0.212 | LAI |
| 20_46581871-46681870 | *Unknown* | -0.189 | 0.212 | LAI |
| 20_46581871-46681870 | *TRIM39* | -0.189 | 0.212 | LAI |
| 20_46581871-46681870 | *PRR3* | -0.189 | 0.212 | LAI |
| 20_46881871-46981870 | *DDR1* | 0.195 | 0.199 | LAI |
| 20_46881871-46981870 | *GTF2H4* | 0.195 | 0.199 | LAI |
| 20_46881871-46981870 | *VARS2* | 0.195 | 0.199 | LAI |
| 20_46881871-46981870 | *Unknown* | 0.195 | 0.199 | LAI |
| 20_46981871-47081870 | *Dpcr1* | 0.243 | 0.25 | LAI |
| 20_46981871-47081870 | *MUC21* | 0.243 | 0.25 | LAI |
| 20_46981871-47081870 | *HCG22* | 0.243 | 0.25 | LAI |
| 20_46981871-47081870 | *CDSN* | 0.243 | 0.25 | LAI |
| 20_46981871-47081870 | *PSORS1C2* | 0.243 | 0.25 | LAI |
| 20_46981871-47081870 | *CCHCR1* | 0.243 | 0.25 | LAI |
| 20_47081871-47181870 | *TCF19* | 0.146 | 0.25 | LAI |
| 20_47081871-47181870 | *MICB* | 0.146 | 0.25 | LAI |
| 20_47081871-47181870 | *Patr-A* | 0.146 | 0.25 | LAI |
| 20_47081871-47181870 | *MCCD1* | 0.146 | 0.25 | LAI |
| 20_47081871-47181870 | *DDX39B* | 0.146 | 0.25 | LAI |
| 20_47081871-47181870 | *ATP6V1G2* | 0.146 | 0.25 | LAI |
| 20_47081871-47181870 | *NFKBIL1* | 0.146 | 0.25 | LAI |
| 20_47181871-47281870 | *LTA* | 0.227 | 0.25 | LAI |
| 20_47181871-47281870 | *TNF* | 0.227 | 0.25 | LAI |
| 20_47181871-47281870 | *LTB* | 0.227 | 0.25 | LAI |
| 20_47181871-47281870 | *LST1* | 0.227 | 0.25 | LAI |
| 20_47181871-47281870 | *AIF1* | 0.227 | 0.25 | LAI |
| 20_47181871-47281870 | *PRRC2A* | 0.227 | 0.25 | LAI |
| 20_47181871-47281870 | *BAG6* | 0.227 | 0.25 | LAI |
| 20_47181871-47281870 | *APOM* | 0.227 | 0.25 | LAI |
| 20_47181871-47281870 | *C6orf47* | 0.227 | 0.25 | LAI |
| 20_47181871-47281870 | *GPANK1* | 0.227 | 0.25 | LAI |
| 20_47181871-47281870 | *Csnk2b* | 0.227 | 0.25 | LAI |
| 20_47181871-47281870 | *LY6G5B* | 0.227 | 0.25 | LAI |
| 20_47181871-47281870 | *LY6G5C* | 0.227 | 0.25 | LAI |
| 20_47181871-47281870 | *ABHD16A* | 0.227 | 0.25 | LAI |
| 20_47281871-47381870 | *LY6G6F* | 0.208 | 0.25 | LAI |
| 20_47281871-47381870 | *Unknown* | 0.208 | 0.25 | LAI |
| 20_47281871-47381870 | *Ly6g6d* | 0.208 | 0.25 | LAI |
| 20_47281871-47381870 | *Ly6g6c* | 0.208 | 0.25 | LAI |
| 20_47281871-47381870 | *MPIG6B* | 0.208 | 0.25 | LAI |
| 20_47281871-47381870 | *DDAH2* | 0.208 | 0.25 | LAI |
| 20_47281871-47381870 | *CLIC1* | 0.208 | 0.25 | LAI |
| 20_47281871-47381870 | *MSH5* | 0.208 | 0.25 | LAI |
| 20_47281871-47381870 | *SAPCD1* | 0.208 | 0.25 | LAI |
| 20_47281871-47381870 | *VWA7* | 0.208 | 0.25 | LAI |
| 20_47281871-47381870 | *VARS* | 0.208 | 0.25 | LAI |
| 20_47281871-47381870 | *HSPA1L* | 0.208 | 0.25 | LAI |
| 20_47281871-47381870 | *HSPA1B* | 0.208 | 0.25 | LAI |
| 20_47381871-47481870 | *HSPA1B* | 0.218 | 0.22 | LAI |
| 20_47381871-47481870 | *NEU1* | 0.218 | 0.22 | LAI |
| 20_47381871-47481870 | *SLC44A4* | 0.218 | 0.22 | LAI |
| 20_47381871-47481870 | *EHMT2* | 0.218 | 0.22 | LAI |
| 20_47381871-47481870 | *ZBTB12* | 0.218 | 0.22 | LAI |
| 20_47381871-47481870 | *C2* | 0.218 | 0.22 | LAI |
| 20_47381871-47481870 | *CFB* | 0.218 | 0.22 | LAI |
| 20_47381871-47481870 | *NELFE* | 0.218 | 0.22 | LAI |
| 20_48090386-48190385 | *GPX5* | 0.09 | 0.172 | LAI |
| 20_48090386-48190385 | *GPX6* | 0.09 | 0.172 | LAI |
| 20_48090386-48190385 | *GPX6* | 0.09 | 0.172 | LAI |
| 20_48090386-48190385 | *ZSCAN12* | 0.09 | 0.172 | LAI |
| 20_48090386-48190385 | *ZSCAN31* | 0.09 | 0.172 | LAI |
| 20_48190386-48290385 | *NKAPL* | 0.354 | 0.25 | Overlap |
| 20_48190386-48290385 | *ZKSCAN4* | 0.354 | 0.25 | Overlap |
| 20_48190386-48290385 | *ZSCAN9* | 0.354 | 0.25 | Overlap |
| 20_48190386-48290385 | *ZNF850* | 0.354 | 0.25 | Overlap |
| 20_48290386-48390385 | *ZKSCAN8* | 0.197 | 0.25 | LAI |
| 20_48290386-48390385 | *OR1F12* | 0.197 | 0.25 | LAI |
| 20_48290386-48390385 | *OR2B8P* | 0.197 | 0.25 | LAI |
| 20_48390386-48490385 | *OR2B8P* | 0.141 | 0.25 | LAI |
| 20_48390386-48490385 | *OR2G6* | 0.141 | 0.25 | LAI |
| 20_48390386-48490385 | *OR2B8P* | 0.141 | 0.25 | LAI |
| 20_48390386-48490385 | *OR2W6P* | 0.141 | 0.25 | LAI |
| 20_48390386-48490385 | *OR2B6* | 0.141 | 0.25 | LAI |
| 20_48390386-48490385 | *OR2W6P* | 0.141 | 0.25 | LAI |
| 20_48390386-48490385 | *OR2B2* | 0.141 | 0.25 | LAI |
| 20_48490386-48590385 | *OR2B2* | 0.427 | 0.25 | Overlap |
| 20_48490386-48590385 | *NA* | 0.427 | 0.25 | Overlap |
| 20_48490386-48590385 | *HIST1H2AM* | 0.427 | 0.25 | Overlap |
| 20_48490386-48590385 | *NA* | 0.427 | 0.25 | Overlap |
| 20_48490386-48590385 | *TGas006m0* | 0.427 | 0.25 | Overlap |
| 20_48490386-48590385 | *NA* | 0.427 | 0.25 | Overlap |
| 20_48490386-48590385 | *Hist1h1b* | 0.427 | 0.25 | Overlap |
| 20_48490386-48590385 | *HIST1H2AM* | 0.427 | 0.25 | Overlap |
| 20_48490386-48590385 | *NA* | 0.427 | 0.25 | Overlap |
| 20_48490386-48590385 | *HIST1H2BN* | 0.427 | 0.25 | Overlap |
| 20_48490386-48590385 | *HIST1H2AM* | 0.427 | 0.25 | Overlap |
| 20_48490386-48590385 | *TGas006m0* | 0.427 | 0.25 | Overlap |
| 20_48490386-48590385 | *TGas006m0* | 0.427 | 0.25 | Overlap |
| 20_48490386-48590385 | *NA* | 0.427 | 0.25 | Overlap |
| 20_48490386-48590385 | *NA* | 0.427 | 0.25 | Overlap |
| 20_48490386-48590385 | *HIST1H2AH* | 0.427 | 0.25 | Overlap |
| 20_48490386-48590385 | *NA* | 0.427 | 0.25 | Overlap |
| 20_48590386-48690385 | *Unknown* | 0.497 | 0.25 | Overlap |
| chrUN_297810177-297825252 | *ZNF184* | 0.268 | 0.21 | LAI |
| chrUN_297810177-297825252 | *ZNF391* | 0.268 | 0.21 | LAI |
| chrUN_297810177-297825252 | *POM121L2* | 0.268 | 0.21 | LAI |
| chrUN_333270225-333370224 | *ZMIZ2* | 0.422 | 0.065 | *f_d_* |
| chrUN_336770225-336870224 | *Spns3* | 0.423 | 0.515 | Overlap |
| chrUN_337691840-337770224 | *TRIM26* | 0.103 | 0.325 | LAI |
| chrUN_337691840-337770224 | *TRIM26* | 0.103 | 0.325 | LAI |
| chrUN_337691840-337770224 | *TRIM15* | 0.103 | 0.325 | LAI |
| chrUN_337691840-337770224 | *TRIM10* | 0.103 | 0.325 | LAI |
| chrUN_337770225-337870224 | *TRIM40* | 0.307 | 0.39 | LAI |
| chrUN_337770225-337870224 | *Trim31* | 0.307 | 0.39 | LAI |
| chrUN_337770225-337870224 | *Trim31* | 0.307 | 0.39 | LAI |
| chrUN_337770225-337870224 | *RNF39* | 0.307 | 0.39 | LAI |
| chrUN_337770225-337870224 | *PPP1R11* | 0.307 | 0.39 | LAI |
| chrUN_337770225-337870224 | *ZNRD1* | 0.307 | 0.39 | LAI |
| chrUN_337770225-337870224 | *ZFP57* | 0.307 | 0.39 | LAI |
| chrUN_337870225-337970224 | *GABBR1* | 0.207 | 0.258 | LAI |
| chrUN_337870225-337970224 | *OR2H1* | 0.207 | 0.258 | LAI |
| chrUN_337870225-337970224 | *OR2I1P* | 0.207 | 0.258 | LAI |
| chrUN_337870225-337970224 | *UBD* | 0.207 | 0.258 | LAI |
| chrUN_337870225-337970224 | *OR10C1* | 0.207 | 0.258 | LAI |
| chrUN_337870225-337970224 | *OR11H2* | 0.207 | 0.258 | LAI |
| 5_4100001-4200000 | *PAX3* | 0.378 | 0.18 | Overlap |
| 12_6615433-6715432 | *Unknown* | 0.335 | 0.066 | *f_d_* |
| 12_6615433-6715432 | *SCAF11* | 0.335 | 0.066 | *f_d_* |
| 12_6715433-6815432 | *ARID2* | 0.464 | 0.125 | Overlap |
| 12_17415433-17515432 | *RTCB* | 0.36 | 0.225 | Overlap |
| 12_17415433-17515432 | *ASCL4* | 0.36 | 0.225 | Overlap |
| 12_17415433-17515432 | *PRDM4* | 0.36 | 0.225 | Overlap |
| 12_17515433-17615432 | *PWP1* | 0.448 | 0.245 | Overlap |
| 12_17515433-17615432 | *BTBD11* | 0.448 | 0.245 | Overlap |
| 12_19215433-19315432 | *NA* | 0.374 | 0.125 | Overlap |
| 12_43615433-43715432 | *ZFC3H1* | 0.379 | 0.104 | *f_d_* |
| 12_43615433-43715432 | *LGR5* | 0.379 | 0.104 | *f_d_* |
| 2_4935014-5035013 | *FRYL* | 0.29 | 0.202 | LAI |
| 2_5035014-5135013 | *ZAR1* | 0.286 | 0.25 | LAI |
| 2_5035014-5135013 | *Slc10a4* | 0.286 | 0.25 | LAI |
| 2_5035014-5135013 | *SLAIN2* | 0.286 | 0.25 | LAI |
| 2_5235014-5335013 | *Unknown* | 0.582 | 0.495 | Overlap |
| 2_5235014-5335013 | *TEC* | 0.582 | 0.495 | Overlap |
| 2_5435014-5535013 | *Unknown* | 0.488 | 0.447 | Overlap |
| 2_5435014-5535013 | *NIPAL1* | 0.488 | 0.447 | Overlap |
| 2_5435014-5535013 | *CNGA3* | 0.488 | 0.447 | Overlap |
| 2_5435014-5535013 | *NFXL1* | 0.488 | 0.447 | Overlap |
| 2_5535014-5635013 | *CORIN* | 0.524 | 0.337 | Overlap |
| 2_5735014-5835013 | *ATP10D* | 0.369 | 0.299 | Overlap |
| 2_5935014-6035013 | *GABRB1* | 0.345 | 0.288 | Overlap |
| 2_36235014-36242981 | *NR3C1* | 0.022 | 0.17 | LAI |
| 2_36235014-36242981 | *ARHGAP26* | 0.022 | 0.17 | LAI |
| 6_14972824-15072823 | *Gm527* | 0.388 | 0.225 | Overlap |
| 6_14972824-15072823 | *RPL29* | 0.388 | 0.225 | Overlap |
| 6_15072824-15172823 | *Togaram1* | 0.305 | 0.272 | LAI |
| 6_15072824-15172823 | *PRPF39* | 0.305 | 0.272 | LAI |
| 6_15172824-15272823 | *FKBP3* | 0.305 | 0.25 | LAI |
| 6_15172824-15272823 | *FANCM* | 0.305 | 0.25 | LAI |
| 6_15172824-15272823 | *MIS18BP1* | 0.305 | 0.25 | LAI |
| 6_16072824-16172823 | *RPL10L* | 0.336 | 0.125 | Overlap |
| 6_18272824-18372823 | *LRR1* | 0.354 | 0 | *f_d_* |
| 6_18272824-18372823 | *Rpl36a* | 0.354 | 0 | *f_d_* |
| 6_18272824-18372823 | *MGAT2* | 0.354 | 0 | *f_d_* |
| 6_18272824-18372823 | *DNAAF2* | 0.354 | 0 | *f_d_* |
| 6_18272824-18372823 | *POLE2* | 0.354 | 0 | *f_d_* |
| 6_19872824-19972823 | *FRMD6* | 0.319 | 0.125 | Overlap |
| 8_12800001-12900000 | *Unknown* | 0.315 | 0.232 | LAI |
| 8_12800001-12900000 | *GINM1* | 0.315 | 0.232 | LAI |
| 8_12800001-12900000 | *S100A11* | 0.315 | 0.232 | LAI |
| 8_12800001-12900000 | *KATNA1* | 0.315 | 0.232 | LAI |
| 8_12800001-12900000 | *LATS1* | 0.315 | 0.232 | LAI |
| 8_12800001-12900000 | *NUP43* | 0.315 | 0.232 | LAI |
| 8_12900001-13000000 | *NUP43* | 0.356 | 0.25 | Overlap |
| 8_12900001-13000000 | *PCMT1* | 0.356 | 0.25 | Overlap |
| 8_12900001-13000000 | *LRP11* | 0.356 | 0.25 | Overlap |
| 8_12900001-13000000 | *HNRNPA1* | 0.356 | 0.25 | Overlap |
| 8_13000001-13100000 | *FTH1* | 0.37 | 0.25 | Overlap |
| 8_13100001-13200000 | *Ppp1r14c* | 0.375 | 0.25 | Overlap |
| 8_13100001-13200000 | *Unknown* | 0.375 | 0.25 | Overlap |
| 8_13100001-13200000 | *Unknown* | 0.375 | 0.25 | Overlap |
| 8_13200001-13300000 | *IYD* | 0.38 | 0.25 | Overlap |
| 23_11217-111216 | *spen* | 0.365 | 0 | *f_d_* |
| 23_11217-111216 | *CD46* | 0.365 | 0 | *f_d_* |
| 20_25372385-25472384 | *Unknown* | 0.34 | 0.125 | Overlap |
| 20_25572385-25672384 | *BMP5* | 0.317 | 0.039 | *f_d_* |
| 4_16072817-16172816 | *ECPAS* | 0.352 | 0.125 | Overlap |
| 29_1730046-1830045 | *ST18* | 0.316 | 0.105 | *f_d_* |
| 29_16330046-16430045 | *Unknown* | 0.452 | 0.177 | Overlap |
| 29_16330046-16430045 | *Unknown* | 0.452 | 0.177 | Overlap |
| 2_43651425-43751424 | *MTTP* | 0.272 | 0.245 | LAI |
| 2_43651425-43751424 | *TRMT10A* | 0.272 | 0.245 | LAI |
| 2_43651425-43751424 | *C4orf17* | 0.272 | 0.245 | LAI |
| 2_43751425-43851424 | *NA* | 0.35 | 0.375 | Overlap |
| 2_43851425-43951424 | *ADH5* | 0.545 | 0.375 | Overlap |
| 2_43951425-44051424 | *METAP1* | 0.578 | 0.375 | Overlap |
| 2_44051425-44151424 | *Eif4e* | 0.481 | 0.348 | Overlap |
| 27_353853-453852 | *Chsy1* | 0.362 | 0.125 | Overlap |
| 19_25846-125845 | *CSNK2A1* | 0.338 | 0.125 | Overlap |
| 19_25846-125845 | *TBC1D20* | 0.338 | 0.125 | Overlap |
| 19_25846-125845 | *RBCK1* | 0.338 | 0.125 | Overlap |
| 19_925846-1025845 | *TM9SF4* | 0.402 | 0 | *f_d_* |
| 19_925846-1025845 | *TSPY26P* | 0.402 | 0 | *f_d_* |
| 19_925846-1025845 | *PLAGL2* | 0.402 | 0 | *f_d_* |
| 19_925846-1025845 | *POFUT1* | 0.402 | 0 | *f_d_* |
| 19_1625846-1725845 | *BPIFB1* | 0.345 | 0 | *f_d_* |
| 19_19825846-19925845 | *Stx16* | 0.68 | 0.375 | Overlap |
| 19_19825846-19925845 | *NPEPL1* | 0.68 | 0.375 | Overlap |
| 19_19825846-19925845 | *Piezo2* | 0.68 | 0.375 | Overlap |
| 19_19925846-20025845 | *PIEZO2* | 0.749 | 0.375 | Overlap |
| 19_19925846-20025845 | *GNAS* | 0.749 | 0.375 | Overlap |
| 19_19925846-20025845 | *Gnas* | 0.749 | 0.375 | Overlap |
| 19_20025846-20125845 | *NELFCD* | 0.671 | 0.375 | Overlap |
| 19_20025846-20125845 | *CTSZ* | 0.671 | 0.375 | Overlap |
| 19_20125846-20225845 | *TUBB1* | 0.623 | 0.375 | Overlap |
| 19_20125846-20225845 | *ATP5F1E* | 0.623 | 0.375 | Overlap |
| 19_20125846-20225845 | *PRELID3B* | 0.623 | 0.375 | Overlap |
| 19_20125846-20225845 | *Unknown* | 0.623 | 0.375 | Overlap |
| 19_20225846-20325845 | *ZNF831* | 0.642 | 0.383 | Overlap |
| 19_20325846-20425845 | *Rack1* | 0.824 | 0.605 | Overlap |
| 19_20325846-20425845 | *EDN3* | 0.824 | 0.605 | Overlap |
| 13_26941542-27041541 | *RPL22* | 0.329 | 0.125 | Overlap |
| 13_26941542-27041541 | *RNF207* | 0.329 | 0.125 | Overlap |
| 13_26941542-27041541 | *ICMT* | 0.329 | 0.125 | Overlap |
| 13_26941542-27041541 | *HES3* | 0.329 | 0.125 | Overlap |
| 13_26941542-27041541 | *Gpr153* | 0.329 | 0.125 | Overlap |
| 11_11900001-12000000 | *SORCS1* | 0.41 | 0.125 | Overlap |
| 3_45396183-45496182 | *Set* | 0.273 | 0.134 | LAI |
| 10_11158754-11258753 | *METTL15* | 0.387 | 0.164 | Overlap |
| 11_21533452-21633451 | *REEP3* | 0.342 | 0 | *f_d_* |
| 11_21933452-22033451 | *NRBF2* | 0.356 | 0.038 | *f_d_* |
| 11_33333452-33433451 | *IFIT3* | 0.17 | 0.146 | LAI |
| 11_33333452-33433451 | *IFIT1* | 0.17 | 0.146 | LAI |
| 11_33333452-33433451 | *IFIT5* | 0.17 | 0.146 | LAI |
| 11_33333452-33433451 | *SLC16A12* | 0.17 | 0.146 | LAI |
| 11_33433452-33533451 | *Unknown* | 0.226 | 0.157 | LAI |
| 11_37333452-37433451 | *CYP2C42* | 0.348 | 0.17 | Overlap |
| chrUN_7703618-7803617 | *TRMT10C* | 0.417 | 0 | *f_d_* |
| chrUN_7703618-7803617 | *ZBTB11* | 0.417 | 0 | *f_d_* |
| chrUN_15903618-16003617 | *Unknown* | 0.328 | 0.125 | Overlap |
| chrUN_15903618-16003617 | *CD200* | 0.328 | 0.125 | Overlap |
| 18_3175754-3275753 | *UQCRC2* | 0.345 | 0.089 | *f_d_* |
| 18_3175754-3275753 | *PDZD9* | 0.345 | 0.089 | *f_d_* |
| 18_3175754-3275753 | *VWA3A* | 0.345 | 0.089 | *f_d_* |
| 21_4453042-4553041 | *Tdrd5* | 0.502 | 0.125 | Overlap |
| 21_4553042-4653041 | *Nphs2* | 0.473 | 0.125 | Overlap |
| 21_14453042-14553041 | *Uck2* | 0.336 | 0.005 | *f_d_* |
| 21_14453042-14553041 | *Tmco1* | 0.336 | 0.005 | *f_d_* |
| chrUN_72050937-72150936 | *SERPINB11* | 0.318 | 0.237 | Overlap |
| chrUN_72050937-72150936 | *SERPINB4* | 0.318 | 0.237 | Overlap |
| chrUN_72050937-72150936 | *SERPINB4* | 0.318 | 0.237 | Overlap |
| chrUN_72050937-72150936 | *SERPINB4* | 0.318 | 0.237 | Overlap |
| 3_88066958-88166957 | *DYNAP* | 0.39 | 0.082 | *f_d_* |
| 3_88066958-88166957 | *DYNAP* | 0.39 | 0.082 | *f_d_* |
| 3_88066958-88166957 | *Unknown* | 0.39 | 0.082 | *f_d_* |
| 3_93466958-93566957 | *SIK3* | 0.348 | 0.25 | Overlap |
| 3_93466958-93566957 | *PAFAH1B2* | 0.348 | 0.25 | Overlap |
| 3_93466958-93566957 | *SIDT2* | 0.348 | 0.25 | Overlap |
| 3_93466958-93566957 | *TAGLN* | 0.348 | 0.25 | Overlap |
| 3_93466958-93566957 | *PCSK7* | 0.348 | 0.25 | Overlap |
| 3_93566958-93666957 | *RNF214* | 0.221 | 0.174 | LAI |
| 3_93566958-93666957 | *BACE1* | 0.221 | 0.174 | LAI |
| 3_93566958-93666957 | *CEP164* | 0.221 | 0.174 | LAI |

**Table S13. Introgressed segment and genes showing low and high LAI introgression on the inferred X-chromosome of alpaca**

| Region | Gene | Region | Gene |
| --- | --- | --- | --- |
| Low 1 | *DACH2* | High 1 | *rnf12-a* |
| Low 1 | *CHM* | High 1 | *DMD* |
| Low 1 | *ZNF711* | High 2 | *GPR101* |
| Low 1 | *Cnksr2* | High 2 | *ZIC3* |
| Low 1 | *POU3F4* | High 2 | *FGF13* |
| Low 1 | *BRWD3* | High 2 | *F9* |
| Low 1 | *TBX22* | High 3 | *BCOR* |
| Low 1 | *MAGT1* | High 3 | *ATP6AP2* |
| Low 1 | *ATRX* | High 3 | *USP9X* |
| Low 2 | *ARX* | High 3 | *DDX3X* |
| Low 2 | *POLA1* | High 3 | *NYX* |
| Low 2 | *PDK3* | High 3 | *CASK* |
| Low 3 | *OPHN1* | High 3 | *CDKL5* |
| Low 3 | *EDA2R* | High 3 | *RS1* |
| Low 4 | *CNKSR2* | High 3 | *Phka2* |
| Low 4 | *SMPX* | High 3 | *PDHA1* |
| Low 4 | *Mbtps2* | High 4 | *OFD1* |
| Low 4 | *PHEX* | High 4 | *TLR8* |
| Low 4 | *PTCHD1* | High 4 | *TLR7* |
| Low 4 | *PIH1D3* | High 4 | *Prps2* |
| Low 4 | *Prps1* | High 4 | *FRMPD4* |
| Low 4 | *Mid2* | High 4 | *Amelx* |
| Low 4 | *COL4A6* | High 4 | *MID1* |
| Low 4 | *ACSL4* | High 4 | *CLCN4* |
| Low 4 | *Ammecr1* | High 4 | *PLS3* |

**Table S14. Selection signatures detected in comparisons between vicuña and alpaca (XP-EHH)**

| Chromosome start-end | Gene | *F*_ST_ | XP-EHH |  |
| --- | --- | --- | --- | --- |
| 3 61237430-62737429 | *NAA11* | 0.287 | 4.42 | |
| 3_61237430-62737429 | *GK2* | 0.287 | 4.42 | |
| 3_61237430-62737429 | *Unknown* | 0.287 | 4.42 | |
| 3_61237430-62737429 | *ANTXR2* | 0.287 | 4.42 | |
| 3_61237430-62737429 | *Unknown* | 0.287 | 4.42 | |
| 3_61237430-62737429 | *PRDM8* | 0.287 | 4.42 | |
| 3_61237430-62737429 | *FGF5* | 0.287 | 4.42 | |
| 3_61237430-62737429 | *C4ORF22* | 0.287 | 4.42 | |
| 3_94866958-95466957 | *CCDC84* | 0.093 | 3.418 | |
| 3_94866958-95466957 | *RPS25* | 0.093 | 3.418 | |
| 3_94866958-95466957 | *TRAPPC4* | 0.093 | 3.418 | |
| 3_94866958-95466957 | *SLC37A4* | 0.093 | 3.418 | |
| 3_94866958-95466957 | *HYOU1* | 0.093 | 3.418 | |
| 3_94866958-95466957 | *VPS11* | 0.093 | 3.418 | |
| 3_94866958-95466957 | *HMBS* | 0.093 | 3.418 | |
| 3_94866958-95466957 | *H2AFX* | 0.093 | 3.418 | |
| 3_94866958-95466957 | *DPAGT1* | 0.093 | 3.418 | |
| 3_94866958-95466957 | *C2CD2L* | 0.093 | 3.418 | |
| 3_94866958-95466957 | *ABCG4* | 0.093 | 3.418 | |
| 3_94866958-95466957 | *Nlrx1* | 0.093 | 3.418 | |
| 3_94866958-95466957 | *PDZD3* | 0.093 | 3.418 | |
| 3_94866958-95466957 | *CCDC153* | 0.093 | 3.418 | |
| 3_94866958-95466957 | *Cbl* | 0.093 | 3.418 | |
| 3_94866958-95466957 | *MCAM* | 0.093 | 3.418 | |
| 3_94866958-95466957 | *RNF26* | 0.093 | 3.418 | |
| 3_94866958-95466957 | *C1QTNF5* | 0.093 | 3.418 | |
| 3_94866958-95466957 | *MFRP* | 0.093 | 3.418 | |
| 3_94866958-95466957 | *USP2* | 0.093 | 3.418 | |
| 3_94866958-95466957 | *THY1* | 0.093 | 3.418 | |
| 3_94866958-95466957 | *Unknown* | 0.093 | 3.418 | |
| 3_94866958-95466957 | *Unknown* | 0.093 | 3.418 | |
| 3_94866958-95466957 | *NECTIN1* | 0.093 | 3.418 | |
| 3_102460109-102560108 | *Rbm22* | 0.103 | 3.222 | |
| 3_102460109-102560108 | *DCTN4* | 0.103 | 3.222 | |
| 3_102460109-102560108 | *SMIM3* | 0.103 | 3.222 | |
| chrUN_47550937-48050936 | *PRF1* | 0.11 | 3.094 | |
| chrUN_47550937-48050936 | *PALD1* | 0.11 | 3.094 | |
| chrUN_47550937-48050936 | *NODAL* | 0.11 | 3.094 | |
| chrUN_47550937-48050936 | *EIF4EBP2* | 0.11 | 3.094 | |
| chrUN_47550937-48050936 | *Unknown* | 0.11 | 3.094 | |
| chrUN_47550937-48050936 | *Unknown* | 0.11 | 3.094 | |
| chrUN_47550937-48050936 | *NPFFR1* | 0.11 | 3.094 | |
| chrUN_47550937-48050936 | *Unknown* | 0.11 | 3.094 | |
| chrUN_47550937-48050936 | *PPA1* | 0.11 | 3.094 | |
| chrUN_47550937-48050936 | *TYSND1* | 0.11 | 3.094 | |
| chrUN_47550937-48050936 | *AIFM2* | 0.11 | 3.094 | |
| chrUN_47550937-48050936 | *H2AFY2* | 0.11 | 3.094 | |
| 4_14772817-14872816 | *HDHD3* | 0.163 | 3.034 | |
| 4_14772817-14872816 | *BSPRY* | 0.163 | 3.034 | |
| 4_14772817-14872816 | *WDR31* | 0.163 | 3.034 | |
| 4_14772817-14872816 | *Rnf183* | 0.163 | 3.034 | |
| 4_14772817-14872816 | *PRPF4* | 0.163 | 3.034 | |
| 4_14772817-14872816 | *CDC26* | 0.163 | 3.034 | |
| 4_14772817-14872816 | *SLC31A1* | 0.163 | 3.034 | |
| 31_1-197752 | *CREB5* | 0.096 | 2.951 | |
| 17_46187727-46885888 | *KIF9* | 0.096 | 2.885 | |
| 17_46187727-46885888 | *KLHL18* | 0.096 | 2.885 | |
| 17_46187727-46885888 | *Ngp* | 0.096 | 2.885 | |
| 17_46187727-46885888 | *Ptpn23* | 0.096 | 2.885 | |
| 17_46187727-46885888 | *SCAP* | 0.096 | 2.885 | |
| 17_46187727-46885888 | *Elp6* | 0.096 | 2.885 | |
| 17_46187727-46885888 | *CSPG5* | 0.096 | 2.885 | |
| 17_46187727-46885888 | *SMARCC1* | 0.096 | 2.885 | |
| 17_46187727-46885888 | *DHX30* | 0.096 | 2.885 | |
| 17_46187727-46885888 | *MAP4* | 0.096 | 2.885 | |
| 17_46187727-46885888 | *CDC25A* | 0.096 | 2.885 | |
| 17_46187727-46885888 | *NPG2* | 0.096 | 2.885 | |
| 17_46187727-46885888 | *PMAP37* | 0.096 | 2.885 | |
| chrUN_283454014-283854013 | *RPAIN* | 0.083 | 2.86 | |
| chrUN_283454014-283854013 | *NUP88* | 0.083 | 2.86 | |
| chrUN_283454014-283854013 | *RABEP1* | 0.083 | 2.86 | |
| chrUN_283454014-283854013 | *ZNF594* | 0.083 | 2.86 | |
| chrUN_283454014-283854013 | *ZFP3* | 0.083 | 2.86 | |
| chrUN_283454014-283854013 | *Kif1c* | 0.083 | 2.86 | |
| chrUN_283454014-283854013 | *INCA1* | 0.083 | 2.86 | |
| chrUN_283454014-283854013 | *CAMTA2* | 0.083 | 2.86 | |
| chrUN_283454014-283854013 | *ENO3* | 0.083 | 2.86 | |
| chrUN_283454014-283854013 | *PFN1* | 0.083 | 2.86 | |
| chrUN_283454014-283854013 | *RNF167* | 0.083 | 2.86 | |
| chrUN_283454014-283854013 | *SLC25A11* | 0.083 | 2.86 | |
| chrUN_283454014-283854013 | *Gp1ba* | 0.083 | 2.86 | |
| chrUN_283454014-283854013 | *CHRNE* | 0.083 | 2.86 | |
| chrUN_283454014-283854013 | *MINK1* | 0.083 | 2.86 | |
| chrUN_283454014-283854013 | *PLD2* | 0.083 | 2.86 | |
| 21_3853042-4053041 | *Lhx4* | 0.06 | 2.844 | |
| 13_27000-126999 | *HOOK1* | 0.115 | 2.749 | |
| chrUN_146456864-146556863 | *PLCB4* | 0.101 | 2.726 | |
| chrUN_259315973-259615972 | *Unknown* | 0.147 | 2.682 | |
| chrUN_259315973-259615972 | *Unknown* | 0.147 | 2.682 | |
| chrUN_259315973-259615972 | *Unknown* | 0.147 | 2.682 | |
| 20_5272385-5572384 | *Unknown* | 0.106 | 2.681 | |
| 20_5272385-5572384 | *FOXP2* | 0.106 | 2.681 | |
| chrUN_223137540-223437539 | *Unknown* | 0.046 | 2.647 | |
| chrUN_223137540-223437539 | *Unknown* | 0.046 | 2.647 | |
| chrUN_223137540-223437539 | *HS3ST3A1* | 0.046 | 2.647 | |
| chrUN_223137540-223437539 | *HS3ST3A1* | 0.046 | 2.647 | |
| 3_64437430-64537429 | *HELQ* | 0.114 | 2.63 | |
| 3_64437430-64537429 | *ABRAXAS1* | 0.114 | 2.63 | |
| chrUN_149856864-149956863 | *RASSF2* | 0.079 | 2.608 | |
| chrUN_149856864-149956863 | *PRND* | 0.079 | 2.608 | |
| chrUN_183710337-183810336 | *TRIM24* | 0.048 | 2.594 | |
| 12_20715433-21115432 | *C12orf42* | 0.137 | 2.562 | |
| 12_20715433-21115432 | *ASCL1* | 0.137 | 2.562 | |
| 12_20715433-21115432 | *PAH* | 0.137 | 2.562 | |
| 33_16916594-17016593 | *SIK2* | 0.115 | 2.56 | |
| 33_16916594-17016593 | *LAYN* | 0.115 | 2.56 | |
| 19_20225846-20325845 | *ZNF831* | 0.11 | 2.554 | |
| 6_32872824-32972823 | *ZFP36L1* | 0.084 | 2.543 | |
| 6_32872824-32972823 | *ACTN1* | 0.084 | 2.543 | |
| 5_93519328-93619327 | *SAG* | 0.088 | 2.533 | |
| 5_93519328-93619327 | *ATG16L1* | 0.088 | 2.533 | |
| 5_93519328-93619327 | *INPP5D* | 0.088 | 2.533 | |
| 8_30088785-30488784 | *GJA10* | 0.098 | 2.529 | |
| 8_30088785-30488784 | *CASP8AP2* | 0.098 | 2.529 | |
| 8_30088785-30488784 | *BACH2* | 0.098 | 2.529 | |
| 2_5535014-5935013 | *CORIN* | 0.074 | 2.507 | |
| 2_5535014-5935013 | *ATP10D* | 0.074 | 2.507 | |
| 2_60768624-60868623 | *Unknown* | 0.05 | 2.505 | |
| 24_12538448-13138447 | *SMCHD1* | 0.079 | 2.504 | |
| 24_12538448-13138447 | *EMILIN2* | 0.079 | 2.504 | |
| 24_12538448-13138447 | *LPIN2* | 0.079 | 2.504 | |
| 24_12538448-13138447 | *MYOM1* | 0.079 | 2.504 | |
| 24_12538448-13138447 | *MYL9* | 0.079 | 2.504 | |
| 24_12538448-13138447 | *Myl12b* | 0.079 | 2.504 | |
| 24_12538448-13138447 | *TGIF1* | 0.079 | 2.504 | |
| 6_33672824-33772823 | *Unknown* | 0.078 | 2.502 | |

**Table S15**. **Selection signatures detected in comparisons between vicuña and alpaca (*F*_ST_)**

| Chromosome start-end | Gene | *F*_ST_ | XP-EHH |
| --- | --- | --- | --- |
| chrUN_340870225-341070224 | *SPIN4* | 0.299 | 1.651 |
| 8_59789730-62193908 | *GDPD5* | 0.286 | 1.431 |
| 8_59789730-62193908 | *KLHL35* | 0.286 | 1.431 |
| 8_59789730-62193908 | *RPS3* | 0.286 | 1.431 |
| 8_59789730-62193908 | *ARRB1* | 0.286 | 1.431 |
| 8_59789730-62193908 | *TPBGL* | 0.286 | 1.431 |
| 8_59789730-62193908 | *SLCO2B1* | 0.286 | 1.431 |
| 8_59789730-62193908 | *OR2AT4* | 0.286 | 1.431 |
| 8_59789730-62193908 | *OR2AT4* | 0.286 | 1.431 |
| 8_59789730-62193908 | *OR2AT4* | 0.286 | 1.431 |
| 8_59789730-62193908 | *NEU3* | 0.286 | 1.431 |
| 8_59789730-62193908 | *SPCS2* | 0.286 | 1.431 |
| 8_59789730-62193908 | *XRRA1* | 0.286 | 1.431 |
| 8_59789730-62193908 | *RNF169* | 0.286 | 1.431 |
| 8_59789730-62193908 | *CHRDL2* | 0.286 | 1.431 |
| 8_59789730-62193908 | *POLD3* | 0.286 | 1.431 |
| 8_59789730-62193908 | *LIPT2* | 0.286 | 1.431 |
| 8_59789730-62193908 | *KCNE3* | 0.286 | 1.431 |
| 8_59789730-62193908 | *PGM2L1* | 0.286 | 1.431 |
| 8_59789730-62193908 | *P4HA3* | 0.286 | 1.431 |
| 8_59789730-62193908 | *Ppme1* | 0.286 | 1.431 |
| 8_59789730-62193908 | *C2CD3* | 0.286 | 1.431 |
| 8_59789730-62193908 | *UCP3* | 0.286 | 1.431 |
| 8_59789730-62193908 | *UCP2* | 0.286 | 1.431 |
| 8_59789730-62193908 | *DNAJB13* | 0.286 | 1.431 |
| 8_59789730-62193908 | *COA4* | 0.286 | 1.431 |
| 8_59789730-62193908 | *RAB6C* | 0.286 | 1.431 |
| 8_59789730-62193908 | *Plekhb1* | 0.286 | 1.431 |
| 8_59789730-62193908 | *Fam168a* | 0.286 | 1.431 |
| 8_59789730-62193908 | *RELT* | 0.286 | 1.431 |
| 8_59789730-62193908 | *ARHGEF17* | 0.286 | 1.431 |
| 8_59789730-62193908 | *P2RY6* | 0.286 | 1.431 |
| 8_59789730-62193908 | *P2RY2* | 0.286 | 1.431 |
| 8_59789730-62193908 | *FCHSD2* | 0.286 | 1.431 |
| 8_59789730-62193908 | *ATG16L2* | 0.286 | 1.431 |
| 8_59789730-62193908 | *STARD10* | 0.286 | 1.431 |
| 8_59789730-62193908 | *ARAP1* | 0.286 | 1.431 |
| 8_59789730-62193908 | *CLPB* | 0.286 | 1.431 |
| 8_59789730-62193908 | *PHOX2A* | 0.286 | 1.431 |
| 8_59789730-62193908 | *Inppl1* | 0.286 | 1.431 |
| 8_59789730-62193908 | *FOLR2* | 0.286 | 1.431 |
| 8_59789730-62193908 | *FOLR1* | 0.286 | 1.431 |
| 8_59789730-62193908 | *ANAPC15* | 0.286 | 1.431 |
| 8_59789730-62193908 | *LRTOMT* | 0.286 | 1.431 |
| 8_59789730-62193908 | *LAMTOR1* | 0.286 | 1.431 |
| 8_59789730-62193908 | *LRRC51* | 0.286 | 1.431 |
| 8_59789730-62193908 | *Unknown* | 0.286 | 1.431 |
| 8_59789730-62193908 | *NUMA1* | 0.286 | 1.431 |
| 8_59789730-62193908 | *IL18BP* | 0.286 | 1.431 |
| 8_59789730-62193908 | *RNF121* | 0.286 | 1.431 |
| 8_59789730-62193908 | *Trpc2* | 0.286 | 1.431 |
| 8_59789730-62193908 | *ART5* | 0.286 | 1.431 |
| 8_59789730-62193908 | *ART1* | 0.286 | 1.431 |
| 8_59789730-62193908 | *NUP98* | 0.286 | 1.431 |
| 8_59789730-62193908 | *PGAP2* | 0.286 | 1.431 |
| 8_59789730-62193908 | *Rhog* | 0.286 | 1.431 |
| 8_59789730-62193908 | *GAPDH* | 0.286 | 1.431 |
| 8_59789730-62193908 | *Stim1* | 0.286 | 1.431 |
| 8_59789730-62193908 | *RRM1* | 0.286 | 1.431 |
| 9_25536515-25736514 | *ZFP1* | 0.27 | -0.945 |
| 9_25536515-25736514 | *CTRB2* | 0.27 | -0.945 |
| 9_25536515-25736514 | *CTRB1* | 0.27 | -0.945 |
| 9_25536515-25736514 | *CTRB1* | 0.27 | -0.945 |
| 9_25536515-25736514 | *BCAR1* | 0.27 | -0.945 |
| 9_25536515-25736514 | *CFDP1* | 0.27 | -0.945 |
| 9_25536515-25736514 | *CFDP1* | 0.27 | -0.945 |
| chrUN_20903618-21003617 | *CHAC2* | 0.264 | 0.379 |
| chrUN_20903618-21003617 | *Unknown* | 0.264 | 0.379 |
| chrUN_20903618-21003617 | *Erlec1* | 0.264 | 0.379 |
| chrUN_20903618-21003617 | *GPR75* | 0.264 | 0.379 |
| chrUN_20903618-21003617 | *PSME4* | 0.264 | 0.379 |
| 23_28500866-28800865 | *IKBKE* | 0.259 | 0.133 |
| 23_28500866-28800865 | *Rassf5* | 0.259 | 0.133 |
| 23_28500866-28800865 | *EIF2D* | 0.259 | 0.133 |
| 23_28500866-28800865 | *DYRK3* | 0.259 | 0.133 |
| 23_28500866-28800865 | *Mapkapk2* | 0.259 | 0.133 |
| 3_61137430-63237429 | *NAA11* | 0.258 | 3.468 |
| 3_61137430-63237429 | *GK2* | 0.258 | 3.468 |
| 3_61137430-63237429 | *Unknown* | 0.258 | 3.468 |
| 3_61137430-63237429 | *ANTXR2* | 0.258 | 3.468 |
| 3_61137430-63237429 | *Unknown* | 0.258 | 3.468 |
| 3_61137430-63237429 | *PRDM8* | 0.258 | 3.468 |
| 3_61137430-63237429 | *FGF5* | 0.258 | 3.468 |
| 3_61137430-63237429 | *C4ORF22* | 0.258 | 3.468 |
| 3_61137430-63237429 | *PRKG2* | 0.258 | 3.468 |
| 3_61137430-63237429 | *RASGEF1B* | 0.258 | 3.468 |
| 21_22915279-24715278 | *Unknown* | 0.254 | 0.673 |
| 21_22915279-24715278 | *Cd24* | 0.254 | 0.673 |
| 21_22915279-24715278 | *Unknown* | 0.254 | 0.673 |
| 21_22915279-24715278 | *FBXO31* | 0.254 | 0.673 |
| 21_22915279-24715278 | *MAP1LC3B* | 0.254 | 0.673 |
| 21_22915279-24715278 | *ZCCHC14* | 0.254 | 0.673 |
| 21_22915279-24715278 | *Jph3* | 0.254 | 0.673 |
| 21_22915279-24715278 | *Jph3* | 0.254 | 0.673 |
| 21_22915279-24715278 | *Klhdc4* | 0.254 | 0.673 |
| 21_22915279-24715278 | *SLC7A5* | 0.254 | 0.673 |
| 21_22915279-24715278 | *CA5A* | 0.254 | 0.673 |
| 21_22915279-24715278 | *BANP* | 0.254 | 0.673 |
| 21_22915279-24715278 | *Unknown* | 0.254 | 0.673 |
| 21_22915279-24715278 | *Unknown* | 0.254 | 0.673 |
| 21_22915279-24715278 | *ZFPM1* | 0.254 | 0.673 |
| 21_22915279-24715278 | *Trhr* | 0.254 | 0.673 |
| 21_22915279-24715278 | *Unknown* | 0.254 | 0.673 |
| 21_22915279-24715278 | *ZC3H18* | 0.254 | 0.673 |
| 21_22915279-24715278 | *IL17C* | 0.254 | 0.673 |
| 21_22915279-24715278 | *CYBA* | 0.254 | 0.673 |
| 21_22915279-24715278 | *MVD* | 0.254 | 0.673 |
| 21_22915279-24715278 | *SNAI3* | 0.254 | 0.673 |
| 21_22915279-24715278 | *RNF166* | 0.254 | 0.673 |
| 21_22915279-24715278 | *CTU2* | 0.254 | 0.673 |
| 21_22915279-24715278 | *PIEZO1* | 0.254 | 0.673 |
| 21_22915279-24715278 | *CDT1* | 0.254 | 0.673 |
| 21_22915279-24715278 | *APRT* | 0.254 | 0.673 |
| 21_22915279-24715278 | *GALNS* | 0.254 | 0.673 |
| 21_22915279-24715278 | *Trappc2l* | 0.254 | 0.673 |
| 21_22915279-24715278 | *CBFA2T3* | 0.254 | 0.673 |
| 21_22915279-24715278 | *Unknown* | 0.254 | 0.673 |
| 21_22915279-24715278 | *Unknown* | 0.254 | 0.673 |
| 21_22915279-24715278 | *ACSF3* | 0.254 | 0.673 |
| 21_22915279-24715278 | *Cdh15* | 0.254 | 0.673 |
| 21_22915279-24715278 | *SLC22A31* | 0.254 | 0.673 |
| 21_22915279-24715278 | *ANKRD11* | 0.254 | 0.673 |
| 21_22915279-24715278 | *Unknown* | 0.254 | 0.673 |
| 21_22915279-24715278 | *SPG7* | 0.254 | 0.673 |
| 21_22915279-24715278 | *Cpne7* | 0.254 | 0.673 |
| 21_22915279-24715278 | *DPEP1* | 0.254 | 0.673 |
| 21_22915279-24715278 | *CHMP1A* | 0.254 | 0.673 |
| 21_22915279-24715278 | *SPATA33* | 0.254 | 0.673 |
| 21_22915279-24715278 | *CDK10* | 0.254 | 0.673 |
| 21_22915279-24715278 | *SPATA2L* | 0.254 | 0.673 |
| 21_22915279-24715278 | *VPS9D1* | 0.254 | 0.673 |
| 21_22915279-24715278 | *ZNF276* | 0.254 | 0.673 |
| 21_22915279-24715278 | *FANCA* | 0.254 | 0.673 |
| 21_22915279-24715278 | *Spire2* | 0.254 | 0.673 |
| 21_22915279-24715278 | *SPIRE2* | 0.254 | 0.673 |
| 21_22915279-24715278 | *TCF25* | 0.254 | 0.673 |
| 21_22915279-24715278 | *MC1R* | 0.254 | 0.673 |
| 21_22915279-24715278 | *TUBB3* | 0.254 | 0.673 |
| 21_22915279-24715278 | *Afg3l1* | 0.254 | 0.673 |
| 21_22915279-24715278 | *Afg3l1* | 0.254 | 0.673 |
| 21_22915279-24715278 | *DBNDD1* | 0.254 | 0.673 |
|  |  |  |  |

**Table S16. Selection signatures detected in comparisons between vicuña and alpaca (overlap between methods)**

| Chromosome start-end | Gene | *F*_ST_ | XP-EHH |
| --- | --- | --- | --- |
| 3_61237430-62737429 | *NAA11* | 0.287 | 4.42 |
| 3_61237430-62737429 | *GK2* | 0.287 | 4.42 |
| 3_61237430-62737429 | *Unknown* | 0.287 | 4.42 |
| 3_61237430-62737429 | *ANTXR2* | 0.287 | 4.42 |
| 3_61237430-62737429 | *Unknown* | 0.287 | 4.42 |
| 3_61237430-62737429 | *PRDM8* | 0.287 | 4.42 |
| 3_61237430-62737429 | *FGF5* | 0.287 | 4.42 |
| 3_61237430-62737429 | *C4ORF22* | 0.287 | 4.42 |
| chrUN_95380520-95480519 | *EXOC4* | 0.22 | 2.173 |
| chrUN_17103618-17203617 | *LHCGR* | 0.206 | 1.906 |
| 4_45748813-46444533 | *ATG16L2* | 0.298 | 2.424 |
| 4_45748813-46444533 | *STARD10* | 0.298 | 2.424 |
| 4_45748813-46444533 | *ARAP1* | 0.298 | 2.424 |
| 4_45748813-46444533 | *CLPB* | 0.298 | 2.424 |
| 4_45748813-46444533 | *PHOX2A* | 0.298 | 2.424 |
| 4_45748813-46444533 | *Inppl1* | 0.298 | 2.424 |
| 4_45748813-46444533 | *FOLR2* | 0.298 | 2.424 |
| 4_45748813-46444533 | *FOLR1* | 0.298 | 2.424 |
| 4_45748813-46444533 | *ANAPC15* | 0.298 | 2.424 |
| 4_45748813-46444533 | *LRTOMT* | 0.298 | 2.424 |
| 4_45748813-46444533 | *LAMTOR1* | 0.298 | 2.424 |
| 4_45748813-46444533 | *LRRC51* | 0.298 | 2.424 |
| 4_45748813-46444533 | *Unknown* | 0.298 | 2.424 |
| 4_45748813-46444533 | *NUMA1* | 0.298 | 2.424 |
| 4_45748813-46444533 | *IL18BP* | 0.298 | 2.424 |
| 4_45748813-46444533 | *RNF121* | 0.298 | 2.424 |
| 4_45748813-46444533 | *Trpc2* | 0.298 | 2.424 |
| 4_45748813-46444533 | *ART5* | 0.298 | 2.424 |
| 4_45748813-46444533 | *ART1* | 0.298 | 2.424 |
| 4_45748813-46444533 | *NUP98* | 0.298 | 2.424 |
| 4_45748813-46444533 | *PGAP2* | 0.298 | 2.424 |
| 4_45748813-46444533 | *Rhog* | 0.298 | 2.424 |
| 4_45748813-46444533 | *GAPDH* | 0.298 | 2.424 |
| 4_45748813-46444533 | *Stim1* | 0.298 | 2.424 |
| 4_45748813-46444533 | *RRM1* | 0.298 | 2.424 |
|  |  |  |  |

**Table S17**. **Selection signatures detected in comparisons between guanaco and llama (XP-EHH)**

| Chromosome start-end | Gene | *F*_ST_ | XP-EHH |
| --- | --- | --- | --- |
| 16_27064595-27364594 | *Neurod2* | 0.129 | 3.414 |
| 16_27064595-27364594 | *PPP1R1B* | 0.129 | 3.414 |
| 16_27064595-27364594 | *Stard3* | 0.129 | 3.414 |
| 16_27064595-27364594 | *TCAP* | 0.129 | 3.414 |
| 16_27064595-27364594 | *PNMT* | 0.129 | 3.414 |
| 16_27064595-27364594 | *PGAP3* | 0.129 | 3.414 |
| 16_27064595-27364594 | *ERBB2* | 0.129 | 3.414 |
| 16_27064595-27364594 | *MIEN1* | 0.129 | 3.414 |
| 16_27064595-27364594 | *GRB7* | 0.129 | 3.414 |
| 16_27064595-27364594 | *Unknown* | 0.129 | 3.414 |
| 16_27064595-27364594 | *IKZF3* | 0.129 | 3.414 |
| 16_27064595-27364594 | *ZPBP2* | 0.129 | 3.414 |
| 16_27064595-27364594 | *GSDMB* | 0.129 | 3.414 |
| 16_27064595-27364594 | *ORMDL3* | 0.129 | 3.414 |
| 16_27064595-27364594 | *LRRC3C* | 0.129 | 3.414 |
| 2_107277008-108177007 | *ARSJ* | 0.107 | 2.858 |
| 2_107277008-108177007 | *CAMK2D* | 0.107 | 2.858 |
| 13_5627000-6026999 | *PODN* | 0.068 | 2.857 |
| 13_5627000-6026999 | *SCP2* | 0.068 | 2.857 |
| 13_5627000-6026999 | *ECHDC2* | 0.068 | 2.857 |
| 13_5627000-6026999 | *ZYG11A* | 0.068 | 2.857 |
| 13_5627000-6026999 | *SERF2* | 0.068 | 2.857 |
| 13_5627000-6026999 | *ZYG11B* | 0.068 | 2.857 |
| 13_5627000-6026999 | *Coa7* | 0.068 | 2.857 |
| 13_5627000-6026999 | *SHISAL2A* | 0.068 | 2.857 |
| 13_4727000-5126999 | *MRPL37* | 0.076 | 2.837 |
| 13_4727000-5126999 | *CYB5RL* | 0.076 | 2.837 |
| 13_4727000-5126999 | *CDCP2* | 0.076 | 2.837 |
| 13_4727000-5126999 | *TCEANC2* | 0.076 | 2.837 |
| 13_4727000-5126999 | *TMEM59* | 0.076 | 2.837 |
| 13_4727000-5126999 | *LDLRAD1* | 0.076 | 2.837 |
| 13_4727000-5126999 | *LRRC42* | 0.076 | 2.837 |
| 13_4727000-5126999 | *RPS2* | 0.076 | 2.837 |
| 13_4727000-5126999 | *HSPB11* | 0.076 | 2.837 |
| 13_4727000-5126999 | *DIO1* | 0.076 | 2.837 |
| 13_4727000-5126999 | *YIPF1* | 0.076 | 2.837 |
| 13_4727000-5126999 | *NDC1* | 0.076 | 2.837 |
| 13_4727000-5126999 | *Glis1* | 0.076 | 2.837 |
| chrUN_322050946-322070224 | *TRIM11* | 0.123 | 2.837 |
| chrUN_322050946-322070224 | *TRIM17* | 0.123 | 2.837 |
| chrUN_322050946-322070224 | *Unknown* | 0.123 | 2.837 |
| chrUN_322050946-322070224 | *Hist3h2bb* | 0.123 | 2.837 |
| chrUN_322050946-322070224 | *RNF187* | 0.123 | 2.837 |
| chrUN_322050946-322070224 | *Olfr56* | 0.123 | 2.837 |
| chrUN_322050946-322070224 | *OR2L13* | 0.123 | 2.837 |
| chrUN_322050946-322070224 | *Olfr56* | 0.123 | 2.837 |
| chrUN_322050946-322070224 | *OR2T27* | 0.123 | 2.837 |
| 14_13744126-13844125 | *USP12* | 0.164 | 2.749 |
| 19_10525846-10625845 | *MATN4* | 0.102 | 2.671 |
| 19_10525846-10625845 | *RBPJL* | 0.102 | 2.671 |
| 19_10525846-10625845 | *SDC4* | 0.102 | 2.671 |
| 19_10525846-10625845 | *SYS1* | 0.102 | 2.671 |
| 19_10525846-10625845 | *TP53TG5* | 0.102 | 2.671 |
| 19_10525846-10625845 | *DBNDD2* | 0.102 | 2.671 |
| 19_10525846-10625845 | *PIGT* | 0.102 | 2.671 |
| 3_61137430-63237429 | *NAA11* | 0.106 | 2.629 |
| 3_61137430-63237429 | *GK2* | 0.106 | 2.629 |
| 3_61137430-63237429 | *Unknown* | 0.106 | 2.629 |
| 3_61137430-63237429 | *ANTXR2* | 0.106 | 2.629 |
| 3_61137430-63237429 | *Unknown* | 0.106 | 2.629 |
| 3_61137430-63237429 | *PRDM8* | 0.106 | 2.629 |
| 3_61137430-63237429 | *FGF5* | 0.106 | 2.629 |
| 3_61137430-63237429 | *C4ORF22* | 0.106 | 2.629 |
| 3_61137430-63237429 | *PRKG2* | 0.106 | 2.629 |
| 3_61137430-63237429 | *RASGEF1B* | 0.106 | 2.629 |
| 27_17653853-17953852 | *Sin3a* | 0.097 | 2.624 |
| 27_17653853-17953852 | *MAN2C1* | 0.097 | 2.624 |
| 27_17653853-17953852 | *NEIL1* | 0.097 | 2.624 |
| 27_17653853-17953852 | *COMMD4* | 0.097 | 2.624 |
| 27_17653853-17953852 | *Trcg1* | 0.097 | 2.624 |
| 27_17653853-17953852 | *Unknown* | 0.097 | 2.624 |
| 27_17653853-17953852 | *PPCDC* | 0.097 | 2.624 |
| 27_17653853-17953852 | *SCAMP5* | 0.097 | 2.624 |
| 27_17653853-17953852 | *Rpp25* | 0.097 | 2.624 |
| 25_52386813-52486812 | *SCGB1A1* | 0.057 | 2.601 |
| 13_15227000-15426999 | *RLF* | 0.113 | 2.574 |
| 13_15227000-15426999 | *PPT1* | 0.113 | 2.574 |
| 13_15227000-15426999 | *CAP1* | 0.113 | 2.574 |
| 18_14075754-14275753 | *Mlxipl* | 0.09 | 2.564 |
| 18_14075754-14275753 | *TBL2* | 0.09 | 2.564 |
| 18_14075754-14275753 | *BCL7B* | 0.09 | 2.564 |
| 18_14075754-14275753 | *BAZ1B* | 0.09 | 2.564 |
| 18_14075754-14275753 | *Fzd9* | 0.09 | 2.564 |
| 18_14075754-14275753 | *FKBP6* | 0.09 | 2.564 |
| 18_14075754-14275753 | *TRIM50* | 0.09 | 2.564 |
| 18_14075754-14275753 | *NSUN5* | 0.09 | 2.564 |
| 4_8772817-8972816 | *PHF19* | 0.084 | 2.557 |
| 4_8772817-8972816 | *cuta* | 0.084 | 2.557 |
| 4_8772817-8972816 | *PSMD5* | 0.084 | 2.557 |
| 4_8772817-8972816 | *UnKnown* | 0.084 | 2.557 |
| 4_8772817-8972816 | *FBXW2* | 0.084 | 2.557 |
| 4_8772817-8972816 | *Unknown* | 0.084 | 2.557 |
| 4_8772817-8972816 | *MEGF9* | 0.084 | 2.557 |
| 3_23891933-25591932 | *Unknown* | 0.067 | 2.51 |
| 3_23891933-25591932 | *STARD4* | 0.067 | 2.51 |
| 3_23891933-25591932 | *CAMK4* | 0.067 | 2.51 |
| 3_23891933-25591932 | *CAMK4* | 0.067 | 2.51 |
| 3_23891933-25591932 | *WDR36* | 0.067 | 2.51 |
| 3_23891933-25591932 | *SLC25A46* | 0.067 | 2.51 |
| 3_23891933-25591932 | *TMEM232* | 0.067 | 2.51 |
| 3_23891933-25591932 | *TMEM232* | 0.067 | 2.51 |
| 3_23891933-25591932 | *MAN2A1* | 0.067 | 2.51 |

**Table S18**. **Selection signatures detected in comparisons between guanaco and llama (*F*_ST_)**

| Chromosome _start-end | Gene | *F*_ST_ | XP-EHH |
| --- | --- | --- | --- |
| chrUN_259315973-259615972 | *Unknown* | 0.358 | 0.326 |
| chrUN_259315973-259615972 | *Unknown* | 0.358 | 0.326 |
| chrUN_259315973-259615972 | *Unknown* | 0.358 | 0.326 |
| 27_18253853-18353852 | *UBL7* | 0.249 | 1.473 |
| 27_18253853-18353852 | *UBL7* | 0.249 | 1.473 |
| 27_18253853-18353852 | *SEMA7A* | 0.249 | 1.473 |
| 22_16944450-17844449 | *C2cd4cC2C* | 0.234 | 0.8 |
| 22_16944450-17844449 | *Shc2* | 0.234 | 0.8 |
| 22_16944450-17844449 | *Unknown* | 0.234 | 0.8 |
| 22_16944450-17844449 | *Madcam1* | 0.234 | 0.8 |
| 22_16944450-17844449 | *TPGS1* | 0.234 | 0.8 |
| 22_16944450-17844449 | *CDC34* | 0.234 | 0.8 |
| 22_16944450-17844449 | *GZMM* | 0.234 | 0.8 |
| 22_16944450-17844449 | *BSG* | 0.234 | 0.8 |
| 22_16944450-17844449 | *Hcn2* | 0.234 | 0.8 |
| 22_16944450-17844449 | *POLRMT* | 0.234 | 0.8 |
| 22_16944450-17844449 | *Fgf22* | 0.234 | 0.8 |
| 22_16944450-17844449 | *RNF126* | 0.234 | 0.8 |
| 22_16944450-17844449 | *FSTL3* | 0.234 | 0.8 |
| 22_16944450-17844449 | *PRSS57* | 0.234 | 0.8 |
| 22_16944450-17844449 | *PALM* | 0.234 | 0.8 |
| 22_16944450-17844449 | *MISP* | 0.234 | 0.8 |
| 22_16944450-17844449 | *PTBP1* | 0.234 | 0.8 |
| 22_16944450-17844449 | *Plppr3* | 0.234 | 0.8 |
| 22_16944450-17844449 | *AZU1* | 0.234 | 0.8 |
| 22_16944450-17844449 | *Unknown* | 0.234 | 0.8 |
| 22_16944450-17844449 | *Elane* | 0.234 | 0.8 |
| 22_16944450-17844449 | *CFD* | 0.234 | 0.8 |
| 22_16944450-17844449 | *MED16* | 0.234 | 0.8 |
| 22_16944450-17844449 | *R3HDM4* | 0.234 | 0.8 |
| 22_16944450-17844449 | *KISS1R* | 0.234 | 0.8 |
| 22_16944450-17844449 | *ARID3A* | 0.234 | 0.8 |
| 22_16944450-17844449 | *WDR18* | 0.234 | 0.8 |
| 22_16944450-17844449 | *GRIN3B* | 0.234 | 0.8 |
| 22_16944450-17844449 | *TMEM259* | 0.234 | 0.8 |
| 22_16944450-17844449 | *CNN2* | 0.234 | 0.8 |
| 22_16944450-17844449 | *ABCA7* | 0.234 | 0.8 |
| 22_16944450-17844449 | *ARHGAP45* | 0.234 | 0.8 |
| 22_16944450-17844449 | *POLR2E* | 0.234 | 0.8 |
| 22_16944450-17844449 | *GPX4* | 0.234 | 0.8 |
| 22_16944450-17844449 | *SBNO2* | 0.234 | 0.8 |
| 22_16944450-17844449 | *SBNO2* | 0.234 | 0.8 |
| 22_16944450-17844449 | *STK11* | 0.234 | 0.8 |
| 22_16944450-17844449 | *Cbarp* | 0.234 | 0.8 |
| 22_16944450-17844449 | *ATP5F1D* | 0.234 | 0.8 |
| 22_16944450-17844449 | *Midn* | 0.234 | 0.8 |
| 22_16944450-17844449 | *CIRBP* | 0.234 | 0.8 |
| 22_16944450-17844449 | *C19orf24* | 0.234 | 0.8 |
| 22_16944450-17844449 | *Efna2* | 0.234 | 0.8 |
| 22_16944450-17844449 | *MUM1* | 0.234 | 0.8 |
| 22_16944450-17844449 | *NDUFS7* | 0.234 | 0.8 |
| 22_16944450-17844449 | *GAMT* | 0.234 | 0.8 |
| 22_16944450-17844449 | *Dazap1* | 0.234 | 0.8 |
| 22_16944450-17844449 | *Rps15* | 0.234 | 0.8 |
| 22_16944450-17844449 | *APC2* | 0.234 | 0.8 |
| 22_16944450-17844449 | *Unknown* | 0.234 | 0.8 |
| 22_16944450-17844449 | *PCSK4* | 0.234 | 0.8 |
| 22_16944450-17844449 | *REEP6* | 0.234 | 0.8 |
| 22_16944450-17844449 | *ADAMTSL5* | 0.234 | 0.8 |
| 22_16944450-17844449 | *Plk5* | 0.234 | 0.8 |
| 22_16944450-17844449 | *MEX3D* | 0.234 | 0.8 |
| 22_16944450-17844449 | *Uqcr11* | 0.234 | 0.8 |
| 22_16944450-17844449 | *TCF3* | 0.234 | 0.8 |
| 19_19725846-20525845 | *Stx16* | 0.231 | -0.694 |
| 19_19725846-20525845 | *NPEPL1* | 0.231 | -0.694 |
| 19_19725846-20525845 | *Piezo2* | 0.231 | -0.694 |
| 19_19725846-20525845 | *PIEZO2* | 0.231 | -0.694 |
| 19_19725846-20525845 | *GNAS* | 0.231 | -0.694 |
| 19_19725846-20525845 | *NELFCD* | 0.231 | -0.694 |
| 19_19725846-20525845 | *CTSZ* | 0.231 | -0.694 |
| 19_19725846-20525845 | *TUBB1* | 0.231 | -0.694 |
| 19_19725846-20525845 | *ATP5F1E* | 0.231 | -0.694 |
| 19_19725846-20525845 | *PRELID3B* | 0.231 | -0.694 |
| 19_19725846-20525845 | *Unknown* | 0.231 | -0.694 |
| 19_19725846-20525845 | *ZNF831* | 0.231 | -0.694 |
| 19_19725846-20525845 | *Rack1* | 0.231 | -0.694 |
| 19_19725846-20525845 | *EDN3* | 0.231 | -0.694 |
| 5_93319328-93519327 | *USP40* | 0.227 | 0.45 |
| 5_93319328-93519327 | *DGKD* | 0.227 | 0.45 |
| 33_8239455-8339454 | *TMPRSS6* | 0.223 | 0.001 |
| 33_8239455-8339454 | *IL2RB* | 0.223 | 0.001 |
| 33_8239455-8339454 | *C1qtnf6* | 0.223 | 0.001 |
| 3_80327083-80527082 | *DUSP13* | 0.223 | 0.227 |
| 3_80327083-80527082 | *DUPD1* | 0.223 | 0.227 |
| 5_40300001-40400000 | *GPR155* | 0.217 | 1.222 |
| 5_40300001-40400000 | *SCRN3* | 0.217 | 1.222 |
| 5_40300001-40400000 | *CIR1* | 0.217 | 1.222 |
| chrUN_340870225-341070224 | *SPIN4* | 0.21 | 0.664 |
| 35_2764578-2864577 | *PRPF18* | 0.209 | 0.788 |
| 35_2764578-2864577 | *BEND7* | 0.209 | 0.788 |
| 6_46228104-46528103 | *AMN* | 0.204 | 0.546 |
| 6_46228104-46528103 | *Traf3* | 0.204 | 0.546 |
| 6_46228104-46528103 | *RCOR1* | 0.204 | 0.546 |
| 32_18922638-18926042 | *brk1* | 0.203 | 0.395 |
| 32_18922638-18926042 | *PPP1R3F* | 0.203 | 0.395 |
| 32_18922638-18926042 | *FOXP3* | 0.203 | 0.395 |
| 32_18922638-18926042 | *CCDC22* | 0.203 | 0.395 |
| 32_18922638-18926042 | *CACNA1F* | 0.203 | 0.395 |
| 32_18922638-18926042 | *SYP* | 0.203 | 0.395 |
| 32_18922638-18926042 | *PRICKLE3* | 0.203 | 0.395 |
| 32_18922638-18926042 | *PLP2* | 0.203 | 0.395 |
| 32_18922638-18926042 | *MAGIX* | 0.203 | 0.395 |
| 32_18922638-18926042 | *GPKOW* | 0.203 | 0.395 |
| 32_18922638-18926042 | *WDR45* | 0.203 | 0.395 |
| 32_18922638-18926042 | *PRAF2* | 0.203 | 0.395 |
| 32_18922638-18926042 | *CCDC120* | 0.203 | 0.395 |
| 32_18922638-18926042 | *TFE3* | 0.203 | 0.395 |
| 32_18922638-18926042 | *GRIPAP1* | 0.203 | 0.395 |
| 32_18922638-18926042 | *KCND1* | 0.203 | 0.395 |
| 32_18922638-18926042 | *Otud5* | 0.203 | 0.395 |
| 32_18922638-18926042 | *PIM2* | 0.203 | 0.395 |
| 32_18922638-18926042 | *SLC35A2* | 0.203 | 0.395 |
| 32_18922638-18926042 | *PQBP1* | 0.203 | 0.395 |
| 32_18922638-18926042 | *TIMM17B* | 0.203 | 0.395 |
| 32_18922638-18926042 | *Syt5* | 0.203 | 0.395 |
| 32_18922638-18926042 | *Unknown* | 0.203 | 0.395 |
| 32_18922638-18926042 | *PCSK1N* | 0.203 | 0.395 |
| 32_18922638-18926042 | *ERAS* | 0.203 | 0.395 |
| 32_18922638-18926042 | *HDAC6* | 0.203 | 0.395 |
| 32_18922638-18926042 | *GATA1* | 0.203 | 0.395 |
| 32_18922638-18926042 | *GLOD5* | 0.203 | 0.395 |
| 32_18922638-18926042 | *SUV39H1* | 0.203 | 0.395 |
| 32_18922638-18926042 | *WAS* | 0.203 | 0.395 |
| 32_18922638-18926042 | *Unknown* | 0.203 | 0.395 |
| 15_12459970-12559969 | *SLC30A6* | 0.2 | 0.605 |
| 15_12459970-12559969 | *NLRC4* | 0.2 | 0.605 |

**Table S19. Selection signatures detected in comparisons between guanaco and llama (overlap between methods)**

| Chromosome start-end | Gene | *F*_ST_ | XP-EHH |
| --- | --- | --- | --- |
| 3_55937430-56037429 | *GC* | 0.147 | 1.89 |
| 33_11239455-11339454 | *RANGAP1* | 0.182 | 1.754 |
| 33_11239455-11339454 | *ZC3H7B* | 0.182 | 1.754 |
| 22_18844450-18944449 | *GNA11* | 0.151 | 2.196 |
| 22_18844450-18944449 | *GNA15* | 0.151 | 2.196 |
| 22_18844450-18944449 | *S1PR4* | 0.151 | 2.196 |
| 22_18844450-18944449 | *NCLN* | 0.151 | 2.196 |
| 22_18844450-18944449 | *MAP2K2* | 0.15 | 3.104 |
| 22_18844450-18944449 | *CREB3L3* | 0.15 | 3.104 |
| 22_18844450-18944449 | *SIRT6* | 0.15 | 3.104 |
| 22_18844450-18944449 | *ANKRD24* | 0.15 | 3.104 |
| 22_18844450-18944449 | *YJU2* | 0.15 | 3.104 |
| 33_16816594-16916593 | *PPP2R1B* | 0.162 | 1.82 |
| chrUN_248757006-248857005 | *GRTP1* | 0.16 | 1.828 |
| chrUN_248757006-248857005 | *ADPRHL1* | 0.16 | 1.828 |
| chrUN_248757006-248857005 | *Unknown* | 0.16 | 1.828 |
| chrUN_248757006-248857005 | *Tmco3* | 0.16 | 1.828 |
| chrUN_258530484-259030483 | *Uncx* | 0.15 | 2.802 |
| chrUN_258530484-259030483 | *ZFAND2A* | 0.15 | 2.802 |
| chrUN_258530484-259030483 | *GPER1* | 0.15 | 2.802 |
| chrUN_258530484-259030483 | *Unknown* | 0.15 | 2.802 |
| chrUN_258530484-259030483 | *Unknown* | 0.15 | 2.802 |
| chrUN_258530484-259030483 | *Gpr146* | 0.15 | 2.802 |
| chrUN_258530484-259030483 | *C7orf50* | 0.15 | 2.802 |
| chrUN_258530484-259030483 | *CYP2W1* | 0.15 | 2.802 |
| chrUN_258530484-259030483 | *COX19* | 0.15 | 2.802 |
| chrUN_258530484-259030483 | *ADAP1* | 0.15 | 2.802 |
| chrUN_258530484-259030483 | *GET4* | 0.15 | 2.802 |
| chrUN_258530484-259030483 | *Sun1* | 0.15 | 2.802 |
| chrUN_258530484-259030483 | *Unknown* | 0.15 | 2.802 |
| chrUN_258530484-259030483 | *Dnaaf5* | 0.15 | 2.802 |
| chrUN_258530484-259030483 | *PRKAR1B* | 0.15 | 2.802 |
| chrUN_258530484-259030483 | *PDGFA* | 0.15 | 2.802 |
| chrUN_298125253-298325252 | *Glud1* | 0.184 | 1.807 |
| chrUN_298125253-298325252 | *GLUD2* | 0.184 | 1.807 |
| chrUN_298125253-298325252 | *Unknown* | 0.184 | 1.807 |
| chrUN_316870225-317370224 | *GLS2* | 0.15 | 2.298 |
| chrUN_316870225-317370224 | *SPRYD4* | 0.15 | 2.298 |
| chrUN_316870225-317370224 | *MIP* | 0.15 | 2.298 |
| chrUN_316870225-317370224 | *TIMELESS* | 0.15 | 2.298 |
| chrUN_316870225-317370224 | *APOF* | 0.15 | 2.298 |
| chrUN_316870225-317370224 | *STAT2* | 0.15 | 2.298 |
| chrUN_316870225-317370224 | *IL23A* | 0.15 | 2.298 |
| chrUN_316870225-317370224 | *PAN2* | 0.15 | 2.298 |
| chrUN_316870225-317370224 | *CS* | 0.15 | 2.298 |
| chrUN_316870225-317370224 | *COQ10A* | 0.15 | 2.298 |
| chrUN_316870225-317370224 | *Ankrd52* | 0.15 | 2.298 |
| chrUN_316870225-317370224 | *Slc39a5* | 0.15 | 2.298 |
| chrUN_316870225-317370224 | *RNF41* | 0.15 | 2.298 |
| chrUN_316870225-317370224 | *SMARCC2* | 0.15 | 2.298 |
| chrUN_316870225-317370224 | *MYL6B* | 0.15 | 2.298 |
| chrUN_316870225-317370224 | *ESYT1* | 0.15 | 2.298 |
| chrUN_316870225-317370224 | *Zc3h10* | 0.15 | 2.298 |
| chrUN_316870225-317370224 | *ERBB3* | 0.15 | 2.298 |
| chrUN_316870225-317370224 | *IKZF4* | 0.15 | 2.298 |
| chrUN_316870225-317370224 | *SUOX* | 0.15 | 2.298 |
| chrUN_316870225-317370224 | *RAB5B* | 0.15 | 2.298 |
| chrUN_316870225-317370224 | *Cdk2* | 0.15 | 2.298 |
| chrUN_316870225-317370224 | *PMEL* | 0.15 | 2.298 |
| chrUN_316870225-317370224 | *DGKA* | 0.15 | 2.298 |
| chrUN_316870225-317370224 | *PYM1* | 0.15 | 2.298 |
| chrUN_316870225-317370224 | *MMP19* | 0.15 | 2.298 |
| chrUN_321870225-321970224 | *TRIM11* | 0.141 | 3.805 |
| chrUN_321870225-321970224 | *TRIM17* | 0.141 | 3.805 |
| chrUN_321870225-321970224 | *Unknown* | 0.141 | 3.805 |
| chrUN_321870225-321970224 | *Hist3h2bb* | 0.141 | 3.805 |
| chrUN_336770225-336870224 | *Spns3* | 0.194 | 1.909 |
| 6_32872824-33372823 | *ZFP36L1* | 0.149 | 2.322 |
| 6_32872824-33372823 | *ACTN1* | 0.149 | 2.322 |
| 6_32872824-33372823 | *DCAF5* | 0.149 | 2.322 |
| 6_32872824-33372823 | *EXD2* | 0.149 | 2.322 |
| 6_32872824-33372823 | *Galnt16* | 0.149 | 2.322 |
| 14_10144126-10244125 | *N4BP2L2* | 0.148 | 2.39 |
| 14_10144126-10244125 | *N4BP2L1* | 0.148 | 2.39 |
| 14_13744126-13844125 | *USP12* | 0.164 | 2.749 |
| 8_8600001-8700000 | *SF3B5* | 0.161 | 1.726 |
| 8_8600001-8700000 | *STX11* | 0.161 | 1.726 |

**Table S20.** **Olfactory receptor gene numbers in South American camelids, compared to the cow**

| Species | Functional gene number | Pseudogene number |
| --- | --- | --- |
| Alpaca | 591 | 259 |
| Vicuña | 586 | 271 |
| Guanaco | 602 | 248 |
| llama | 566 | 263 |
| Cow* | 1227 | 1057 |

* Niimura *et al* (2014)^2^

**Table S21**. **PCR and qPCR primers**

| Gene | Sequence 5’-3’ | Application |
| --- | --- | --- |
| *FGF5* | F:TCCTCTTGCTCAGCCACC  R:CCATTGACTTTGCCATCC | qPCR |
| *FGF5* | F:GCGAGCTATGAGCTTGTCCTTCCTCCTCCTCCT  R:GCCTCGAGTCACTCCCTGAACTTGCAGTCATCTG | PCR |
| *SOX6* | F:GGCTCGGAAGATGCGAGAAC  R:CTCAAAGCGTGTTCTTTCCTTCT | qPCR |
| *HIF1α* | F:ACCTTCATCGGAAACTCCAAAG  R:ACTGTTAGGCTCAGGTGAACT | qPCR |
| *18s rRNA* | F:GAAGGGCACCACCAGGAGT  R:CAGACAAATCACTCCACCAA | qPCR |

**Table S22**. **Blood and skin tissue expression counts for *de novo* sequenced SACs and Chinese alpaca, respectively**

| Gene | *Vicugna pacos*  (skin, *N*=17) | *Lama glama* (blood, *N*=1) | *Lama guanicoe* (blood, *N*=1) | *Vicugna vicugna* (blood, *N*=1) |
| --- | --- | --- | --- | --- |
| *ANTXR2* | 16.815 | 77.86 | 2.48 | 12.41 |
| *CORIN* | 0.396 | 1.1 | 0.42 | 0.96 |
| *EDN3* | 0.552 | 23.8 | 0.21 | 0 |
| *FGF21* | 48.333 | 0.85 | 0.21 | 0 |
| *ASIP* | 110.89 | 0 | 1.54 | 1.17 |
| *TAGLN* | 788.9 | 779.25 | 16.35 | 4.5 |
| *GNAS* | 94.18 | 1.81 | 0.18 | 0.62 |
| *CTSZ* | 124.97 | 58.35 | 71.51 | 117.2 |
| *GPER1* | 0.849 | 7.99 | 0.36 | 2.9 |
| *CS* | 48.166 | 0 | 103.56 | 0 |
| *PMEL* | 86.788 | 2.67 | 3.43 | 0.51 |
| *AGRP* | 0 | 0.99 | 0 | 0 |
| *FGF22* | 36.346 | 0 | 0 | 0 |
| *TAPBPL* | 6.996 | 42.12 | 14.8 | 18.5 |
| *LHCGR* | 0.018 | 0.71 | 0 | 0 |
| *HYOU1* | 33.435 | 45.02 | 37.75 | 93.44 |
| *MYOM1* | 2.154 | 10.14 | 1.68 | 4.6 |
| *MC5R* | 1.012 | 0.76 | 2.15 | 3.03 |
| *MC2R* | 0.049 | 0 | 0.14 | 0 |
| *MC1R* | 0 | 1.63 | 3.66 | 4.64 |
| *FGF5* | 33.05 | 0.64 | 0 | 0 |

**Supplementary Text**

S1a. Genome size estimation

To estimate the genome size of *L. guanicoe*, *V. vicugna* and *L. glama*, we first performed *k*-mer analysis on the Illumina short reads. An oligonucleotide with *k* bp refers to a *k*-mer. The *k*-mer frequencies derived from the sequencing reads follow a Poisson distribution in a given data set. Given a certain k-mer, genome size can be inferred from the total number of *k-*mers (referred to as *k*_num) divided by the k*-*mer depth (referred to as peak_depth), G=*k*_num/peak_depth. A total of 80 Gb, 214 Gb and 78 Gb clean data from short-insert size libraries were using for k-mer analysis, for each species, respectively. When the *k-*mer size was set at 17 (**Figure S1**), a total number of 67,200,000,000; 187,346,919,393 and 64,244,213, *k*-mers were generated, and the peak_depth was 26, 72 and 25, respectively. From these statistics, we estimated the three genome sizes were approximately 2.58GB, 2.60GB and 2.57Gb for *L. guanicoe*, *V. vicugna* and *L. glama* respectively, which was consistent with that from flow cytometric analysis (**Table S5**).

**S1b. *de novo* sequencing, assembly and annotation**

The paired-end libraries were constructed using paired-end kits (Illumina, USA) with average insert sizes of 170 bp, 500 bp and 800 bp. Mate-pair libraries were prepared using mate-pair kits (Illumina, USA) with average insert sizes of 2 kb, 5 kb, 10 kb, 20 kb and 40 kb. Using standard Illumina protocols, library preparation, sequencing and base calling were performed on Illumina Hiseq platforms. In total, we generated 335 Gb, 285 Gb and 262 Gb of high-quality clean data. Data filtering comprised: (1) removing reads from short insert-size libraries (<1 kb) where N constituted more than 2% of bases, and from long insert-size libraries (≥2 kb) where more than 5% of bases were poly(A); (2) removing low-quality reads from short insert-size libraries (<1 kb) when 40% or more of bases had a quality score ≤7, and from long insert-size libraries (≥2 kb) at 30%; (3) removing reads with more than 10 bp aligned to the adapter sequence (allowing ≤ 3 bp mismatch); (4) removing reads from short insert-size libraries when two reads overlapped by 10+ bp allowing for a 10% mismatch; (5) removing PCR duplicate reads; (6) removing possible contaminant reads of known bacterial and viral origin (**Table S1**).

Prior to assembly, we compiled a 17-mer frequency table for the short-insert size data (<1kb) and removed the reads with frequency lower than 10. For the data from libraries of 170 bp and 250 bp, we connected the paired-end reads into linked reads by overlapping sequences. Then *SOAPdenovo* (v2.04)^3,4^ was used to construct contigs and scaffolds for the three genomes with the same parameters (-*K* 41 -*d* 1 –*M* 2). Krskgf and Gapclose^5^ were used to fill gaps to improve the quality of genomes. The assembled genome sizes for these three individuals were 2.58, 2.6 and 2.6 Gb, respectively. Contig N50 and scaffold N50 lengths were 91.6 kb and 14.6Mb for *L. guanicoe*, 91.1 kb and 6.15Mb for *V.* *vicugna*, and 44.1 kb and 3.2Mb for *L. glama* (**Table** S**2a-c**). To investigate the quality of the genome assembly, the assembled transcriptome unigenes were mapped to the genome sequences using BLASTN^6^ (E-value < 1e-5; **Table S3**). Results showed that over 84% of the unigenes could be covered by the genome indicated its completeness. BUSCO results also indicated that >93.7% of the mammal’s orthologs could be found in the SAC genomes, also reflecting its high quality (**Table S4**). This also means that although all the SAC genomes were assembled based on NGS data, we can still assembly most of the conserved genes.

To identify transposable elements (TEs) in the assembly, we integrated *de novo* methods and homology based methods. The *de novo* prediction programs RepeatModeler^7^ (http://www.repeatmasker.org/RepeatModeler.html) and LTR-FINDER^8^ were first used to search for repetitive sequences in the assembled scaffolds, and then the repeat sequences obtained were used to construct a non-redundant repeat sequence library, with which repetitive sequences in the genome were identified using *Repeatmasker* (<http://www.repeatmasker.org>). Homology based prediction was conducted by comparing the assembly to the Repbase-18.04 using *RepeatMasker* and *RepeatProteinMask*^9^ (version 3.3.0). TEs identified by these two methods were further analysed to remove redundant elements. Overall, 32.61% 32.23% and 26.00% of the SAC genomes contained by TEs (*L.* guanicoe, *V. vicugna*, *L. glama*, respectively). Gene prediction was carried out as described in the Main Text. **Table S6a-c** show the results for each species.

**S2 Introgression analysis**

For the ABBA/BABA approach, we calculated both Patterson’s *D^10^* and, because domesticated SACs have undergone several documented demographic bottlenecks since domestication, (*f*_d_)^11^, which better accounts for genetic drift, to quantify genomic introgression. We used the frequency of the derived allele at each locus for each species instead of binary counts of fixed ABBA and BABA. The formula is as follows:

$$D\left( P_{1},P_{2},P_{3},O \right)=\frac{\sum_{i=1}^{n} \left[ \left( 1-{\overset{^}{P}}_{i1} \right){\overset{^}{P}}_{i2}{\overset{^}{P}}_{i3}\left( 1-{\overset{^}{O}}_{i} \right)-{\overset{^}{P}}_{i1}\left( 1-{\overset{^}{P}}_{i2} \right){\overset{^}{P}}_{i3}\left( 1-{\overset{^}{O}}_{i} \right) \right]}{\sum_{i=1}^{n} \left[ \left( 1-{\overset{^}{P}}_{i1} \right){\overset{^}{P}}_{i2}{\overset{^}{P}}_{i3}\left( 1-{\overset{^}{O}}_{i} \right)+{\overset{^}{P}}_{i1}\left( 1-{\overset{^}{P}}_{i2} \right){\overset{^}{P}}_{i3}\left( 1-{\overset{^}{O}}_{i} \right) \right]}$$

where *P*_1_ , *P*_2_ , *P*_3_ and *O* are the four compared taxa and ${\overset{^}{P}}_{ij}$ is the observed frequency a SNP *i* in population *j*, ${\overset{^}{O}}_{i}$ is the observed frequency of SNP *i* in the outgroup population. Under the null hypothesis of zero gene flow and random mating in the ancestral population, *D* ((*P*_1_, *P*_2_), *P*_3_), *O*) will approach zero, regardless of differences in effective population size. Hence, a significantly positive *D* is indicative of a significant excess of shared derived alleles between *P*_1_ and *P*_3_, while a significantly negative value indicates gene flow between *P*_2_ and *P*_3_. A block size of 30 Mb was selected to calculate standard errors of *D* by bootstrapping. *f*d was calculated as:

$${\overset{^}{f}}_{d}=\frac{S\left( P_{1},P_{2},P_{3},O \right)}{S\left( P_{1},P_{D},P_{D},O \right)}$$

where *P*_1_ , *P*_2_ , *P*_3_ and *O* are the four taxa compared and *P*_D_ can be either *P*_1_ or *P*_2_, which has the higher frequency of the derived allele.

**S3. Sample collection, DNA extraction and permits**

Guanaco DNA was extracted from blood samples taken from six wild-caught adults following chemical immobilization. Animals were darted while in a herd group with 10 mg Medetomidine, 300 mg Ketamine, and reversed with Atipamezole per animal^12^. DNA was extracted from skin samples from one carcass of animals form El Rosin, Chile and opportunistically from liver sample of one adult male guanaco slaughtered in Valle Chacabuco (Valchac Ltd.), Chile, under a sustainable use program authorized by the Chilean government. Blood samples were collected from five free-ranging vicuñas from Lauca, Surire and Nevado Tres Cruces Nationals Parks using 18 mg Medetomidine, 500 mg Ketamine, and reversed with Atipamezole per animal^12^, and 500 mg Tiletamine-Zolazepam with one adult vicuña form Llullaillaco National Park^13^.  Blood samples from two adult vicuñas were obtain during captures undertaken for obtaining wool in “chaku” in Huancavelica, Perú and Cieneguillas, Argentina. Samples collected from San Guillermo, Argentina were obtained from animals reared in captivity. All samples of domestic animals were obtained from the Llamas de Sur farm, Chile. Samples were collected following guidelines of the American Society of Mammalogists^14^. Specific permits were required for the Servicio Agrícola y Ganadero, SAG (Resolution Nº 5663, 2013), the Corporación Nacional Forestal, CONAF (Authorization Nº 018/2014), for granting other collection permits and helping in collecting samples (**Table** S**8**) , and from Peru to Chile by CITES Permit number 4222, granted July 15, 2003 by INRENA, Peru. All experimental protocols were approved by the Institutional Animal Care and Use Committee of Universidad del Bío-Bío. The methods were carried out in accordance with the approved guidelines. Samples were transported under CITES authorization number 6793.

**Supplementary references**

1. Wu H, Guang X, Al-Fageeh MB, Cao J, Pan S, Zhou H, et al. Camelid genomes reveal evolution and adaptation to desert environments. Nat Commun. 2014;5:5188.

2. Niimura Y, Matsui A, Touhara K. Extreme expansion of the olfactory receptor gene repertoire in African elephants and evolutionary dynamics of orthologous gene groups in 13 placental mammals. Genome Res. 2014;24:1485-96.

3. Li R, Li Y, Kristiansen K, Wang J. SOAP: short oligonucleotide alignment program. Bioinformatics. 2008;24:713-4.

4. Luo R, Liu B, Xie Y, Li Z, Huang W, Yuan J, et al. SOAPdenovo2: an empirically improved memory-efficient short-readde novoassembler. Gigascience. 2012;1:18.

5. Li R, Fan W, Tian G, Zhu H, He L, Cai J, et al. The sequence and de novo assembly of the giant panda genome. Nature. 2010;463:311-7.

6. Altschul SF, Madden TL, Schäffer AA, Zhang J, Zhang Z, Miller W, Lipman DJ.Gapped BLAST and PSI-BLAST: a new generation of protein database search programs. Nucleic Acids Res. 1997;25:3389-402

7. Smit A, Hubley R, Green P. RepeatModeler Open-1.0. 2008-2010.

8. Xu Z, Wang H. LTR_FINDER: an efficient tool for the prediction of full-length LTR retrotransposons. Nucleic Acids Res. 2007;35:W265-8.

9. Smit A, Hubley R, Green P. RepeatMasker Open-3.0. 1996-2010.

10. Durand EY, Nick P, David R, Montgomery S. Testing for ancient admixture between closely related populations. Mol Biol Evol. 2011;28:2239-52.

11. Martin SH, Davey JW, Jiggins CD. Evaluating the Use of ABBA-BABA Statistics to Locate Introgressed Loci. Mol Biol Evol. 2015;32:244-57.

12. Georoff TA, James SB, Kalk P, Calle PP, Martin-Flores M. Evaluation of Medetomidine–Ketamine–Butorphanol anesthesia with Atipamezole–Naltrexone antagonism in captive male guanacos (Lama guanicoe). J Zoo Wildlife Med. 2010;41:255-62.

13. Sarno RJ, Hunter RL, Franklin WL. Immobilization of guanacos by use of tiletamine/zolazepam. J AM Vet Med A. 1996;208:408-9.

14. Gannon WL, Sikes RS, The Animal Care and Use Committee of the American Society of Mammalogists. Guidelines of the American Society of Mammalogists for the Use of Wild Mammals in Research. J Mammal. 2007;88:809-23.
