# Supplementary material for: Genomic analysis of the domestication and post-Spanish conquest evolution of the llama and alpaca
Source: Genome Biol. 2020 Jul 2;21:159. doi: 10.1186/s13059-020-02080-6 (PMC7331169; doi:10.1186/s13059-020-02080-6)
Supplement: Supplementary file 2 — Additional file 2. Review history. [file 13059_2020_2080_MOESM2_ESM.docx]

**Peer review history**

**First round of review**

**Reviewer #1**

The results provide further detailed insight in the domestication of the Lama and Alpaca in particular about the admixture between these two species and regions that have been under selection during the domestication and subsequent selection of these species.

Overall the results are clearly presented, although the paper would benefit if the native English-speaking co-authors would critically read and edit the English used throughout the manuscript. Below under "minor comments" I have indicated several examples where the English used should be improved. These represent some obvious examples, but these are this is not an exhaustive list.

In general, the conclusion drawn by the authors are supported by the results, except for the functional studies presented in the paragraph "functional analyses". In particular the hypoxia studies in the mouse cells are not very convincing and do not support the selection of this region because of the high altitudes in the Andes. (Discussion, lines 466-470 are not very convincing and not very well supported by the results)

I am also not fully convinced about some of the variation seen in the OR genes. This is a complex gene family and very prone to misaligned reads using Illumina data, resulting in a large number of false positive SNP calls. Also, the number of genes might be underestimated in an assembly based on short read Illumina data. See e.g. lines 308-311 and lines 457-458.

Specific comments:
Lines 103-107: The authors should insert a comment about the assembler used (SOAP de novo). Also, the comment that the sequencing did result in the indicated N50 contig values is not correct. This is the result of the assembly.

Lines 107-108: RNAseq covered 82% f unique genes. This is not very clearly discussed. Also, in table S5 the "Data sets" used should be better explained. A more in-depth discussion of the BUSCO results would be better than the information provided in this table. I suggest expanding the discussion of the BUSCO analyses in the SI text.

Line 109-111: Table S7 only shows the number of aligned bases. This is not very meaningful, and the numbers shown are what is to be expected from a comparison between mammals. No information about the degree of synteny can be obtained from this information. The authors should provide the number of syntenic segments/breaks and the average size of these segments.
Furthermore, the I think it is difficult to obtain good estimates of the degree of synteny, given the fragmented nature of the assemblies.

Lines 731-733: Including the accession numbers is mandatory.

Lines 183-189: The lower admixture of the X chromosome could probably also be explained if admixture would be the result of using predominantly males. This could be checked by the analyses of the Y chromosome.

Lines 195-198: The comment about the deleteriousness of these genes in human is rather far-fetched. Furthermore, such genes are very likely to be found at other locations as well. The authors should test this and see if there is indeed a significant difference.

Minor comments:

Lines 118-119: Instead of "could have been missed" make the statement stronger like e.g. "most likely due to the short-read sequencing".

Lines 131-132: Places mentioned in the text should also be shown in figure 1b.

Figure 1b: Explain difference between open and solid dots.

Lines 173-175 and lines 176-178 "are ….. were …." Sentences are not correct.

Line 259: replace "used" by "use"

Line 266-269: Sentence not correct. Delete "were detected"

Line 280: Not clear where previous refers to "previously published results" or "results mentioned in the previous paragraph"

Lines 290-300: SNP variation is not that clearly visible from the figure. Consider mentioning the degree of variation in the text.

Line 322: A verb seems to be missing from this sentence.

Line 326: delete the words "the" and "of"

Lines 329-331: A find this a bit of a strange comment. Furthermore, it is also more discussion than results. I suggest deleting this sentence.

Line 363: Strange wording: "in the alpaca removed"

Line 368: replace "has" by "have"

Line 369: sentence is incorrect

Line 370: replace "reveal" by "reveals" and insert "which is" before "recapitulated".

Line 403: "that have showed" either "that have shown" or "that showed"

Line 404: I suggest replacing ": our" by ". Our"

**Reviewer #2**

This is an interesting study about the genomes, genetic relationships, introgression and signatures of domestication in South American camelids.

Overall, this is an extensive and mostly well-conducted study and the results may be of broad interest. However, I have a few major concerns and several minor comments regarding data analysis and presentation.

Major comments:
1.     The authors have completely ignored the most recent improved alpaca chromosome-level reference genome VicPac3.1 (Richardson et al. 2019) available at NCBI. Likewise, they have ignored the dromedary Zoo-FISH map (Balmus et al. 2007) and the alpaca cytogenetic map (Avila et al. 2014) for chromosomal assignment of the genome sequences of South American Camelids (SAC). Pseudo-chromosomal locations presented in Tables 1 and 2 are not in the agreement with previously established chromosomal homologies between human and camelids, thus confusing and misleading. For example, FGF5 has been mapped to alpaca/camelid chr2 and not to chr9 as presented in this study; EDN3 maps to alpaca/camelid chr19 and not to chr6 as shown in Tables 1 and 2, etc. Please revise to stay consistent with previous data.
2.     At places, et seems that the authors have attempted to 'bend' the published data to fit with their assumptions or hypotheses. For example, the story about EDN3 and blue-eyed, white alpacas and llamas has nothing to do with the reference Jackling et al. 2014. The referenced study is about association of white blue-eyed phenotype in alpacas with the KIT gene. Likewise, there is limited evidence justifying the author's attempt to associate down-regulation of PRMD8 with hypoxic conditions.
3.     The observed lower level of introgression in the X chromosome is intriguing but poorly explained (lines 444-451). Notion about the "cost to hybridization" requires elaboration.
4.     The authors have likely underestimated genome-wide recombination rate (Methods lines 580-585) by assuming one cross-over event per chromosome because typically, there is one cross-over event per chromosome arm.
5.     The authors have not discussed their results about the demographic expansions (lines 229-234) of SAC species. This is of interest and needs discussion.
6.     The human chromosome 4q21 open reading frame C4orf22 is cilia and flagella associated protein 299 (CFAP299) with expression restricted toward testis. Thus, rather unsuitable for expression analysis in melanocytes or skin. Likewise, FGF5 has limited expression in gall bladder and kidney, thus also not the best gene for the tissues available in this study. The authors should comment about these genes regarding their  expression results, which are clearly different compared to what is known about the expression of these genes in humans.
7.     Lines 407-414: I do not think that "coincidence" is the correct term to describe overlap between the acceleration of the decline of effective population size and the admixture pulse. They probably were two sides of the same coin associated with Spanish Conquest, and not a coincidence.
8.     English grammar, but particularly syntax, require essential editing.  

Minor comments
1.     Sections about the 4q21 region are confusing and should be presented as HSA4q21 region.
2.     Line 233: should be 100,000- 200,000; line 405: should be 2000-3000 years ago; check the manuscript for similar mistakes.
3.     Lines 297-302: one sentence extends over 6 lines. Please rephrase for clarity.
4.     Line 335: CDS should be explained at its first appearance.
5.     Lines 345-348: the sentence "However, of note …" is difficult to understand and needs rewording.
6.     Lines 382-385: The sentence starting " All methods gave ….." is long and difficult to understand. Is the event "relatively recent" or "relatively old"? Please reword for clarity.
7.     Lines 476-479: "Functional analysis…" needs a reference for "preliminary analysis of expression profiles" and rephrasing for clarity.
8.     Liness 487-490: Llamas and alpacas are not endangered species. Please reword.
9.     Methods: no information is provided about the procurement and storage of tissues for expression studies.
10.     Methods, lines 504 onwards: the authors conducted 'de novo assembly', not 'de novo sequencing'. Please correct everywhere where needed.
11.     Line 535: Section title "Resequencing of four camel species" is wrong and confusing. Please reword.
12.     Line 715: BWA-MEM is not explained.
13.     In Abbreviations, there is no consistency in using capital or small letters.
14.     Tables 1 and 2: as mentioned above, chromosomal assignments of genes in this study are in disagreement with previous comparative and cytogenetic maps and need revision.
15.     Table 1: references for EDN3 and FGF21 are wrong.
16.     Table 2: please correct the first 3 rows for the ORIGIN column; reference for the same table (Table2) is strange; the text in these 3 lines should be consistent.
17.     Table 2: ASIP function should be 'pigmentation'.
18.     Figure legends are not informative. They should be much more detailed, explaining every line (e.g., thresholds), left/right; up/down; x- and y-axes and arrows. Please essentially expand and elaborate all legends.
19.     Figure 1c: there is no explanation in text or figure legends about which K-value characterizes structure the best and why.
20.     Figure 2e: in the legend it should be 'llama' not 'alpaca'.
21.     Figure 2f: there is no arrow in the figure, though mentioned in the legend.
22.     Figure 3b: colors are too similar. Please change to more distinct colors.
23.     Figure 3c: Sequence positions in the X chromosome are inverted. All genes to the right of MID1 are located at Xp terminal region, thus close to 0 Mb. Also, there is nothing in the legend about the lower portion of Figure 3c.

**Authors’ response to reviewers**

Responses to Reviewer #1:

The paper would benefit if the native English-speaking co-authors would critically read and edit the English used throughout the manuscript.

Response: We have carried out a thorough review of the English, including the instances identified below.

In general, the conclusion drawn by the authors are supported by the results, except for the functional studies presented in the paragraph "functional analyses". In particular the hypoxia studies in the mouse cells are not very convincing and do not support the selection of this region because of the high altitudes in the Andes. (Discussion, lines 466-470 are not very convincing and not very well supported by the results)

Response: It was not clear to us whether Referee 1 was concerned about our use of the mouse model, and experimental manipulation of atmospheric oxygen, as a proxy for hypoxic conditions at high altitude or whether the referee found our functional results unconvincing. In the case of the former, we could remove this section entirely, depending on the opinion of the Editor and the reviewer, however our preference is to retain this analysis and to this end we have carried out additional functional analysis of the regulatory effects of hypoxic condition on the mRNA expression of SOX6 and HIF1α in alpaca melanocytes. We show that PRDM8 is down-regulated under hypoxic conditions and that this is mirrored by decreased expression of SOX6 and HIF1α (Results, Page 19, Lines 478-488; Fig. 4f).

We added a section in the discussion: "Our qPCR results showed that mRNA expression levels for the genes SOX6 and HIF1α are similarly downregulated. SOX6 is one of the targets of PRDM8 in the retina and its expression is controlled by PRDM8 (Allen, 2008). The response of HIF1α may therefore be indirect via the expression of the transcription factors SOX6 (and/or SOX5 and SOX9), depending on levels of atmospheric oxygen availability (Khan et al, 2007). Under hypoxia, mitochondrial O2 consumption is inhibited or intracellular Fe and PHD (prolyl hydroxylase) activity increases, which decreases the expression of HIF1α (Ke and Max, 2006). Therefore, FGF5, ANTXR2 and C4orf22 may play a key role in regulating hypoxia stress, while PRDM8 is a novel gene associated with hypoxic adaptation, the expression of which may be mirrored by decreased expression of SOX6 and HIF1α”. Following these new results, we have revised the text (Page 19, Lines 478-487).

I am also not fully convinced about some of the variation seen in the OR genes. This is a complex gene family and very prone to misaligned reads using Illumina data, resulting in a large number of false positive SNP calls. Also, the number of genes might be underestimated in an assembly based on short read Illumina data. See e.g. lines 308-311 and lines 457-458.

Response: We agree that it is possible that misaligned reads from Illumina data may result in false positive SNP calls. However, previous research has shown that over 95% of the SNPs identified in the OR genes are consistent between Illumina and Sanger sequencing results (e.g. Trimmer et al., 2019). Therefore, we contend that the large majority of our SNPs in the OR genes should be authentic.

We also agree that the number of OR genes might be underestimated in an assembly based on short read Illumina data. As discussed by Li et al. (2016), there may be as much as a 10% underestimation in whole genome short gun assemblies (WGSA). However, when we compare our data with, for example, the cattle reference genome, which was also WGS assembled, we still find an approximately 50% contraction in SAC OR genes, and thus we believe that this observed contraction is authentic. Further, we have updated our OR gene annotation using our previously published pipeline (Zhan et al. 2013, Nature Genetics; Table S20). Our predicted OR copy number did show a slight increase (491 vs 591 for alpaca, 583 vs 602 for Guanaco, 552 vs 566 for llama), but the overall observation remains.

We have therefore revised the text to state “We also found that the number of SAC functional olfactory receptor (OR) genes was substantially lower than for other domestic livestock (approximately 50% of cattle and horse, at 566-602, Additional file 1: Table S20).”(Page 13, Lines 317-319).


Specific comments:
Lines 103-107: The authors should insert a comment about the assembler used (SOAP de novo). Also, the comment that the sequencing did result in the indicated N50 contig values is not correct. This is the result of the assembly.

Response: We have added a comment on the assembly software and revised the sentences, please see Line 105.

Lines 107-108: RNAseq covered 82% f unique genes. This is not very clearly discussed. Also, in table S5 the "Data sets" used should be better explained. A more in-depth discussion of the BUSCO results would be better than the information provided in this table. I suggest expanding the discussion of the BUSCO analyses in the SI text.

Response: We have added an explanation of the RNAseq data, please see Line 108. Additionally, we have added the BUSCO results and discussion in the SI text, and SI Table S4.

Line 109-111: Table S7 only shows the number of aligned bases. This is not very meaningful, and the numbers shown are what is to be expected from a comparison between mammals. No information about the degree of synteny can be obtained from this information. The authors should provide the number of syntenic segments/breaks and the average size of these segments. Furthermore, the I think it is difficult to obtain good estimates of the degree of synteny, given the fragmented nature of the assemblies.

Response: We have restated the results according to the reviewer’s suggestion, please see the new version of Table S6a.

Lines 731-733: Including the accession numbers is mandatory.
Response: We have now added “The datasets generated and analyzed during the current study are available in the NCBI’s GenBank PNXU00000000, PNXV00000000, PNXW00000000. The SAC whole-genome shotgun project has been deposited at the GenBank under the accession number PRJNA612032. Other results are included in this published article and its Additional material files.” (Page 31, Lines 769-772)

Lines 183-189: The lower admixture of the X chromosome could probably also be explained if admixture would be the result of using predominantly males. This could be checked by the analyses of the Y chromosome.

Response: Thanks for this important comment. We conducted admixture analysis in Y chromosome as suggested. Briefly, we mapped the reads of two selected individuals (one male and one female) onto our assembled scaffolds and identified Y fragments based on the sequence depth ratio (male/female ≥ 2). We next connected the identified Y fragments together and used the assembled sequence as our Y chromosome sequence in the following analyses. The length of the Y chromosome sequence connected was 2,672,492 bp. We then selected the male individuals (N = 14) from our study and mapped their reads onto the Y chromosome. GATK pipeline was used to call SNPs as we used in study. To obtain the final SNP dataset, we filtered the sites with low mapping quality, missing and heterozygous genotypes. Finally, we obtained a total of 45,068 SNPs. We further ran ADMIXTURE and plotted a haplotype heatmap. The results showed that there is almost no admixture between the llama and alpaca in Y chromosome, rejected the hypothesis that the lower admixture detected on the X chromosome is an artefact of using predominantly males.

Lines 195-198: The comment about the deleteriousness of these genes in human is rather far-fetched. Furthermore, such genes are very likely to be found at other locations as well. The authors should test this and see if there is indeed a significant difference.

Response: We have now revised it as “The four regions with low introgression contained genes with known deleterious or lethal X-linked effects in humans or mouse (25 genes in low introgression regions and 28 in high regions). For example, the deleterious mutations in the PTCHD1 (Patched domain containing 1) gene have been described in male patients with X-linked Autism spectrum disorder (ASD) and/or intellectual disability (ID) (Noor et al. 2010; Torrico et al. 2015), which further been found the deficiency of PTCHD1 could also induces excitatory synaptic and cognitive dysfunctions in mouse (Ung et al. 2018)”. (Pages 8, Lines 198-204). Furthermore, we obtained the genes sequences from four representative “low” introgression regions on the X chromosome and blasted them against the autosomes. The results showed that all of them to be single copy genes.

Reference cited:
Noor A, Whibley A, Marshall CR, Gianakopoulos PJ, Piton A, Carson AR, et al. Disruption at the PTCHD1 locus on Xp22. 11 in Autism spectrum disorder and intellectual disability. Sci Transl Med. 2010;2:49-68.
Torrico B, Fernàndez-Castillo N, Hervás A, Milà M, Salgado M, Rueda I, Buitelaar JK, Rommelse N, Oerlemans AM, Bralten J, et al. . Contribution of common and rare variants of the PTCHD1 gene to autism spectrum disorders and intellectual disability. Eur J Hum Genet. 2015;23:1694-1701.
Ung DC, Iacono G, Méziane H, Blanchard E, Papon MA, Selten M, van Rhijn JR, Montjean R, Rucci J, Martin S, et al. Ptchd1 deficiency induces excitatory synaptic and cognitive dysfunctions in mouse. Mol Psychiatry. 2018;23:1356-1367.

Minor comments:

Lines 118-119: Instead of "could have been missed" make the statement stronger like e.g. "most likely due to the short-read sequencing”.

Response: We have revised it as “although such sequences were difficult to assemble most likely due to the limitations of short-read sequencing.” (Page 5, Lines 120-121)

Lines 131-132: Places mentioned in the text should also be shown in figure 1b.
Response: Thank you for your suggestion. We have revised it accordingly. In addition, we also plotted the detailed information of sequenced individuals on the map (Additional file1: Fig S2).

Figure 1b: Explain difference between open and solid dots.
Response: We added this sentence in the legend of Figure 1,“Map with current distributions of SAC and sample location. Open and solid dots indicate sampled localities of vicunas and guanacos respectively”.

Lines 173-175 and lines 176-178 "are ….. were …." Sentences are not correct.
Response:
We have modified these sentences as: “There were 87 windows (comprising 101 genes) detected in common by both methods.” and “using the same approach, we identified 247 high alpaca ancestry regions using LAI or fd, comprising 335 and 287 genes, respectively (Additional file 1: Table S11; Figure S4) with 113 regions (192 genes) detected in common (Additional file 1: Table S12), which were also investigated for GO terms.” (Page 7, Lines 176-181)


Line 259: replace "used" by "use"
Response: Modified as suggested. (Page 10, Line 265)

Line 266-269: Sentence not correct. Delete "were detected”
Response: Deleted as suggested. (Page 11, Line 272)


Line 280: Not clear where previous refers to "previously published results" or "results mentioned in the previous paragraph”
Response: We mean it previous paragraph. We change these words as “domestication analysis”. (Page 11, Line 287)


Lines 290-300: SNP variation is not that clearly visible from the figure. Consider mentioning the degree of variation in the text.
Response: Revised as suggested. We calculated the the genetic diversity, and used it as the degree of variation. (Page 12, Lines 304-307)

Line 322: A verb seems to be missing from this sentence.
Response: We have revised it as: “we found clear evidence of introgression from llama to alpaca”. (Page 13, Lines 317-319)

Line 326: delete the words "the" and “of"
Response: We have deleted them. (Page 13, Line 331)

Lines 329-331: A find this a bit of a strange comment. Furthermore, it is also more discussion than results. I suggest deleting this sentence.
Response: We deleted it as suggested.

Line 363: Strange wording: "in the alpaca removed"
Response: We revised it as “When we recalculated the phylogeny with the introgression segments removed from the alpaca genome”. (Page 15, Lines 365-366)


Line 368: replace "has" by “have"
Response: We have corrected it. (Page 15, Line 371)

Line 369: sentence is incorrect
Response: we revised this sentence as “It remains possible, however, that residual introgression segments which we were unable to reliably detect using LAI have influenced the shape of the phylogeny and could underly the separation of alpaca from mensalis (Figure 1a)”. (Page 15, Lines 370-373)
Line 370: replace "reveal" by "reveals" and insert "which is" before “recapitulated".
Response: Done as suggested. (Page 15, Lines 373-374)

Line 403: "that have showed" either "that have shown" or "that showed”
Response: We have revised it as “that have shown” (Page 16, Line 409)

Line 404: I suggest replacing ": our" by ". Our"
Response: We have revised it as “and our". (Page 16, Line 410)


Responses to Reviewer #2
This is an interesting study about the genomes, genetic relationships, introgression and signatures of domestication in South American camelids.

Overall, this is an extensive and mostly well-conducted study and the results may be of broad interest. However, I have a few major concerns and several minor comments regarding data analysis and presentation.

Major comments:
1. The authors have completely ignored the most recent improved alpaca chromosome-level reference genome VicPac3.1 (Richardson et al. 2019) available at NCBI. Likewise, they have ignored the dromedary Zoo-FISH map (Balmus et al. 2007) and the alpaca cytogenetic map (Avila et al. 2014) for chromosomal assignment of the genome sequences of South American Camelids (SAC). Pseudo-chromosomal locations presented in Tables 1 and 2 are not in the agreement with previously established chromosomal homologies between human and camelids, thus confusing and misleading. For example, FGF5 has been mapped to alpaca/camelid chr2 and not to chr9 as presented in this study; EDN3 maps to alpaca/camelid chr19 and not to chr6 as shown in Tables 1 and 2, etc. Please revise to stay consistent with previous data.

Response: Thanks for suggesting this. We aligned our assembled scaffolds against the VicPac3.1 (which was published after we finished the assembly for the first version of this paper) using LAST (Kiełbasa et al. 2011) and obtained the chromosome information for each scaffold. However, as confirmed by contact with the authors, there is no ordering information for scaffolds within each chromosome of their assembly. Therefore, in this revision we joined the scaffolds together after sorted them by length within each VicPac3.1 chromosome. For unmapped scaffolds, we connected them together after sorted by length and classified them as “ChrUN”. In total, ~2.1 Gb of assembled scaffolds in our study could be mapped to chromosomes in VicPac3.1 and ~499.7 Mb were classified as “ChrUN”, the same level as those reported by VicPac3.1. Based on the obtained chromosome information, we revised the chromosome and position information in Fig 3a, Fig 4a-d, Fig S4, S7-12, Table1, Table2, S11-12, and Table S14-19.

In addition, we also redid some of analysis of demographic history (PSMC, MSMC and SNeP) that could potentially be influenced by the scaffold assembly order. The overall result of these analyses remains unchanged, and we have now incorporated the refined figures and have slightly amended the text (Pages 9-10, Lines 235-248).

2. At places, et seems that the authors have attempted to 'bend' the published data to fit with their assumptions or hypotheses. For example, the story about EDN3 and blue-eyed, white alpacas and llamas has nothing to do with the reference Jackling et al. 2014. The referenced study is about association of white blue-eyed phenotype in alpacas with the KIT gene. Likewise, there is limited evidence justifying the author's attempt to associate down-regulation of PRMD8 with hypoxic conditions.

Response: In both alpacas and llamas, white individuals with blue eyes are often deaf, the phenotype of which is known to be regulated by KIT gene (Jackling et al. 2014). However, in the phenotypically similar Waardenburg syndrome in humans, these phenotypes are associated with EDN3, SOX10 and EDNRB (Segun-Busar et al. 2008). While the linkage of these phenotypes to EDN3 in SAC was previously unsuspected, studies have suggested EDN3 is linked to white pelage in wild cats (Xu et al. 2017) and the co-occurrence of deafness (congenital deafness) and white pigmentation is a common observation in domestic animals (Strain, 2015). We have now clarified this in the text. (Page 12, lines 309-315)

Reference cited:
Jackling FC, Johnson WE, Appleton BR. The genetic inheritance of the blue-eyed white phenotype in alpacas (Vicugna pacos). J Hered. 2014;105:847-57.
Xu X, Dong GX, Schmidt-Küntzel Anne, et al. The genetics of tiger pelage color variations. Cell Res. 2017;954-957.
Segun-Busari S, Ayodele O, Dunmade AD, Afolabi O, Omokanye H, Owoeye JF. Blue eyes syndrome and deafness: the need for early audiological screening. Nigerian Journal of Otorhinolaryngology. 2018; 16: 25-8.
Strain GM. The genetics of deafness in domestic animals. Front. Vet Sci. 2015;2:1-20.

The association of PRDM8 with hypoxia-driven selection is, to our knowledge, a novel finding. To investigate the potential mechanism, we carried out qPCR for analysis of the regulatory effects of hypoxic condition on the mRNA expression of SOX6 and HIF1α in alpaca melanocytes (see above). (Pages 19, Lines 478-483 and Page 29, Lines 741-746).

3. The observed lower level of introgression in the X chromosome is intriguing but poorly explained (lines 444-451). Notion about the "cost to hybridization" requires elaboration.

Response: In this part, we have revised the text as “while admixture has been bidirectional, the generally lower levels of introgression in the X-chromosome suggests a potential barrier to hybridization which might have further impacted on effective population size trajectory after the Conquest”. (Pages 18, Lines 451-454)

4. The authors have likely underestimated genome-wide recombination rate (Methods lines 580-585) by assuming one cross-over event per chromosome because typically, there is one cross-over event per chromosome arm.
Response: We did consider this issue, but as almost all chromosomes in alpaca are acrocentric (with the exception of Chr 14, 16 and X) (Avila et al. 2014). We did not feel it was necessary to modify the recombination rate in this case. We also note that this is at best a very imprecise quantification, nonetheless, our primary results are robust to significant perturbation.

Reference cited:
Avila F, Baily MP, Perelman P, Das PJ, Pontius J, Chowdhary R, Owens E, Johnson WE, Merriwether DA, Raudsepp T. A comprehensive whole-genome integrated cytogenetic map for the alpaca (Lama pacos). Cytogenet Genome Res. 2014;144:196-207.

5. The authors have not discussed their results about the demographic expansions (lines 229-234) of SAC species. This is of interest and needs discussion.

Response: The demographic history of SACs since the divergence of Lama and Vicugna c 2.5 M years before present shows evidence for a strong expansion, albeit earlier for the vicuña, with the expansion of the genus Lama coinciding with the Mindel-Riss/Holstein interglacial period followed by a decline over the last 200,000 years, which has continued largely to the present day (Additional file 1: Figure S5 and S6). The difference between the two genera may reflect their altitudinal partitioning and reliance of the guanaco on multiple Andean habitats, which would have opened up during this interglacial period, potentially allowing the population to expand. We added the discussion in Lines 401-408.

It is also noted that the main aim of our paper was not to explore ancient changes in Ne (which was covered in the original study with the alpaca reference genome) and we focus on recent changes in Ne during the time period covered by domestication.

6. The human chromosome 4q21 open reading frame C4orf22 is cilia and flagella associated protein 299 (CFAP299) with expression restricted toward testis. Thus, rather unsuitable for expression analysis in melanocytes or skin. Likewise, FGF5 has limited expression in gall bladder and kidney, thus also not the best gene for the tissues available in this study. The authors should comment about these genes regarding their expression results, which are clearly different compared to what is known about the expression of these genes in humans.

Response: We added a comment on the function of FGF5 as follows: FGF5 was originally reported as a human oncogene (Zhan et al. 1987) , associated with a number of cancers (Ghassemi et al, 2017), angiogenesis in human aortic endothelial cells (Seo et al, 2016) and trichomegaly in humans (Higgins et al, 2014). (Page 20, Lines 487-489).

The expression of C4orf22 in mouse heart, liver, spleen, lung, kidney, testis and brain has been studied and although abundant expression of C4orf22 was found in testis, trace expression was also observed in lung (Li et al, 2019). Therefore, we rephrased the text as follows: “C4orf22 is expressed in human neural progenitor cells expressing MEF2CA (Chan et al, 2015)”. (Page 20, Lines 489).

References cited:
Zhan X, Culpeppe, A, Reddy M, Loveless J, Goldfarb M. Human oncogenes detected by a defined medium culture assay. Oncogene. 1987; 1: 369-76.
Seo HR , Jeong HE , Joo HJ , Choi SC, Park CY, Kim JH, Choi JH, Cui LC, Hong SJ, Chung S, Lim DS. Intrinsic FGF2 and FGF5 promotes angiogenesis of human aortic endothelial cells in 3D microfluidic angiogenesis system. Sci Rep. 2016; 6:28832.
Higgins CA, Petukhova L, Harel S, Ho YY, Drill E, Shapiro L, Wajid M, Christiano AM. FGF5 is a crucial regulator of hair length in humans. Proc Natl Acad Sci U S A. 2014;111:10648-53.
Ghassemi S, Vejdovszky K, Sahin E, et al. Fgf5 is expressed in melanoma and enhances malignancy in vitro and in vivo. Oncotarget. 2017; 8:87750-62.
Li H, Dai Y, Luo Z, Nie D. Cloning of a new testis-enriched gene c4orf22 and its role in cell cycle and apoptosis in mouse spermatogenic cells. Mol Biol Rep. 2019; 46:2029-38.
Chan SF, Huang X, McKercher SR, Zaidi R, Okamoto SI, NakanishiN, Lipton SA. Transcriptional profiling of MEF2-regulated genes in human neural progenitor cells derived from embryonic stem cells. Genomics Data. 2015; 3:24-7.

7. Lines 407-414: I do not think that "coincidence" is the correct term to describe overlap between the acceleration of the decline of effective population size and the admixture pulse. They probably were two sides of the same coin associated with Spanish Conquest, and not a coincidence

Response: As suggested we revised the text as “Interestingly, a coincident acceleration in this decline was detected approximately 110 generations ago using NeS (Fig. 2f), very close to the date estimate for the admixture pulse and in line with the recorded decline following the Spanish Conquest. So, taken together, the admixture tract and SNeP analysis point to a single event in both species occurring 100-110 generations ago.” (Page 17, Lines 416-417)

8. English grammar, but particularly syntax, require essential editing.

Response: We have carried out a thorough review of the English by one of the corresponding authors.

Minor comments
1. Sections about the 4q21 region are confusing and should be presented as HSA4q21 region.
Response: Revised as suggested. (Page 18, Line 459).

2. Line 233: should be 100,000- 200,000; line 405: should be 2000-3000 years ago; check the manuscript for similar mistakes.

Response: Corrected as suggested. (Page 9, Line 238 and Page 17, Line 412).

3. Lines 297-302: one sentence extends over 6 lines. Please rephrase for clarity.
Response: We revised it as: “In contrast, for the EDN3 gene, while the alpaca origin of the domestic haplotype is clear (Fig. 4c), supporting its vicuña origin, there is no evidence for a loss of diversity between vicuña and alpaca (EDN3 does not feature in either the FST or XP-EHH candidate list; Fig. 4a) or at the stage of admixture (alpaca – llama, where LAI and fd scores were 0.400 and 0.664, respectively, suggestive of recent artificial selection for this phenotype in the domestic forms (white fleece, blue eyes21;Fig 4e)”. (Page 12, Lines 304-309).

4. Line 335: CDS should be explained at its first appearance.

Response: We added the full name “coding sequence”. (Page 13, Line 337)

5. Lines 345-348: the sentence "However, of note …" is difficult to understand and needs rewording.

Response: We revised it as “We found very high expression levels for transgelin (7x higher compared to all other genes) in alpaca and llama skin (expressed at a similar level to alpaca, although in blood, while being effectively absent from the wild species). (Page 14, Lines 346-348)

6. Lines 382-385: The sentence starting "All methods gave ….." is long and difficult to understand. Is the event "relatively recent" or "relatively old"? Please reword for clarity.

Response: We revised it as: “All methods gave similar results but the LAI analysis of admixture tract lengths (Fig. 2c and 2e) allowed us to conclude that a single major event occurred contemporaneously for both species and is relatively recent, occurring just over 100 generations ago, consistent with the low variance in ancestry components detected”. (Page 15, Lines 384-388)

7. Lines 476-479: "Functional analysis…" needs a reference for "preliminary analysis of expression profiles" and rephrasing for clarity.

Response: We have revised the sentence as “Functional analysis of the FGF5 gene in suri and huacaya alpacas has previously shown its potential importance in regulating fleece length (Pallotti et al, 2018) and preliminary analysis of expression profiles in domestic SACs have shown the potential importance of a novel QTL for fine fiber production in merino sheep30”. (Page 20, Lines 490-493).

Reference cited: Pallotti S, Pediconi D, Subramanian D, Molina MG, Antonini M, Morelli MB, Renieri C, La TerzaA. Evidence of post-transcriptional read through regulation in FGF5 gene of alpaca. Gene, 2018; 647: 121-128.

8. Lines 487-490: Llamas and alpacas are not endangered species. Please reword.

Response: We have reworded it as “species management or conservation”. (Page 20, Line 503).

9. Methods: no information is provided about the procurement and storage of tissues for expression studies.

Response: We added the information as follows: “Six healthy 2 year-old longwool (suri) alpacas (3:3) and shortwool (huacaya) alpacas (3:3) were selected for mRNA and protein expression testing using the skin samples. A skin section (diameter 3 cm) from the neck was surgically obtained and immediately stored in liquid nitrogen”. (Page 29, Lines 716-719)

10. Methods, lines 504 onwards: the authors conducted 'de novo assembly', not 'de novo sequencing'. Please correct everywhere where needed.

Response: We have corrected them wherever necessary. (Page 21, Lines 519-520).

11. Line 535: Section title "Resequencing of four camel species" is wrong and confusing. Please reword.

Response: We corrected it as “Resequencing of individuals of four camelid species”. (Page 22, Line 550).

12. Line 715: BWA-MEM is not explained.

Response: The full name, Burrows-Wheeler Alignment MEM, is added accordingly (Page 23, Line 570).

13. In Abbreviations, there is no consistency in using capital or small letters.

Response: We have formatted the capital and small letters. (Pages 29 and 30)

14. Tables 1 and 2: as mentioned above, chromosomal assignments of genes in this study are in disagreement with previous comparative and cytogenetic maps and need revision.

Response: Thank you for this important comment. We have corrected the chromosomal assignments of genes in Table1 and Table2 after our alignment analysis (Pages 48 and 49).

15. Table 1: references for EDN3 and FGF21 are wrong.

Response: The reference for FGF21 is corrected as“ Wang R, Chen T, Zhao B, Fan R, Ji K, Yu X, Wang X, Dong C. FGF21 regulates melanogenesis in alpaca melanocytes via ERK1/2-mediated MITF downregulation. Biochemical and Biophysical Research Communications. 2017; 490:466-71”.

We revised the text about EDN3 as “the Blue-eyed, white haired phenotype in Waardenburg syndrome of humans”. The reference is listed as “Segun-Busari S, Ayodele O, Dunmade AD, Afolabi O, Omokanye H, Owoeye JF. Blue eyes syndrome and deafness: the need for early audiological screening. Nigerian Journal of Otorhinolaryngology. 2018; 16: 25-8. ”. (Pages 48 and 49).


16. Table 2: please correct the first 3 rows for the ORIGIN column; reference for the same table (Table2) is strange; the text in these 3 lines should be consistent.

Response: Thank you for your suggestions. We have revised them accordingly in the new Table 2. (Pages 49)

17. Table 2: ASIP function should be ‘pigmentation'.

Response: Corrected as suggested. (Pages 48 and 49)

18. Figure legends are not informative. They should be much more detailed, explaining every line (e.g., thresholds), left/right; up/down; x- and y-axes and arrows. Please essentially expand and elaborate all legends.

Response: We have refined the legends as suggested (Pages 45-47).

19. Figure 1c: there is no explanation in text or figure legends about which K-value characterizes structure the best and why.

Response: In Lines 150-151, we have mentioned the optimal grouping to be k = 2 based on the cross-validation admixture analysis.

20. Figure 2e: in the legend it should be 'llama' not ‘alpaca'.

Response: Corrected as suggested. (Page 46, Line 1120)

21. Figure 2f: there is no arrow in the figure, though mentioned in the legend.

Response: We have added the arrow to Figure 2f.

22. Figure 3b: colors are too similar. Please change to more distinct colors.

Response: We have changed the colors as suggested.

23. Figure 3c: Sequence positions in the X chromosome are inverted. All genes to the right of MID1 are located at Xp terminal region, thus close to 0 Mb. Also, there is nothing in the legend about the lower portion of Figure 3c.

Response: We have corrected the sequence positions and added the details into the legend.

**Second round of review**

**Reviewer #1**

The authors have sufficiently addressed most of my comments made in my earlier report except for my comments related to the SNP diversity within the OR genes and the NCBI accession numbers.

In line 463-464 the authors still conclude that "to a complex mosaic of SNP diversity across the OR families". The SNP diversity seen in these genes strongly contrasts to the other genes in this region, and the authors have not ruled out that this is the result of misaligned sequence reads. I think the authors should make a reservation that they cannot rule out such an artefact when commenting this observation.

I think that a reviewer should be able to access the raw data on which the conclusion of the manuscript is based.  Providing non existing GenBank accession numbers (PNXU00000000, PNXV00000000, PNXW00000000) and referring to an accession number (PRJNA612032) that seems to be hidden from the public (the accession number did not retrieve any hits at NCBI) is not acceptable.

I have a few additional minor comments:

The authors refer to the HSA4q21 locus in lines 57, 459 and 739. This is a region on human chromosome 4q and not a region introgressed in alpaca and llama. The authors should refer to this region in alpaca and llama as the region that is syntenic to HSA4q21.

Line 108: replace "were used" by "was used"

Line 201-202: The final part of this sentence ("which further …….. in mouse") is not correct.

Line 418: replace "estimate" by "estimated".

**Reviewer #2**

The authors have adequately responded to all comments and  thoroughly revised the manuscript. No further comments.

**Response to reviewers**

 Responses to Reviewer #1: 
The authors have sufficiently addressed most of my comments made in my earlier report except for my comments related to the SNP diversity within the OR genes and the NCBI accession numbers. In line 463-464 the authors still conclude that "to a complex mosaic of SNP diversity across the OR families". The SNP diversity seen in these genes strongly contrasts to the other genes in this region, and the authors have not ruled out that this is the result of misaligned sequence reads. I think the authors should make a reservation that they cannot rule out such an artefact when commenting this observation. 
Response: We agree with the reviewer and put a statement that “though we cannot rule out the possibility that some OR SNPs may be artefacts due to misaligned sequences” (Line 468-469).

 I think that a reviewer should be able to access the raw data on which the conclusion of the manuscript is based. Providing non existing GenBank accession numbers (PNXU00000000, PNXV00000000, PNXW00000000) and referring to an accession number (PRJNA612032) that seems to be hidden from the public (the accession number did not retrieve any hits at NCBI) is not acceptable. 
Response: Sorry for this. We have already emailed GenBank to release the data. Please check them with the accession numbers, PRJNA427832, PRJNA421373, PRJNA427644, PRJNA636766 and PRJNA612032. The first four bioprojects have been already released on 2 June 2020, the last bioproject will be released on 8 June 2020.

 Minor comments:  
The authors refer to the HSA4q21 locus in lines 57, 459 and 739. This is a region on human chromosome 4q and not a region introgressed in alpaca and llama. The authors should refer to this region in alpaca and llama as the region that is syntenic to HSA4q21.
Response: Thanks for this. We have corrected the locus name and revised the text accordingly (Line 60, 481, and 744).  

Line 108: replace "were used" by "was used"
Response: done.  

Line 201-202: The final part of this sentence ("which further …….. in mouse") is not correct.
Response: We have corrected the sentence as “which further been found that the deficiency of PTCHD1 could also induce excitatory synaptic and cognitive dysfunctions in mouse”. (Line 205).  Line 418: replace "estimate" by "estimated". 
Response: done as suggested.   

Reviewer #2: The authors have adequately responded to all comments and thoroughly revised the manuscript. No further comments.
Reponses: Thanks for your positive comments.
